# A taxonomic revision of *Curarea* Barneby & Krukoff (Menispermaceae)

**DOI:** 10.3897/phytokeys.100.21828

**Published:** 2018-06-21

**Authors:** Rosa del C. Ortiz

**Affiliations:** 1 Missouri Botanical Garden, 4344 Shaw Blvd., St. Louis, Missouri 63110, USA

**Keywords:** Arrow poison, curare, dioecious liana, neotropical clade, Ranunculales

## Abstract

A monograph of *Curarea*, a neotropical genus in the plant family Menispermaceae, is presented. *Curarea* is distinguished from related genera by the combination of staminate flowers with sepals in two whorls and pistillate flowers with three petals, three carpels and usually elongated carpophores bearing three sessile drupelets. Nine species are recognised, amongst them two new to science, *C. gentryana* from Ecuador and *C. barnebyana*, from Ecuador and Peru. Additionally, two new combinations, *C. iquitana* and *C. tomentocarpa*, are proposed for distinct taxa recovered in a multivariate analysis of quantitative characters of the broadly distributed and morphologically variable *C. toxicofera*. The anatomy and morphology of species in the genus is documented, identification key, species descriptions, distribution maps and a preliminary conservation assessment for all accepted species are also provided. Of the nine species recognised here, *C. barnebyana* is assigned a preliminary status of Vulnerable, *C. crassa* (known only from the coastal Atlantic Forest in Brazil) and *C. gentryana* (endemic to western Ecuador) are both assigned a preliminary status of Endangered.

## Introduction

**Figure 1.**
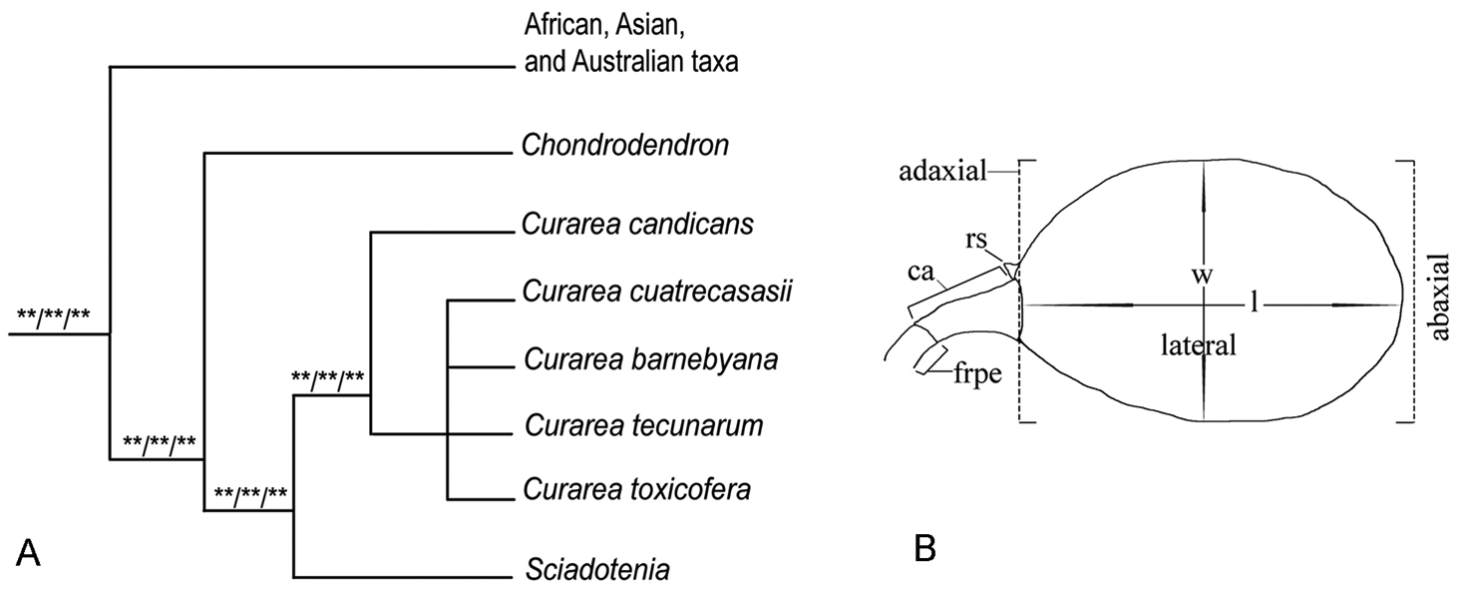
Schematic representation of affinities and drupelet: **A** phylogenetic affinities of *Curarea* and related taxa, modified from Ortiz et al. 2016, three asterisks (***) indicate 100% support in all analyses in the referred study **B** schematic illustration of a *Curarea* drupelet showing the length and width planes measured, ca./abbrev> = carpophore, frpe = fruiting pedicel, rs = remnant of style.

*Curarea* Barneby & Krukoff of the plant family Menispermaceae, tribe Tiliacoreae, is a genus of understory or canopy dioecious lianas that are widely distributed in the humid forests of tropical America, mostly at low elevation from eastern Brazil to Costa Rica in Central America. Within Tiliacoreae, *Curarea* shares with remaining members of the tribe endocarps ornamented by longitudinal ribs, seeds hippocrepiform and without endosperm and fleshy cotyledons (Ortiz et al. 2016). *Curarea* was segregated from *Chondrodendron* Ruiz & Pav. to include species that differed in several floral and carpological features (Barneby and Krukoff 1971). In *Curarea*, the calyx of staminate flowers is composed of two whorls of three, scarcely fleshy and greenish sepals, these being variously described as sepaloid scales (Barneby and Krukoff 1971: 7) or as sepals (Barneby 1996). In *Chondrodendron*, the calyx is formed by four or more whorls of three, membranous sepals that are cream coloured and, hence, described as petaloid sepals (Barneby and Krukoff 1971: 7). Staminate flowers of both genera have 6 petals that are membranous and much shorter than the innermost sepals (Table 1). Pistillate flowers of *Curarea* have only 3 petals and 3 carpels, whilst those of *Chondrodendron* have 6 petals and 6 carpels. The drupelets of both genera have narrow supportive structures, but in *Curarea*, these are elongate carpophores that bear atop sessile fruits, while in *Chondrodendron* the fruits are conspicuously stipitate (Table 1). The distinct nature of *Curarea* is also supported in a family-wide phylogenetic analysis of chloroplast markers (Ortiz et al. 2016), where it is recovered as monophyletic and sister to *Sciadotenia* Miers, in a neotropical clade that also includes *Chondrodendron* (Fig. 1A; Ortiz et al. 2016). Within this neotropical clade, *Curarea* shares with *Sciadotenia* sessile drupelets borne on narrow elongated carpophores bearing six carpels in *Sciadotenia* and only three in *Curarea*. The two whorls of sepals in the staminate flowers distinguish *Curarea* from *Chondrodendron* and *Sciadotenia*, both of which have 3–7 whorls of sepals.

**Table 1.** Main features of *Curarea* and *Chondrodendron*.

Genus	♂ Flowers			♀ Flowers		Drupelets	Carpophore
	Whorls of sepals	Sepal texture	Sepal colour	Petal whorls	Carpel #		
*Curarea*	2	fleshy	greenish	1	3	sessile	present
*Chondrodendron*	≥ 4	membranous	yellowish	2	6	stipitate	absent

When first described, *Curarea* included four species: *C. candicans*, *C. cuatrecasasii*, *C. tecunarum* and *C. toxicofera*, with *C. toxicofera* designated as the type species of the genus (Barneby and Krukoff 1971). While *C. cuatrecasasii* and *C. tecunarum* were newly described, both *C. candicans* and *C. toxicofera* were transferred from *Chondrodendron*. Another species was later added to the original four with the recognition of *C. crassa*, an endemic new species from the Brazilian Atlantic Forest (Barneby 1996). Although the etymology of the generic epithet was never provided, the name *Curarea* doubtlessly refers to curare, the South American arrow-poison for which species of *Curarea* provide one of the ingredients.

In this first treatment of *Curarea* (Barneby and Krukoff 1971), which was also the most comprehensive one at that time, *C. toxicofera* was broadly interpreted and included entities formerly described as various taxa, rendering it a large complex with unclear patterns of morphological variation. However, subsequent works generally followed that of Barneby and Krukoff (1971) and used the five species they recognised in treatment of local, regional and national floras, e.g. Flora of Surinam (Jansen-Jacobs 1976); Flórula de Las Reservas Biológicas de Iquitos, Perú (Ortiz 1997); Flora of Ecuador (Ott 1997); Flora de Nicaragua (Ortiz 2001), amongst others. Seven species were later recognised in the most recent, taxonomic account of the genus (Ortiz-Gentry 2000) but *Curarea toxicofera* remained broadly circumscribed.

On the basis of shared macro- and micromorphology, Ortiz-Gentry (2000) suggested that there were two species groups in *Curarea*. These were not recovered in a later family-wide molecular study (Ortiz et al. 2016) where it was found that *C. candicans* – an outlier in the study of Ortiz-Gentry (2000) – was sister to the remaining species of the genus. Species boundaries in most *Curarea* are relatively well defined with the exception of *C. toxicofera*, which, as mentioned above, has always been broadly defined. For instance, Barneby and Krukoff (1971) treated the three former *Chondrodendron* species [i.e. *Chondrodendron iquitanum* Diels, *C. polyanthum* (Diels) Diels and *C. tomentocarpum* (Rusby) Moldenke] as conspecific with *Curarea toxicofera*. Moreover, the type of *Cocculus toxicoferus* Wedd. is a sterile specimen and, hence, lacks diagnostic features to readily distinguish the species, further complicating the application of this name and circumscription of the taxon, which like most Menispermaceae is also dioecious. To evaluate patterns of morphological variation across the entire range of *Curarea toxicofera*, 200 collections of the taxon were sorted into five groups based on selected features such as relative size of petioles and leaves, lamina shape and indumentum type on its abaxial surface and general features of staminate inflorescence (i.e. slender *vs.* coarser, pedicels and staminate flowers conspicuously larger). These groups of specimens were further evaluated using selected quantitative morphological variables. Based on the outcome of the analyses, three entities are recognised within *C. toxicofera* *s.l.*, namely *C. toxicofera* *s.s.*, *C. iquitana* and *C. tomentocarpa*. Two of the five initial groups within *C. toxicofera*, although distinguished by qualitative features, were however not recovered in the multivariate analyses. Future studies that include other lines of evidence, in addition to morphology, might provide insights about their status and specimens associated with these groups are here tentatively placed under the species to which they are morphologically most similar, preceded by the “*aff.*” qualifier.

The present revision furthers that of Ortiz-Gentry (2000) which sought to evaluate the circumscription of species within the genus *Curarea*, using morphological and anatomical characters. In this study, I provide the taxonomic revision of the species recognised in that work and formally describe the new species treated by Ortiz-Gentry (2000), but were not effectively published there (viz. Melbourne Code Article 30.8). Additionally, in this study, I evaluate the taxonomic boundaries of the *C. toxicofera* complex based on multivariate analyses of the morphological variation across its distribution. Nine species are recognised in *Curarea*, of which two are newly described and two new combinations are proposed. Populations, tentatively identified here as *C. aff. iquitana* or *C.* aff. *Tomentocarpa*, exhibit complex variations that require additional field work in combination with molecular studies to better clarify their taxonomic boundaries. Of the newly described taxa, *C. barnebyana* occurs in Ecuador and Peru, while *C. gentryana* is endemic to western Ecuador and, in a preliminary assessment, is evaluated as endangered.

## Materials and methods

The taxonomic treatment is based upon the examination of 429 herbarium collections from: A, B, BM, BR, ECON, F, G, GH, IAN, INB, K, MG, MO, NY, P, QCNE, R, RB, U, US and USM (acronyms follow Thiers continuously updated). The virtual herbaria of MG (http://marte.museu-goeldi.br/herbario), NY (http://ssciweb.nybg.org/VirtualHerbarium.asp), US (http://collections.nmnh.si.edu/search/botany) and JSTOR Global Plants (http://plants.jstor.org) were also consulted. Additional specimens were collected during field trips to Ecuador and Peru. Label information of all specimens studied are available via TROPICOS, the Missouri Botanical Garden (MO) database, as are the images of all specimens housed at MO. Distribution maps were generated using ArcGIS Desktop Release 10 (ESRI Inc., Redlands, CA), a few specimens whose labels did not indicate coordinates, but had detailed locality information were georeferenced *post facto* using gazetteers, these being found within brackets in the selected specimens examined section under their respective species. A complete list of all specimens examined is presented in the Supplementary material that accompanies this article.

Species boundaries in *Curarea* were inferred based on qualitative and quantitative morphological data (available from the author upon request). Measurements were made from dried herbarium material for leaves, inflorescences and fruits. In *Curarea*, as in all Menispermaceae, development of the fruit is unequal, with the abaxial side developing more than the adaxial side such that the style scar ends up near the adaxial base of the fruit (Fig. 1B). Hence, it should be noted that, although measurements of the fruit length and width seem conventional, i.e. the length referring to the distance between the apex and base of the fruit, the actual apex of the fruit is near the base (Fig. 1B) because of the extreme curvature of the fruit in the Menispermoideae. Similar criteria are followed when measuring endocarps; however, the hippocrepiform embryos are measured from one end to the other, along the outer curvature.

The flowers were rehydrated before measurement. A ruler was used to measure leaves and inflorescences, a digital caliper (Digimatic CD-6” CX, Mitutoyo, Japan) was used to measure fruits and endocarps and a dissecting stereoscope (Nikon, Japan) with a micrometric ocular was used to measure flowers. Inflorescence length includes the short peduncle. The two whorls of sepals in both the staminate and pistillate flowers are always conspicuously different in size and sometimes also in shape, hence measurements of both whorls are reported. However, a single measurement is reported for the two whorls of petals and stamens in the staminate flowers, except when noticeable variation in size and shape is present. For each character, two to three measurements per specimen were taken, the ranges of the averages being used when describing the species.

Most *Curarea* species are morphologically distinct, except for the geographically and ecologically widespread *C. toxicofera* *s.l.* For the latter, I carried out multivariate analyses to evaluate patterns of morphological variation across its entire range. A total of 200 collections were examined and placed in five groups based mostly on variation in general features such as leaf shape and relative size, indumentum type on abaxial leaf surface, the general structure of the staminate inflorescence (i.e. lax or compact), relative staminate flower size and staminate flowers conspicuously pedicellate or subsessile. These five groups, called: **al** = allpahuayo, **iq** = iquitana, **to** = toxicofera, **tm** = tomentocarpa and **uc** = ucayali, below, were further tested by means of a linear discriminant analyses (lda) of selected quantitative data (Table 2) in order to assess the relative importance of the morphological traits in separating the groups (*cf.* Henderson 2005). Of the 200 collections, 73 specimens were staminate, 100 were pistillate and 29 specimens were sterile. To include most of the specimens, only leaves were scored for 197 specimens (Dataset 1), a total of 53 specimens were scored for all characters of leaves + staminate inflorescences + flowers (Dataset 2), 55 for staminate inflorescences + flowers (Dataset 3) and 21 specimens for leaves + fruit characters (Dataset 4). Further, a subset from Dataset 2 was extracted and analysed after removing the toxicofera group, this subset consisted of 27 specimens. All morphological measurements were recorded on an Excel 2010 spreadsheet and log-transformed prior to the analyses. Data analyses were carried out using the package MASS in R ver. 3.1.0 (The R Foundation for Statistical Computing; <http://www.R-project.org>).

**Table 2.** Morphological characters included in multivariate analyses of *Curarea toxicofera* complex.

Organ	Characters (unit)
Leaf	Petiole length (cm)
	Lamina length (cm)
	Lamina width (cm)
Staminate inflorescence	Bract length (mm)
	Main axis length (cm)
	Secondary axis length (cm)
Staminate flowers	Pedicel length (mm)
	Flower length (mm)
	Bracteole length (mm)
	Outer sepal length (mm)
	Outer sepal width (mm)
	Inner sepal length (mm)
	Inner sepal width (mm)
	Outer petal length (mm)
	Outer petal width (mm)
	Inner petal length (mm)
	Inner petal width (mm)
	Outer filament length (mm)
	Outer anther length (mm)
	Inner filament length (mm)
	Inner anther length (mm)
Infructescences and fruits	Infructescence length (cm)
	Infructescence diameter (cm)
	Carpophore length (cm)
	Carpophore diameter (cm)
	Fruit length (cm)
	Fruit width (cm)

A preliminary conservation status assessment for all taxa was carried out according to the IUCN (2012) criteria. Data were analysed using the Geospatial Conservation Assessment Tool, GeoCat (http://geocat.kew.org/) with the default 2 km grid cell.

Features such as wood anatomy, foliar anatomy and leaf venation patterns were also examined. While the few samples per species available for the examination of the characters precluded their inclusion in quantitative analyses, these observations further document interspecific variation in the genus and may provide another line of evidence to distinguish species in *Curarea* when more samples are included.

For wood anatomy studies, stem samples ca. 1.5 cm diameter were available for Curarea aff. iquitana (Diels) R. Ortiz [*Ortiz 181* & *186* (MO)], *C. aff. tomentocarpa* (Rusby) R. Ortiz [*Ortiz & Vargas 197* & *199* (MO)] and of about 5–15 cm wide for *C. tecunarum* [*Ortiz* & *Vásquez 214*, *Ortiz et al. 220* (MO), *van der Werff* & *Vásquez 13909* (MO)], whereas only small twigs of about 1 cm in diameter were available for *C. candicans* [*Lanjouw & Lindeman 2779* (U)], *C. cuatrecasasii* [*Aguilar 682*, *Gentry & Hamilton 41126* (MO)] and for *C. barnebyana* R.Ortiz [*Ortiz & Vargas 200* (MO)]. Permanent slides were prepared following standard techniques in wood anatomy studies. Terms are defined and features are described in accordance with the IAWA committee (1989) guidelines. Table 3 show averages based on 10 measurements of randomly selected cells of the individuals examined.

Sample preparations for foliar anatomy and leaf venation patterns followed Keating (2014). Transverse sections of petiole and mid-lamina, including midrib and lamina margin, were taken. For the petiole, both the apical and basal pulvini as well as the mid-region of the petiole were sectioned. Both fresh and dried leaf samples were used. Fresh field collections were stored in 70% ethanol; dried leaves were first rehydrated in an aqueous solution of Kodak Photo-Flo 200 and stored in 70% ethanol before preparing samples. Observations were made through an Olympus BX40 light microscope at different magnifications. Table 4 summarises the leaf anatomical features and venation patterns observed in *Curarea*.

**Table 3.** Wood anatomy, general features in *Curarea*. * = cambium production unit, *sensu* Jacques & de Franceschi (2007); diam = diameter; rc = ray cells; *crassa*, *gentryana*, *toxicofera* – not studied for wood anatomy.

	*barnebyana*	*candicans*	*tecunarum*	*cuatrecasasii*	*iquitana*	*tomentocarpa*
	Average (range)	Average (range)	Average (range)	Average (range)	Average (range)	Average (range)
Number of samples	1	1	3	2	2	2
pith diameter (mm)	3.2	3	3.4	2.4	2.2	1.2
fiber cap in pith	strong	moderate	strong	weak	weak	weak
Features of xylem bundles & rays in the first CPU*						
# of xylem bundles	39	51	37	25	28	16
xylem bundle shape	nearly terete	nearly terete	nearly terete	nearly terete	terete to wedged	terete to wedged
xylem bundle width adaxial	220(125–275)	160(75–200)	228(150–325)	242(175–350)	194(100–300)	202(125-275)
xylem bundle width abaxial	323(275–400)	233(175–350)	349(275–450)	442(300–575)	259(150–400)	374(175–525)
ray width adaxial	85(50–100)	105(75–125)	81(50–100)	125(75–175)	114(50–125)	12(50–250)
ray width abaxial	150(100–200)	275(225–325)	136(75–250)	210(100–275)	263(150–400)	619(425–1200)
**Vessels**						
# of vessels in xylem bundles in first CPU*	13(9–15)	17(11–25)	21(12–34)	31(22–48)	17(10–30)	32(20-49)
tangential diam. of vessel (µm)	118(50–200)	45(25–75)	107(50–200)	99(50–150)	45(25–75)	68(25-125)
element length (µm)	411(330-410)	359(310-400)	373(300-470)	357(260-470)	277(200–380)	301(100-410)
**Rays**						
height	>1 mm	>1 mm	>1 mm	>1 mm	>1 mm	>1 mm
cellular composition	procumbent & square	procumbent & square	procumbent	procumbent & square	procumbent & square	procumbent & square
**Mineral inclusions**						
prismatic crystals in pith	abundant	moderate	absent	sparse	abundant	abundant
prismatic crystals in ray cells	abundant	abundant	absent	abundant	abundant	moderate to abundant
druses in pith	sparse	sparse	absent	sparse	absent	moderate to abundant
druses in ray cells	absent	absent	absent	absent	absent	absent
stone cells in pith	sparse	sparse	abundant	sparse	absent	absent
stone cells in ray	sparse	absent	absent	sparse	absent	absent
**Other cell inclusions**						
tannins	abundant in rc	sparse in pith & rc	moderate in pith & rc	abundant in pith & rc	absent	sparse in pith cells
tyloses	abundant in pith & rc	abundant in pith & rc	abundant in pith & rc	abundant in pith & rc	abundant in pith & rc	abundant in pith & rc

Cuticle preparations were obtained following methods cited in Christophel et al. (1996). Pieces of leaf of 1 cm^2^ were taken from near the base of the lamina, placed in test tubes and soaked in 70-95% ethanol for about 24 hours. The ethanol was then decanted and 10 drops of 40% hydrogen peroxide and 5 drops of 90% ethanol were added to the tube and then gently boiled in a water bath until the sample turned light yellow. At this point, cuticles were peeled off and cellular material brushed away. Cleaned cuticles were then stained in 0.1% Crystal Violet, wet mounted in CaCl_2_ and observed under the light microscope. Vouchers for leaf anatomy including cuticle and stomata are as follow: *C. barnebyana* [*Ortiz* & *Vargas 194* & *200* (MO)], *C. candicans* [*Jansen-Jacobs et al. 1995* (U)], *Pipoly* & *Boyan 8982* (NY), *C. crassa* [*Jardim et al. 609* (NY)], *C. cuatrecasasii* [*Kernan* & *Phillips 1147* (F), *Callejas 1183* (NY)], *C. gentryana* R.Ortiz [*Rubio* & *Quelal 1503* (MO)], *C. iquitana* [*Vásquez et al. 18715* (MO)], *C. aff. iquitana* [*Ortiz 185* (MO)], *C. tecunarum* [*van der Werff & Vásquez 13909* (MO), *Ortiz et al. 143* (MO)], *C. tomentocarpa* [*Reynel* & *Menezes 5025* (MO)], *C. aff. tomentocarpa* [*Ortiz & Vargas 199* (MO)], *C. toxicofera* [*Encarnación 1094* (MO) and *van der Werff & Vásquez 13990* (MO)].

Scanning electron microscope (SEM) observations of leaf trichomes and stomata were made on the adaxial surface of the samples. Samples of 5 mm^2^ were removed from mid-lamina of apparently adult leaves of herbarium specimens and mounted on SEM stubs with double-sided carbon conductive tabs and coated with gold using a SEM coating unit E5000. Stomatal classification followed Wilkinson (1979) and trichome description and classification followed Theobald et al. (1979). Observations and photographs were made with a Hitachi S-450 SEM @ 20KV using Polaroid panchromatic film.

The difficulty in matching collections of male and female individuals in Menispermaceae has frequently been stressed in the past (Sandwith 1930, Barneby and Krukoff 1971) and the latter authors’ observations regarding the few pistillate flowers in herbarium collections is still relevant at present. In addition to being dioecious, Menispermaceae are also, for the most part, canopy lianas and so difficult to reach, hence they are usually overlooked in biological inventories. It is noteworthy that, although in herbarium collections, there are frequently more pistillate than staminate plants, these pistillate plants are mostly in fruit of different degrees of maturity and therefore are not always readily comparable across species. This is also true for *Curarea*, therefore species are distinguished mostly by characters of the staminate inflorescences and staminate flowers. These latter features are supplemented by characters of the fruits, when available.

In this study, species are regarded as segments of separately evolving lineages of populations, as in the general lineage species concept (de Queiroz 1998, 2007). The criterion used to infer species boundaries is morphological discontinuity, species being diagnosed by unique combinations of qualitative and quantitative morphological features. These morphological features are, as indicated above, mostly based on characters of staminate plants and, for the most part, collections of pistillate plants are assigned to the recognised species if they occur in the same localities where staminate plants have been collected. This approach is rather arbitrary and could be viewed as questionable, especially when the species co-occur as is the case of *C. toxicofera* *s.s.* and the newly resurrected taxa; however, at present doing it in this manner is unavoidable in a dioecious group that is very poorly represented in herbaria collections.

## General morphology

### Habit and stem

**Figure 2.**
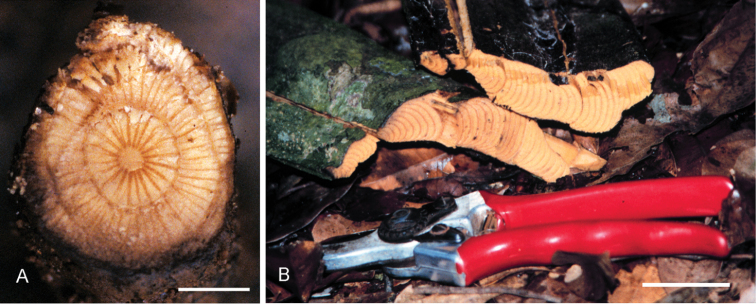
Transversal sections of stem of *Curarea*: **A** relatively round stem of *C. aff. tomentocarpa* (*Ortiz 197*), showing wide rays **B** flattened stem of *C. tecunarum* (*Ortiz & Vásquez 214*) showing successive partial rings. Scale bar: 0.5 cm (**A**); 1.7 cm (**B**).

Species of *Curarea* are all canopy or understory lianas, the canopy species attaining a height of ca. 30 m, the understory species usually only reaching about 10 m high. In understory species, stems are more or less terete (flattened in *C. toxicofera* and ca. 3 cm wide, *Grassl 10076*), with rings or partial rings centric to weakly eccentric, rarely strongly eccentric, in transverse section and 1.5–4 cm in diameter/width (Fig. 2A), while in canopy species, stems are consistently strongly flattened, the partial rings strongly eccentric in transverse section and up to ca. 40 cm in width (Fig. 2B). In both, bark of older stems is usually relatively thin, brittle and smooth, but it can be rugose with irregular shallow lengthwise fissures. Conspicuous tuberculate lenticels are frequent in *C. cuatrecasasii* and in *C. gentryana* and, to some extent, in *C. iquitana*.

### Wood anatomy

Wood anatomy of *C. crassa*, *C. gentryana* and *C. toxicofera* *s.s.* was not investigated. For the remaining species, mostly one sample per species was examined. Transverse sections of the stem of *Curarea*, as of several other Menispermaceae, show an irregular pattern of secondary growth consisting of successive, complete or partial rings of vascular bundles, which are separated radially by wide interfascicular rays and tangentially by pericyclic fibres and tangential cortical parenchyma. This type of growth is typical of Menispermaceae and has been termed “anomalous secondary growth” (Solereder 1908); “included (interxylary) phloem” (Chalk and Chataway 1937; Metcalfe and Chalk 1950), “xylem and phloem concentrically alternating” (Metcalfe and Chalk 1983) or “successive cambia” (Carlquist 1988, 1996; Jacques and de Franceschi 2007).

Within *Curarea*, species differ moderately in their pith diameter, distribution patterns of vascular bundles and rays, occurrence of medullary sclerenchyma (e.g. fibre caps) and number and mean tangential diameter of vessels. Variation in these and other features are summarised in Table 3.

**Figure 3.**
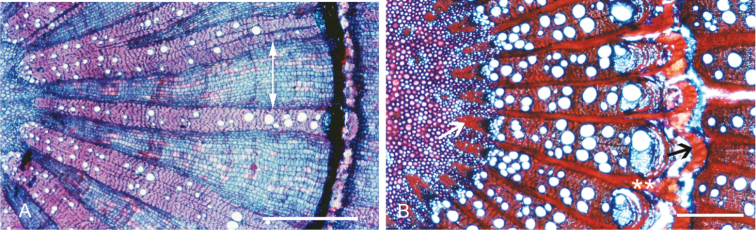
LM photographs of transverse sections of wood of *Curarea*: **A** small vessels, and broad rays (double arrow), *C. aff. tomentocarpa* (*Ortiz & Vargas 197*) **B** moderately large vessels, sclerotic pericyclic fibres (black arrow), strongly developed medullary sclerenchyma (white arrow) and sclerenchyma strongly intruding into the rays (two white asterisks), *C. tecunarum* (*van der Werff & Vásquez 13909*). Scale bar: 0.8 mm (**A, B**).

Successive and centric to weakly eccentric patterns of few (3–7) complete or partial rings are found in *C. aff. tomentocarpa* (Fig. 2A) and *C. cuatrecasasii* and several (ca. 45) strongly eccentric arches are observed in *C. tecunarum* (Fig. 2B), *C. candicans* and *C. barnebyana*. Wide rays (275–340 μm) in combination with more or less straight tangential margins and vascular bundles with smaller vessel diameter are observed in *C. aff. tomentocarpa* (Fig. 3A), *C. candicans* and, to some extent, in *C. cuatrecasasii*. There are tangential wedge-like layers of sclereids intruding into the rays in *C. tecunarum* (Fig. 3B) and *C. barnebyana* but, in all other species examined, the rays are tangentially uniform. Relatively narrow rays (150–136 μm) with conspicuous irregularly angulate radial margins combined with relatively wide (220–228 μm) vascular bundles with large tangential diameter of vessels and strongly developed medullary sclerenchyma in the pith are characteristic of *C. tecunarum* (Fig. 3B) – this alone lacks crystalline inclusions– and *C. barnebyana*.

Relatively narrow rays with conspicuous irregularly angulate radial margins in combination with vascular bundles with large tangential diameters are also observed in *C. cuatrecasasii*. No representative of *C. toxicofera* *s.s.* was available in this study and sterile specimens provisionally identified as *C. toxicofera* show moderate to strongly eccentric arches of successive growth, although the width reaches only up to 3 cm.

### Leaves

**Figure 4.**
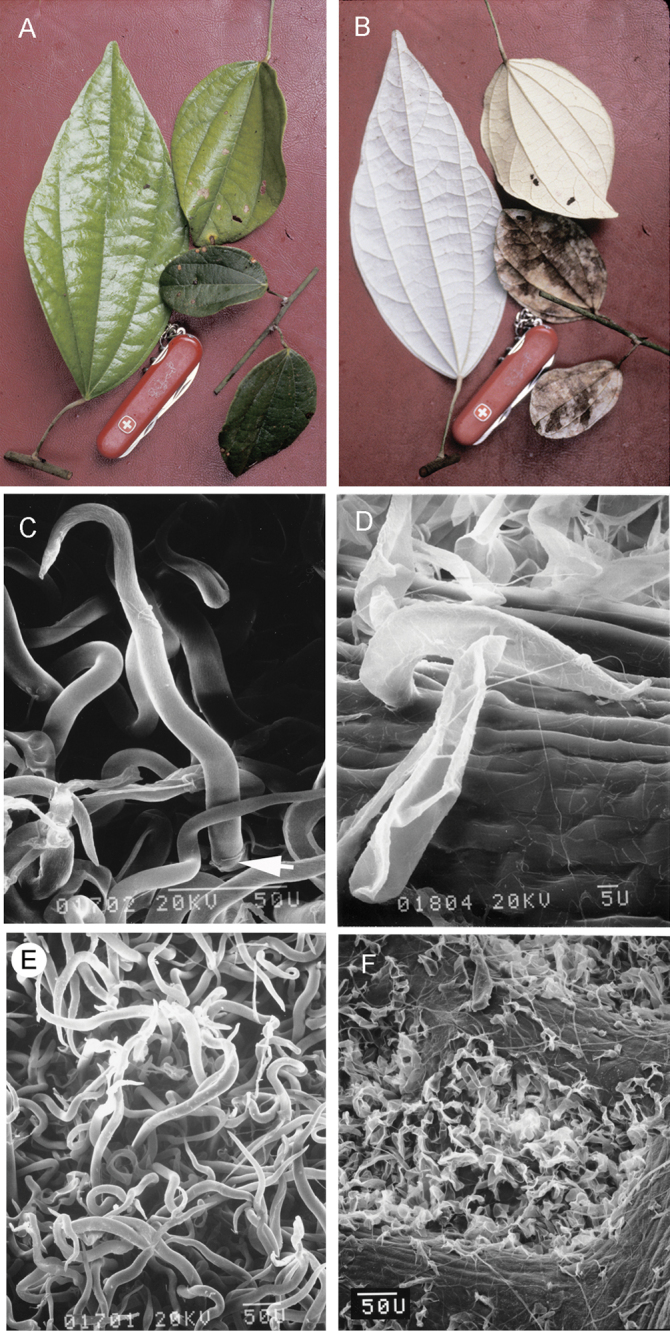
Leaf blade surfaces and SEM micrographs of trichomes of *Curarea*: **A** glabrous adaxial leaf blade surface of juvenile and adult leaves, *C. barnebyana* (*Ortiz & Vargas 194*) **B** densely pubescent adaxial leaf blade surface of juvenile and adult leaves showing whitish covering more conspicuously in juvenile leaves, *C. barnebyana* (*Ortiz & Vargas 194*) **C** elongate trichome with a short basal cell (white arrow) and a cylindrical terminal cell, *C. crassa* (*Jardim et al. 609*) **D** trichome with a moderately short terminal cell, *C. cuatrecasasii* (*Kernan & Phillips 1147*) **E** matted, elongate trichomes, *C. gentryana* (*Rubio & Quelal 1503*) **F** collapsed trichomes, *C. candicans* (*Jansen-Jacobs et al. 1995*).

Within an individual, the shape and size of the lamina varies with age, position along the stem and exposure to sunlight. Thus, large blades with caudate apices are usually either juvenile and/or found in parts of the plant that are in shaded areas, while smaller blades with acute or acuminate apices are found in mature leaves that are usually exposed to direct sunlight. The latter are more conspicuous in species that reach the canopy. The type collection of *C. tecunarum* –i.e., *Krukoff 8713* and a sterile specimen of *Curarea* (*Cerón 2717*), which remains as *Curarea* sp., show a few leaves with cleft apices. The margins of blades are entire, but on rare occasions, they are scarcely lobed to apically trilobed in sterile specimens of *C. tecunarum* (*Vásquez et al. 15101*, *Lewis et al. 11759*) and the sterile specimen *L.C. Richard s.n.*, the type of *C. candicans*, also has an apically trilobed leaf blade. Leaf blades are chartaceous or subcoriaceous, sometimes thinly fleshy when young, surfaces are usually conspicuously discolorous (Fig. 4A–B) resulting from the dense silvery indumentum covering the adaxial surface (Fig. 4B), especially in juvenile leaves. Petioles are shorter in leaves of the canopy and longer in leaves of the understory and/or shaded areas, pulvinate at both ends, with the apical one more conspicuous.

### Indumentum

The indumentum in *Curarea* consists of two-celled unbranched trichomes with a short basal cell and an elongate terminal cell (Fig. 4C–D). Trichomes occur singly or in pairs.

Several-lobed trichomes have been reported for *C. toxicofera* by Wilkinson (1989), but could not be confirmed here. It is likely that the tendency for the terminal cells to collapse in an irregular fashion in dried and/or aged samples gives the impression of trichomes being lobed. Indumentum density varies with age in some species, frequently disappearing along the stems. This variation is of taxonomic use in *Curarea*. Thus, uniformly dense indumentum concealing the lower leaf surface is common in *C. crassa*,

*C. barnebyana* and *C. tecunarum* (Fig. 4E), whereas the indumentum mainly localised in the areole spaces, is common in *C. candicans* (Fig. 4F), *C. cuatrecasasii* and less frequently also in *C. toxicofera* *s.s.*

### Venation pattern

**Figure 5.**
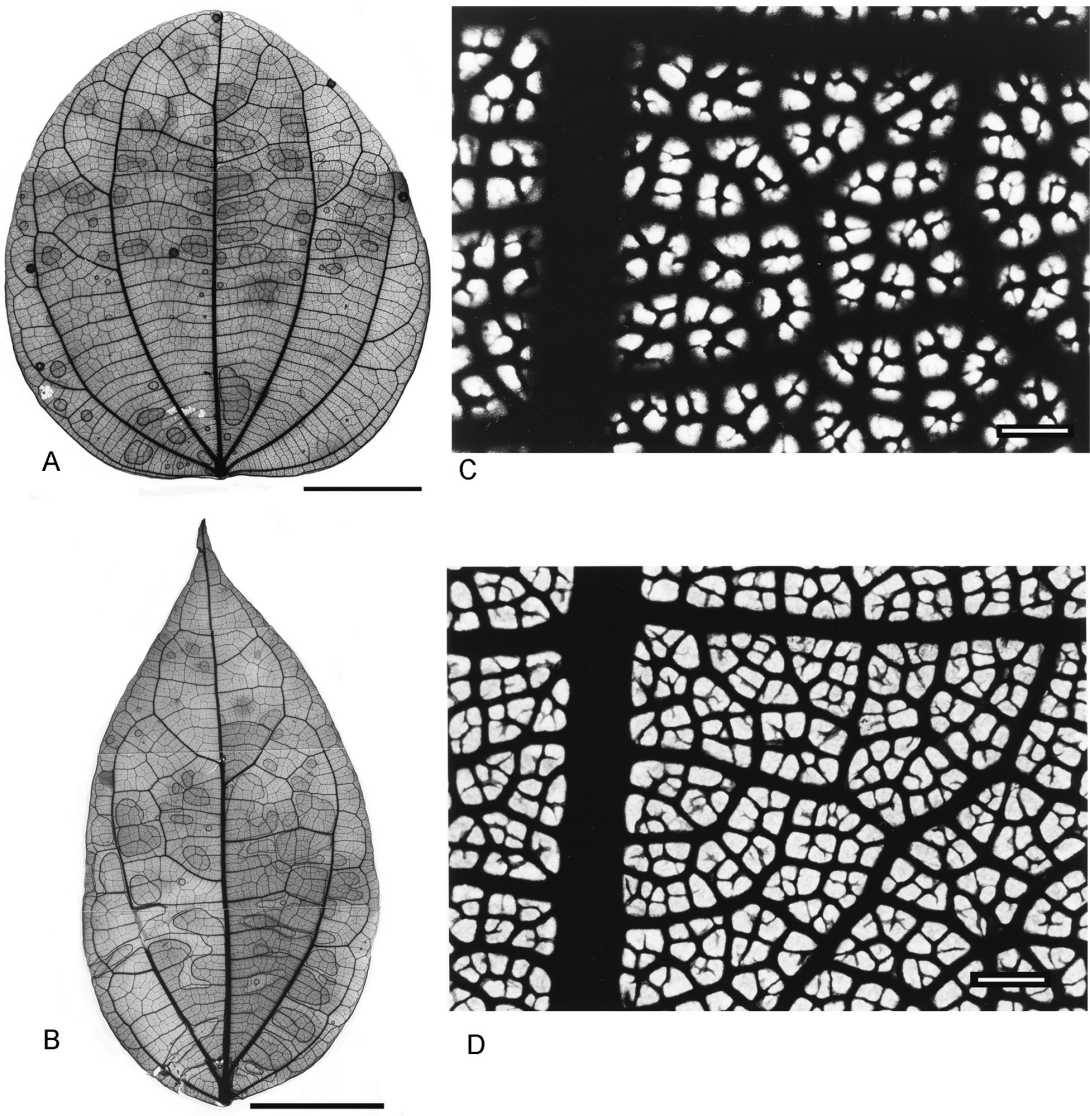
Venation types of *Curarea*: **A** innermost pair of main veins acrodromous perfect, 7 main veins from the base, the basal-most pair weakest, *C. tecunarum* (*van de Werff & Vásquez 13909*) **B** innermost pair acrodromous imperfect, 3 conspicuous main veins from the base, *C. toxicofera* (*van der Werff & Vásquez 13990*) **C** well-developed areolae and ultimate veinlets often thin, free vein endings uncommon, *C. candicans* (*Jansen-Jacobs et al. 1995*) **D** well-developed areolae and ultimate veinlets thin, once or twice branched, endings often free *C. cuatrecasasii* (*Morales et al. 3243*). Scale bar: 3.1 cm (**A**); 0.7 mm (**B**); 3.0 cm (**C**); 1.1 mm (**D**).

The venation in *Curarea* conforms to the acrodromous type (*sensu* Hickey 1973, 1979) as observed in mature leaves. Primary veins vary from 3–5(–7), (Fig. 5A–B), however in one group of species (i.e. *C. candicans*, *C. crassa*, *C. barnebyana* and *C. tecunarum*, Fig. 5B), the innermost pair runs almost the whole length of the lamina and arches towards the apex, with only one pair of loosely brochidodromous secondary veins (this pair sometimes absent) and thick and coarse veinlets (higher-order veins), (Fig. 5C). In the other group of species which includes *C. cuatrecasasii*, *C. gentryana*, *C. iquitana*, *C. tomentocarpa* and *C. toxicofera*, although the innermost pair runs more than half the length of the lamina, frequently there are 2–3 pairs of strongly brochidodromous secondary veins and moderately thin and loose veinlets (Fig. 5B, D).

### Leaf anatomy: adaxial cuticle, transverse sections of petiole, lamina and mid-vein

Most of these features were studied by Wilkinson (1989) for *C. candicans* and *C. toxicofera* *s.l.* when describing characters common to Tiliacoreae as a whole.

**Figure 6.**
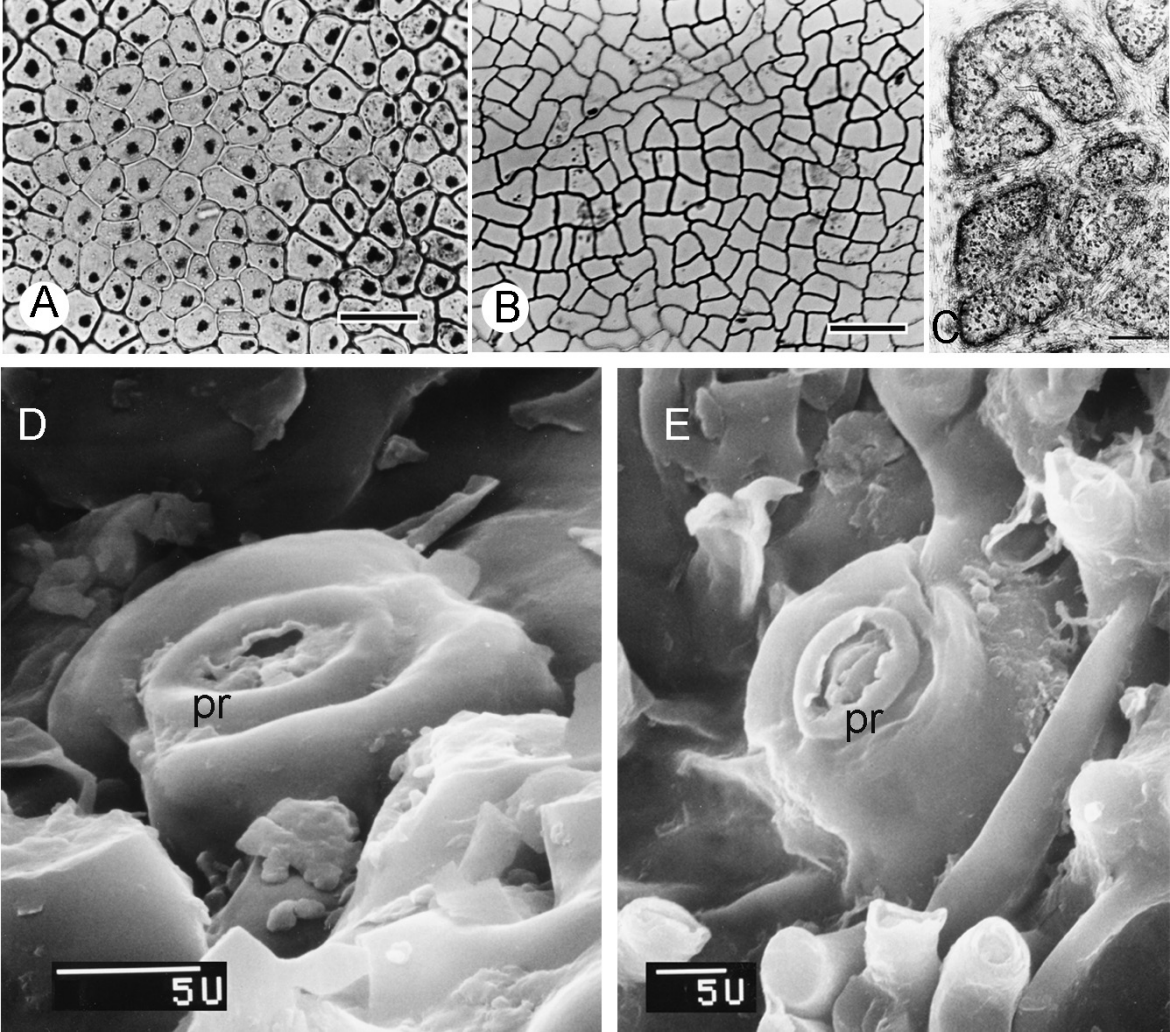
LM photograph and SEM micrographs of leaf adaxial, abaxial surface and stomata in *Curarea*: **A** isodiametric epidermal cells, *C. candicans* (*Jansen-Jacobs et al. nr-1995*) **B** triangular to hexagonal epidermal cells, *C. cuatrecasasii* (*Kernan & Phillips* 1147) **C** abaxial surface showing areoles (black dotted) in *C. toxicofera* (*van der Werff & Vásquez 13990*) **D, E** SEM micrographs of projecting stomata with distinct peristomatal rim **D** *C. tecunarum* (*Ortiz et al. 143*) **E** *C. tomentocarpa* (*Reynel & Meneses 5025*). Scale bar: 50 µm (**A**); 100 µm (**B**), 0.4 mm (**C**), **pr** = peristomatal rim.

Here, I add observations of the remaining species, the main variations being summarised in Table 4. Adaxial epidermal cells in all species are squarish, rectangular or irregularly shaped. In *C. candicans* (Fig. 6A), *C. crassa*, *C. barnebyana* and *C. tecunarum* these have abundant tanniferous cells, but there is little or no tanniferous cells in *C. cuatrecasasii* (Fig. 6B), *C. gentryana*, *C. iquitana*, *C. tomentocarpa* and *C. toxicofera*.

*Curarea* species are exclusively hypostomatic, as noted by Wilkinson (1989). The stomata, between the strongly projecting veins (Fig. 6C), are concealed by a dense indumentum. They occur typically in groups of 7–102 per areole, with the lowest number observed in *C. tomentocarpa* and the highest in *C. barnebyana* (Table 4). Stomata are elliptic in outline and immediately surrounding the aperture is a distinct peristomatal rim (Fig. 6D–E), the cell guards range in size from 26–36 × 23–35 μm. In all species, stomata are raised on a short column of ca. 4 cells (5–6 cells *fide* Wilkinson 1989).

**Table 4.** Leaf anatomy general features in *Curarea*. av = average; diam = diameter; TS = transverse section; vb = vascular bundles.

	*barnebyana*	*candicans*	*crassa*	*tecunarum*	*cuatrecasasii*	*gentryana*	*iquitana*	*tomentocarpa*	*toxicofera*
Number of samples	2	2	1	2	3	1	3	2	2
average diam. of apical pulvinus (mm)	2	2.5	ca. 1	2.8	1.8	1.6	3	1.9	2.3
number of vb. in apical pulvinus	11–12	10	9	11–12	8–9	12	10–13	9–11	11–12
av. diam. in petiole mid-region (mm)	1.5	1.3	0.9	1.8	1.2	1.1	2.1	1.4	1.6
av. blade thickness (µm)	94	116	ca. 110	86	82	ca. 79	74	80	85
av. cuticle + adaxial periclinal	23	24	ca. 21.5	19	14	ca. 16	17	17	19
cell wall size (µm)									
adaxial epidermis cell shape	squarish, rectangular or irregular	squarish or rectangular	squarish or irregular	squarish or irregular	squarish, rectangular or irregular	squarish, rectangular or irregular	squarish, rectangular or irregular	squarish, rectangular or irregular	squarish or rectangular
tannins	abundant	moderate	moderate	moderate	scarce or absent	scarce or absent	scarce or absent	scarce or absent	absent to moderate
range of stomata number/crypt	17–102	15–32	15–46	36–34	16–27	10–17	12–34	7–30	27–37
mean stomata guard cell size (µm)	ca. 26 × 23	36 × 35	30 × 30	27 × 31	ca. 27 × 27	33 × 31	31 × 33	31 × 31	28 × 28
mesophyll palisade cell arrangement	rather compact	rather compact	rather compact	rather compact	loose	loose	rather compact	rather compact	rather compact
outer mesophyll palisade cell size (µm)	26 × 4	23 × 5	24 × 2.8	23 × 5	17 × 5	18 × 7	16 × 6	14 × 5	19 × 5
shape of mid-vein in TS:									
adaxially	concave	flattened or weakly concave	weakly concave	concave	flattened or weakly convex	flattened or weakly convex	weakly convex	flattened	flattened or weakly convex
abaxially	triangular or trapezoid	triangular or trapezoid	triangular or trapezoid	triangular, trapezoid or suborbicular	transversally ellipsoid	triangular	triangular or trapezoid	triangular or trapezoid	rectangular or transversally ellipsoid
trichomes on mid-vein abaxially	abundant	a few basal cells or glabrate	sparse to abundant	abundant	sparse to absent	sparse basal cells to glabrate	sparse basal cells to glabrate	sparse basal cells to glabrate	sparse basal cells to glabrate
number of vb. in mid-vein	3–6	3	6	3–5	3–5	3	3–7	4–6	5–9
number of vb. in petiole main axis	10–12	8–10	10–12	11–12	8–9	12	10–14	9–11	11–12
venation type of acrodromous	perfect	perfect	perfect	perfect	imperfect	imperfect	imperfect	imperfect	imperfect
veinlets	relatively thick	relatively thick	relatively thick	relatively thick	relatively thin	relatively thin	relatively thin	relatively thin	relatively thin

Wilkinson (1989) described this arrangement of the epidermal cells under the guard cells as cyclocytic. Such projecting stomata with thick peristomatal rims are frequently associated with ecological factors such as high humidity and poor soils (Solereder 1908; Wilkinson 1979), conditions typical of the habitats occupied by species of *Curarea*.

**Figure 7.**
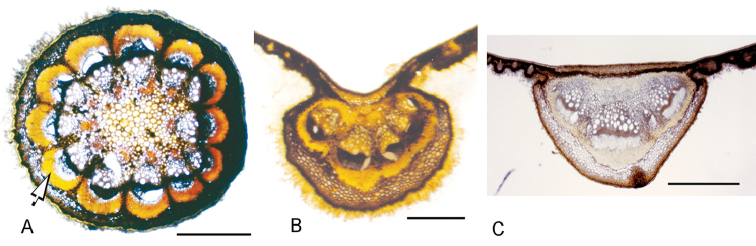
LM photographs of transverse sections of petiole and mid-veins of *Curarea*: **A** petiole mid-section of *C. tecunarum*, note the vascular bundles surrounded by sclerotic fibres on the outer part (arrow) **B** adaxially concave, abaxially strongly raised, rounded and tomentose mid-vein, *C. tecunarum* (A, B from *Ortiz et al. 143*); **C** adaxially flat, abaxially strongly raised, triangular and glabrescent mid-vein, *C. toxicofera* (*van der Werff & Vásquez 13990*). Scale bar: 0.6 mm (**A, C**), 1 mm (**B**).

The apical pulvinus of the petiole is more or less circular to weakly triangular in transverse section and weakly adaxially flattened in *C. candicans*, *C. crassa* and *C. tecunarum*. There are no trichomes or only a few persistent basal cells in *C. candicans*, *C. cuatrecasasii*, *C. gentryana*, *C. iquitana*, *C. tomentocarpa* and *C. toxicofera*; usually there are abundant trichomes in the remaining three species. The vascular tissue is a ring of 8–13 vascular bundles. Sclerenchyma cells surround the vascular tissue, externally forming an interrupted or continuous ring of several layers. There are a few small groupings of sclereids (stone cells) in the inner parenchyma of the cortex and, in the pith, a starch sheath (i.e. endodermis) is found around the vascular tissue in all species. A pulvinus at both ends of the petiole is a common feature in Menispermaceae and they are thought to bring the lamina into the optimum position to receive light (Wilkinson 1989). Pulvini have a large volume of parenchyma; therefore, they are always swollen compared to the rest of the petiole (Esau 1967). The mid-region of the petiole is circular, from about 1 mm diameter in *C. crassa* and *C. cuatrecasasii*, to about 2.1 mm in diameter in *C. iquitana*. Trichomes are few to rather abundant, frequently only the basal cells are present. There are 8–14 vascular bundles surrounded by a continuous ring of thick-walled sclerenchyma cells, the layers of parenchyma cells are much reduced and frequently thick-celled parenchyma is found in the pith (Fig. 7A).

Transverse section of the lamina shows mesophyll palisade cells that are somewhat short and loosely arranged especially the inner layer in *C. cuatrecasasii*, *C. iquitana*, *C. gentryana*, *C. tomentocarpa* and *C. toxicofera*. Palisade cells are moderately long and more or less compactly arranged in *C. barnebyana*, *C. candicans*, *C. crassa* and *C. tecunarum*.

The midrib is adaxially concave and abaxially strongly raised and trapezoid or sub orbicular but always with abundant trichomes in *C. barnebyana*, *C. crassa* and *C. tecunarum* (Fig. 7B). In *C. cuatrecasasii*, *C. gentryana*, *C. iquitana*, *C. tomentocarpa* and *C. toxicofera*, the midrib is adaxially flat to weakly convex and abaxially strongly raised, triangular or trapeziform with few or no trichomes (Fig. 7C). The midrib in *C. candicans* adaxially is somewhat flat to weakly concave while abaxially, it is strongly raised and somewhat trapeziform with sparse trichomes or glabrate. As noted by

Wilkinson (1989), the vascular system in *Curarea* is frequently collateral. There are 3–9 vascular bundles distributed in an arc (Fig. 7B–C), the three central ones being largest. The vascular bundles are surrounded by several layers of sclerenchymatous cells. Adaxial to the vascular bundles there is a continuous (e.g. *C. barnebyana*, *C. candicans*, *C. crassa* and *C. tecunarum*) or sometimes interrupted (e.g. *C. cuatrecasasii*, *C. gentryana*, *C. iquitana*, *C. tomentocarpa* and *C. toxicofera*) layer of chlorenchyma cells and one or several layers of sclerenchymatous cells immediately below the epidermis.

Abaxially, parenchyma cells are abundant, with few to numerous stone cells scattered throughout and below the abaxial epidermis are sclerenchymatous cells.

### Inflorescences

As all Menispermaceae, *Curarea* is also dioecious, with staminate and pistillate inflorescences in different plants. Staminate and pistillate inflorescences are solitary or more frequently fascicled, cauliflorous and arising from old leafless stems, axillary, supra axillary or terminal on young shoots. Inflorescences are basically thyrsi (*sensu* Weberling 1992), however, in pistillate inflorescences, the primary branches are sometimes reduced to solitary flowers, thus appearing racemiform; inflorescence axes are slender or moderately stout, sometimes conspicuously ridged, variously densely pubescent, with trichomes adpressed or ascending. The inflorescence bracts subtending the primary branches are ovate to lanceolate, markedly concave and usually fleshy, the adaxial side glabrous, the abaxial side variously pubescent, the indumentum appressed or spreading.

**Figure 8.**
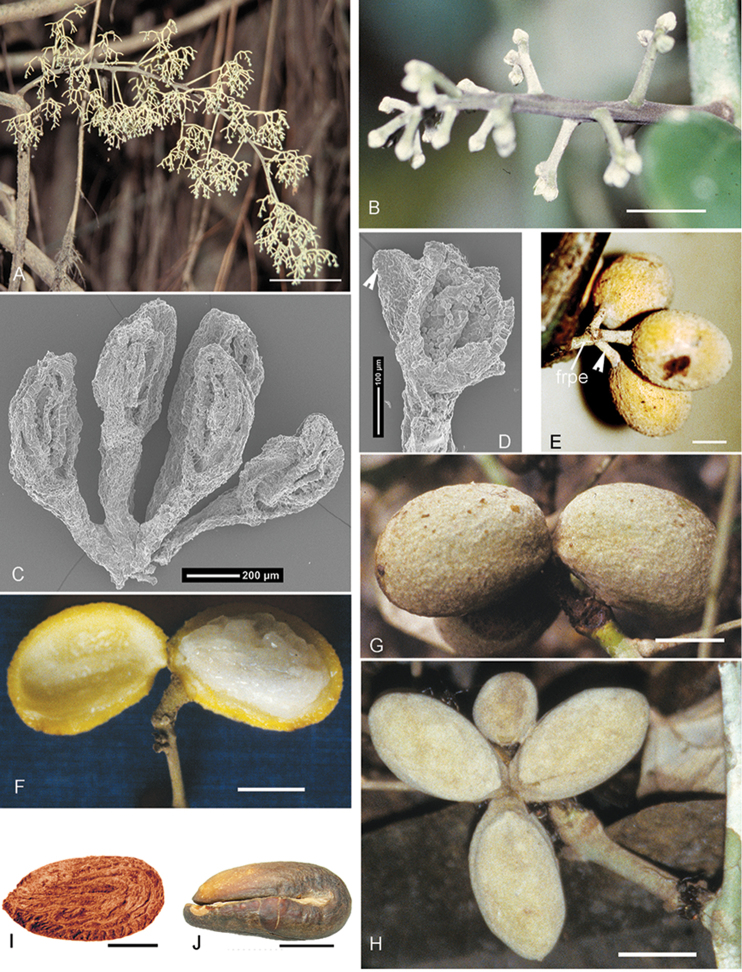
General morphology in *Curarea*: **A.** staminate inflorescence of *C. toxicofera* (*Ortiz et al. 157*) **B** pistillate inflorescence of *C. aff. iquitana* (*Ortiz 184*) **C** stamens of *C. toxicofera* (*Encarnación 1094*) **D** anther of *C. iquitana* showing the horn-like (arrow) adaxial protrusion of the connective (*Vásquez et al. 18994*) **E** ripe fruits of *C. aff. iquitana* showing the fruiting pedicel (frpe) and three elongate carpophores (white arrow) (*Ortiz & Ruiz 191*) **F** ripe fruit of *C. aff. iquitana*, exocarp of drupelet on right pulled away showing whitish mesocarp (*Ortiz & Pezo 208*) **G–H** immature fruits of *C. barnebyana* **G** lateral view **H** view from the top (**G–H** from *Ortiz & Vargas 194*) **I** weakly ornamented endocarp of *C. toxicofera* (*Lewis & Vásquez 4017*) **J** hippocrepiform embryo of *C. barnebyana* (*Ortiz & Vargas 194*). Scale bars: 5.2 cm (**A**), 1.1 cm (**B**), 1 cm (**E**), 1.25 cm (**F**), 1.5 cm (**G–H**), 0.5 cm (**I**), 1 cm (**J**).

*Staminate inflorescences* usually multiflorous (Fig. 8A); lax primary branches with (2–)4–6 branching orders are characteristic of *C. cuatrecasasii*, *C. gentryana*, *C. iquitana*, *C. tomentocarpa*, *C. toxicofera* (Fig. 8A) and *C. tecunarum*. Rather condensed/compacted primary branches with 0–2 branching orders appearing umbelliform or irregularly cymose occur in *C. crassa*, *C. barnebyana* and *C. candicans*; occasionally, *C. candicans* may have 3 orders of branching. In *C. tecunarum*, higher branching orders frequently are reduced (i.e. they are not fully dichotomous and flowers are sessile or subsessile), occasionally in *C. gentryana* and *C. aff. iquitana*, reduced branching occuring on alternating sides along the length of the primary branches, appearing racemiform. In most species, the primary branches are moderately thick, but they are rather thin and filiform in *C. cuatrecasasii* and *C. gentryana*.

*Pistillate inflorescences* are usually pauciflorous (Fig. 8B), with the primary branches mostly simple dichasia subtended by an inflorescence bract. Some or all of the primary branches may be reduced to single flowers, thus resembling a raceme and these flowers are subtended by bracts. In branched inflorescences of both staminate and pistillate there may no be obvious floral bracts.

### Flowers

Flowers are unisexual, actinomorphic and trimerous; usually pedicellate, less frequently sessile. The pedicels are usually terete, moderately slender and sometimes ridged. At the apex of the pedicels there are 1–3(4) minute, more or less deciduous structures, usually alternating with the outer whorl of sepals. Although their morphological nature is often unclear, to facilitate description, here I am referring to them as bracteoles. These structures are most commonly ovate-lanceolate, narrowly ovate to ovate or oblong, fleshy, glabrous adaxially, variously pubescent abaxially. They are most often absent in staminate flowers and, hence, frequently not mentioned in earlier descriptions (i.e. Barneby and Krukoff 1971).

*Staminate* flowers are usually light coloured, frequently described as cream, whitish, greenish, yellowish, orangish, greyish or brownish; although flowers are usually pedicellate, they can be occasionally sessile in *C. toxicofera* and *C. barnebyana* and more often so in *C. tecunarum*. Sepals are commonly 6(–9), free, in 2(–3) unequal whorls (sometimes spirally arranged in *C. candicans*), mostly ovate, narrowly ovate to obovate, oblong, rhombic, elliptic or suborbicular, usually weakly concave and scarcely fleshy, glabrous adaxially, variously pubescent abaxially, the outermost whorl being smaller than the innermost whorl. The inner sepals have valvate aestivation and, after anthesis, their tips are usually reflexed (rarely erect). Petals are (5–)6(–9 in *C. tomentocarpa*), free, arranged in 2(–3) usually similar whorls, they are conspicuously smaller than the inner sepals, usually obovate-trilobed to rhombic or spatulate members, weakly concave, membranous, glabrous adaxially, glabrous or tomentellous abaxially, the lateral margins strongly inflexed and clasping the filaments of the stamens immediately opposite to them. In *C. gentryana*, the lateral margins of the inner whorl are adaxially connate or coherent. The stamens are (3–5)6, (Fig. 8C), arranged in two, more or less similar whorls (often one in *C. gentryana*); the filaments are free or variously connate (connate half their length in *C. aff. iquitana* to fully connate in *C. gentryana*) and clavate, clavate-sigmoid to almost terete, glabrous (abaxially tomentellous in *C. toxicofera*). The anthers are tetrasporangiate and dithecous, basifixed, ellipsoid or subglobose in shape, erect or slightly incurved and dehiscing by latrorse longitudinal slits. They are yellow in colour when fresh and light brown or cream when dried. Frequently, the sporangia appear half-immersed in the dilated connective. In all species, the connective is conspicuous on both sides, however, it is frequently thicker adaxially and variously protruding and it sometimes grows beyond the thecae to form a horn-like structure, as in *C. iquitana* (Fig. 8D). The connective is sometimes thinner abaxially and, at the apex in *C. tomentocarpa* and here, each theca may separate at the apex. Pistillode absent.

*Pistillate* flowers are greenish, yellowish or brownish coloured; pubescent abaxially. Sepals 6–9, free, in two, or more commonly three unequal whorls, mostly ovate-lanceolate to obovate, oblong, elliptic to rhombic or spatulate, usually weakly concave and scarcely fleshy to fleshy, glabrous adaxially, variously pubescent abaxially, the outermost whorl being smaller than the innermost whorl. The inner sepals have valvate aestivation and, after anthesis, their tips are usually reflexed (erect). Petals are usually in a single whorl of three, less frequently 4–6 petals are found in *Curarea aff. iquitana*, *C. tecunarum* and *C. tomentocarpa*, they are spatulate and weakly concave, membranous, glabrous adaxially, glabrous to scarcely pubescent abaxially. The petals are always smaller than the innermost whorl of sepals and are usually opposite the carpels. There are three (seldom four in *C. tomentocarpa*), free carpels, the ovaries are usually coherent when young, strongly gibbous and densely tomentose; the styles are glabrous and usually terete, weakly tapering distally, often reflexed, weakly grooved adaxially in transverse section and commonly subpersistent and located near the base of the fruit; the stigma is inconspicuous. Frequently only one carpel reaches maturity.

### Infructescences and drupelets

*Infructescences* are similar to inflorescences, but are sometimes lenticellate or have exfoliating bark. Fruiting pedicels are clavate or terete, at times inconspicuous, slender or rather thick. As the carpel develops in *C. iquitana*, *C. tomentocarpa*, *C. toxicofera*, *C. cuatrecasasii* and *C. gentryana* (i.e. all understory species in which the fruits are close to the ground), the trilobed gynophore enlarges into three relatively short to moderately long stalks (Fig. 8E). This enlargement of the gynophore produces structures known as carpophores, which support the drupelets of each carpel (Forman 1975, 1986). They are weakly terete or clavate, straight or distally weakly incurved and reach 2.7–11.3 mm in length, the longest being observed in *C. toxicofera*. Carpophores are also found in other genera in the tribe Tiliacoreae (i.e. the neotropical *Sciadotenia* and some African and Indomalesian species of *Tiliacora*; Forman 1975), as well as other genera outside Tiliacoreae (viz. *Elephantomene* of Anomospermeae, see Krukoff and Barneby 1974; and *Anamirta* of Coscinieae, see Forman 1975). However, in *C. barnebyana*, *C. crassa* and *C. tecunarum*, this enlargement does not occur and only a subglobose, weakly trilobed structure is observed at the distal end of the peduncle and, in *C. candicans*, this is somewhat drum-like; these four species bear fruits in the canopy.

*Drupelets* are oblongoid, ellipsoid, obovate or subglobose (Fig. 8E–H) and are weakly laterally compressed when dried. They are obliquely attached to the summit of the carpophore, sessile or less frequently gradually attenuate toward the base, forming a short stipe ca. 3 mm long in some specimens of *C. cuatrecasasii*. The exocarp may be thin or thick, mealy-coriaceous in *C. toxicofera* (*sensu* Barneby and Krukoff 1971), the surface is smooth, rugulose or muriculate, pilosulous or velutinous-hispidulous, the trichomes being erect. The fruits are pale orange to yellow when ripe and frequently leathery outside and granular inside when dried. Although, in a strict sense, the single-layered outer epidermis of the pericarp is referred to as the exocarp, here I am following earlier characterisations of *Curarea* fruit by Barneby and Krukoff (1971), who described the several-layered outermost zone of the pericarp (including the epidermis) as the exocarp. It is worth noting, however, that it appears that Barneby (1996) interpreted the exocarp as the epidermis only and the remaining layers following the epidermis as the mesocarp when describing the fruit in *C. crassa* from dried herbarium material (e.g. Barneby 1996: 22). The mesocarp is white, fleshy and mucilaginous when fresh (Fig. 8F) and, when dried, turns into filamentous plates that adhere to the endocarp (Barneby and Krukoff 1971). Due to the eccentric development of the carpel, the scar of the style is found near the base of the developed fruit. The endocarp is oblong-ellipsoid or obovoid, hippocrepiform (i.e. horseshoe-shaped) (Fig. 8I), a shape produced by the unequal development of the abaxial side compared to the adaxial side (Botha 1980; Forman 1986); although the hippocrepiform shape is more noticeable from within rather than from the external surface. The texture is typically papyraceous (crustaceous in some specimens of *C. toxicofera*). Commonly the surface is fairly smooth, but muriculate or weakly reticulate in *C. toxicofera* (Fig. 8I). Seeds, like the endocarps, are hippocrepiform (Fig. 8J), semicircular in transverse section and brownish when dried. Seeds in *Curarea*, as in most members in the tribe Tiliacoreae, lack endosperm, the cotyledons are thick, fleshy and accumbent, both are usually of equal size, but in *C. barnebyana* one of them may be smaller and somewhat J-shaped. The seed (embryo) is hippocrepiform (i.e. curved around the condyle). The latter has not been studied in developing carpels in *Curarea*, however, features of the mature endocarp suggest that it conforms to a bilaterally compressed septiform condyle as described by Ortiz (2012); the condyle corresponds with a shallow groove on the lateral sides of the external surface of the endocarp.

### Pollen morphology

Pollen morphology of *Curarea* represented by *C. toxicofera* *s.l.* was examined by Thanikaimoni (1968), Ferguson (1975) and Thanikaimoni et al. (1984); the latter authors included also *C. tecunarum*. Pollen is described as spheroidal, tricolporate, with circular equatorial outline, muri distinctly papillose, papillae in one or two rows.

### Phytochemistry

Several members of Menispermaceae are known to contain toxic compounds and bisbenzyltetrahydroisoquinoline alkaloids are very common in the family (Bruneton 1995, Barbosa-Filho et al. 2000). Amongst the best known is (+)–tubocurarine, a muscle relaxant produced by *Chondrodendron tomentosum*, a close relative of *Curarea* and the basis for subsequently developed synthetic forms (Bruneton 1995, Tuba et al. 2002). *Curarea toxicofera* produce alkaloids with curare-like activities such as (-)-curine and (+)-chondrocurine, (+)-isochondrodendrine; the latter has also been reported in *C. tecunarum* (Barbosa-Filho et al. 2000).

### Ethnobotany

Menispermaceae are rich in medicinal and toxic compounds and many species are used to cure a variety of illnesses across their global distribution. Amongst the best-known products is curare, the South American arrow and dart poison of which an overall introduction is given by Krukoff and Moldenke (1938). Curare is a general name for a large group of poisons with muscle paralysis activities (Neuvinger 1998) and several species of *Curarea* are a source of this type of poison. *Curarea candicans* [= *Chondrodendron candicans* (Rich. *ex* DC.) Sandwith)] was reported by Sandwith (1930), based on the label information of *Jenman 5199* to be used by the Warrou Indians of Guyana as a source of the “other kind of poison”, a statement that was interpreted by Krukoff and Moldenke (1938) to refer to a different substance from the curare used by the Macusi Indians, also from Guyana.

*Curarea tecunarum*, variously identified as *Chondrodendron polyanthum* (Diels) Diels or as *Chondrodendron limaciifolium* (Diels) Moldenke, was reported as one of the main sources of curare for the Tecuna Indians from Brazil (Krukoff and Smith 1937; Krukoff and Moldenke 1938; Krukoff and Barneby 1970). The early report of Krukoff and Smith (1937) was later mentioned by Macbride (1938) in the Flora of Peru, under the name of *Chondrodendron polyanthum*. An extract from *C. tecunarum* is used as a long-term oral contraceptive for both men and women of the Denís tribe of Brazil (Barneby and Krukoff 1971; Prance 1972).

*Curarea toxicofera* (as *Cocculus toxicoferus* Wedd.) has long been known as an important source of curare for the Yaguas and Orijones of Amazonian Peru (de Castelnau 1851). Similar uses in this region have since been reported (Krukoff and Barneby 1970, Ayala Flores 1984), also for Brazil (Prance 1972), Colombia (García-Barriga 1992) and Ecuador (Cerón 1995). *Chondrodendron bioccai* G.Lusina (at present provisionally included in the synonym of *Curarea toxicofera*), was stated to be used as a principal source in the preparation of curare by the Maku Indians from Alto Rio Negro (Lusina 1954). Similarly, the sterile specimen (*Mexia 6321a*), previously identified as *Chondrodendron iquitanum* (Krukoff and Moldenke 1938) and later as *Curarea toxicofera* (Barneby and Krukoff 1971), was reported to be used as a source of dart poison by Indians in the Rio Santiago area. However, as the specimen in question was not available in this study, it is unclear to which of the two species it belongs.

### Geographical distribution and habitat

**Figure 9.**
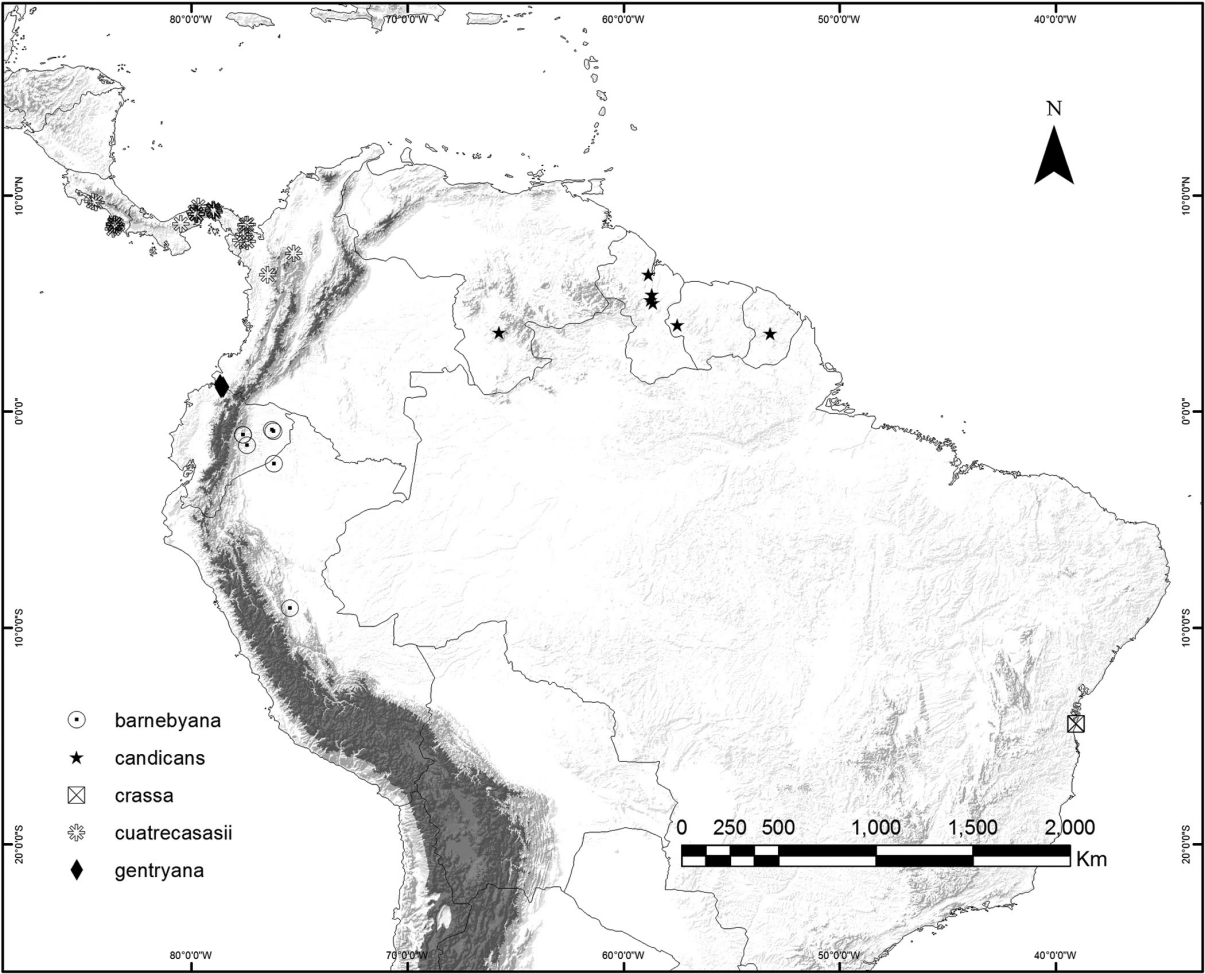
Geographic distribution of *Curarea barnebyana*, *C. candicans*, *C. crassa*, *C. cuatrecasasii* and *C. gentryana*.

*Curarea* is relatively widespread within tropical America, ranging from Costa Rica through Panama, to Bolivia, French Guiana, western Brazil and the Atlantic Forest of South-eastern Brazil (Figs 9, 21 and 25).

Species of *Curarea* are typically found in lowland tropical moist forests and lower montane forest (up to 1300 m elevation). They grow in periodically flooded forest (either *varzea* or *igapó*) to upland non-flooded forest, typically in primary forest, but are also occasionally present in secondary growth. The species are apparently locally rare and mainly allopatric, however, *Curarea aff. iquitana*, *C. toxicofera* and *C. tecunarum* were observed growing sympatrically in Peru.

### Phylogenetic affinities

In phylogenetic analyses of the family using plastid markers, *Curarea* was recovered as sister to *Sciadotenia*, in a clade that also includes *Chondrodendron* (Ortiz et al. 2016).

These affinities are congruent with earlier suggestions (Barneby and Krukoff 1971: Triclisieae *sensu* Diels 1910; Tiliacoreae *sensu* Forman 1982, Ortiz et al. 2016). With *Sciadotenia*, *Curarea* shares palmatinerved or plinerved venation and more or less sessile drupelets borne on elongate carpophores. With *Chondrodendron*, *Curarea* shares stomata aggregated in areolar depressions and raised above the epidermal cells on a short column of cells and a thyrsoid staminate inflorescence. In the study of Ortiz et al. (2016), *C. candicans* is recovered as sister to a clade in which *C. cuatrecasasii*, *C. barnebyana*, *C. tecunarum* and *C. toxicofera* *s.l.* are all unresolved (Fig. 1B).

Morphological characters suggest two species groups within *Curarea* (Table 5), although these are not congruent with the results from analysis of molecular data. In the group including *C. barnebyana*, *C. candicans*, *C. crassa* and *C. tecunarum*, the innermost pair of primary veins of mature leaves, which are in the canopy, conforms to an acrodromous perfect venation, with all but *C. tecunarum* also sharing staminate inflorescences with compact primary branches that have few branching orders. All the above listed species, except for *C. candicans*, share villous indumentum on their abaxial leaf surface and short subglobose carpophores. Wood and leaf anatomy features shared amongst these species are summarised in Table 5. *Curarea candicans* differs from the other species in the first group by its web-like indumentum and its unique drum-like carpophores, its narrow vessel elements being more similar to species of the second group. In the latter group, which includes *C. cuatrecasasii*, *C. gentryana*, *C. iquitana*, *C. tomentocarpa* and *C. toxicofera* (Table 5), the innermost pair of primary veins on mature leaves are acrodromous imperfect, the species have a strigillose-tomentellous indumentum on their abaxial leaf surface (web-like/tomentellous in *C. gentryana*), staminate inflorescences with lax primary branches, these with 4–6 branch orders, elongate carpophores and a few other anatomical features (Table 5). However, *C. cuatrecasasii* shares wide vessels with species in the first group.

### Multivariate analyses of *Curarea toxicofera* *s.l.*

**Figure 10.**
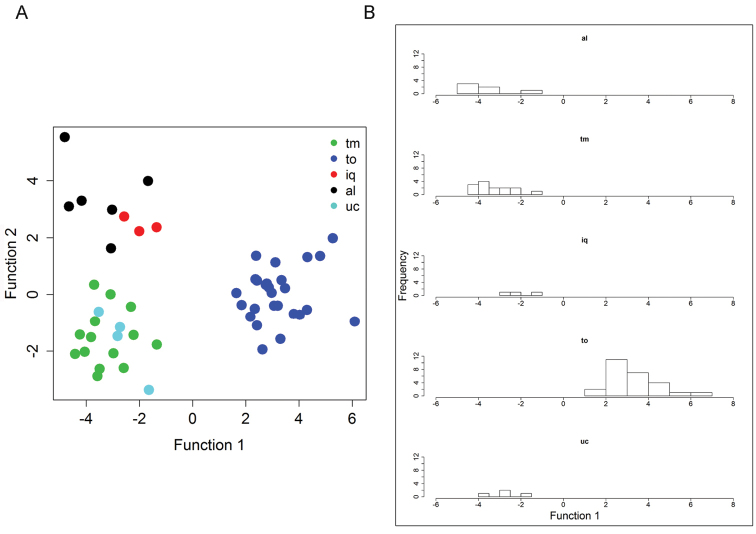
Linear discriminant analysis of characters from leaves, staminate inflorescences and staminate flowers of the five *a priori* groups of the *Curarea toxicofera* complex: **A** plot of scores of function 1 and function 2 **B** histograms of the first discriminant function values showing the positions of the five *a priori* defined groups, **al.** = allpahuayo, **iq** = iquitana, **tm** = tomentocarpa, **to** = toxicofera and **uc** = ucayali, respectively.

The principal component analyses (PCA) of the datasets indicates no clear pattern in the morphological variation of the *C. toxicofera* complex (not shown). Of the five datasets, the linear discriminant analyses (lda) of dataset 2, is presented here. The analysis of this latter dataset, which corresponds to a combination of leaves + staminate inflorescences + staminate flowers, was significant (Wilk’s lambda = 0.0043335, P < 0.001) and correctly assigned all individuals to their previously defined groups. The first two canonical discriminant functions recovered three clusters, namely groups **to**, **tm** + **uc** and **al.** + **iq** (Fig. 10A). Moreover, the toxicofera (**to**) group is recovered as an independent cluster by the first canonical discriminant function, whereas **al.**, **tm**, **iq** and **uc**, show various levels of overlap (Fig. 10B). The first two discriminant functions explained 68% and 16% of the variation respectively. Traits strongly correlated with the first discriminant function are inner whorl anther length, outer whorl anther length and inner whorl petal length. On the other hand, leaf width, leaf length and bract length are only weakly correlated with the second function. A cross validation (leave-one-out) classification showed that, overall, 70% of the specimens were correctly classified and individuals of the **tm** and **to** groups were 79% and 92% correctly classified, respectively.

Analysis of the subset of dataset 2, which excluded the toxicofera group, recovered three clusters, **al.**, **iq** and **tm** + **uc**, however the Wilk’s test was not significant (Wilk’s lambda = 0.000022, p = 0.2368). Similarly, a cross validation analysis indicated that, overall, only 37% of the specimens were correctly classified (not shown).

Hence based on the results of the lda analysis of the quantitative traits in dataset 2, a conservative approach is to recognise: the toxicofera (**to**) group, the tomentocarpa (**tm**) group (including the ucayali [**uc**] group) and the iquitana (**iq**) group (including the allpahuayo [**al.**] group) as separate entities (i.e. as three distinct species).

The groups, here evaluated, appear to vary across an ecological gradient and the **al.** and **uc** groups may represent separate entities that could be recognised when more material becomes available. Of the groups here recognised, one corresponds to *C. toxicofera* *s.s.*, the other two (**iq** and **tm**) were also previously described and two new combinations are here proposed.

## Taxonomic treatment

### Curarea

Taxon classificationPlantaeRanunculalesMenispermaceae

Barneby & Krukoff

Curarea Barneby & Krukoff, Mem. New York Bot. Gard. 22(2): 7. 1971.

#### Type.

*Curarea toxicofera* (Wedd.) Barneby & Krukoff.

#### Description.

*Lianas*, dioecious, growing in canopies or understory; branchlets villous, strigose or strigillose-tomentellous to glabrate, conspicuously ridged. *Leaves* simple, spirally arranged; blade narrowly to broadly ovate or elliptic, oblong, broadly elliptic, suborbicular, less frequently obovate; attached to the petiole at the base or scarcely subpeltate, chartaceous to coriaceous, base truncate or obtuse to rounded or slightly cordate, margin entire (or very rarely minutely undulate, weakly lobed or apically bilobed in *C. candicans*), apex retuse (cleft), acute or acuminate, rarely mucronulate, often cuspidate in young shoots, surfaces usually discolorous, especially when juvenile, lustrous and glabrous adaxially, but midrib sometimes sparsely tomentose, abaxially with finely silvery tomentellous, strigillose-tomentellous or web-like indumentum or the latter coarse and cream or dark brown villous, sometimes restricted to the areolae with age; palmati- to plinerved, (3–)5–7 main veins, the innermost pair perfect or imperfect acrodromous, secondary veins 0–2(–3) pairs or rarely absent, departing from midrib above the middle of the blade; petiole terete, tomentose to glabrate, pulvinate at both ends, apical pulvinus usually more conspicuous, the surface weakly rugulose, sometimes adaxially flattened. *Inflorescences* are solitary or fascicled, cauliflorous, axillary, supra-axillary or terminal on young shoots, basically thyrsi or simple dichasia; bracts subtending the primary branches lanceolate, narrowly ovate or ovate, markedly concave, fleshy, glabrous adaxially, variously pubescent abaxially, indument appressed or spreading. *Staminate inflorescence* with the axes sometimes conspicuously ridged; primary branches either lax, with several branching orders or compact and with few branching orders, pauci or multiflowered, variously pubescent. *Pistillate inflorescences*, with primary branches consisting of simple dichasia or these reduced to single flowers, mostly pauciflorous. *Flowers* are unisexual, actinomorphic and trimerous; pedicels conspicuous (absent in staminate flowers), terete, moderately slender, sometimes ridged, pubescent; bracteoles 1–2(4), usually early caducous, especially in staminate flowers. *Staminate flowers* are cream, whitish, greenish, yellowish, orangish, greyish or brownish; sepals 6(–9), in 2(–3) unequal whorls (spirally arranged), free, mostly ovate, narrowly ovate to obovate, oblong, rhombic, elliptic or suborbicular, inner whorl larger than outer one, both whorls weakly concave, scarce fleshy, glabrous adaxially and variously densely pubescent abaxially, tip of inner sepals erect or reflexed past anthesis; petals (5–)6 in 2, more or less similar whorls, smaller than the inner sepals, free narrow or broadly obovate-trilobed, obovate-rhombic or spatulate, weakly concave, membranous, glabrous adaxially, glabrous to densely tomentellous abaxially, base cuneate to distinctly clawed, lateral margins weakly to strongly inflexed and partially clasping the filaments, rarely those of the inner whorl adaxially connate, apex acute, obtuse, truncate or retuse; stamens (3–)6 in (1–)2 similar whorls, filaments free or variously connate, clavate, clavate-sigmoid or weakly terete, glabrous adaxially, glabrous or tomentellous abaxially, anthers basifixed, erect or weakly incurved, the connective is frequently thicker adaxially and forms a keel or a hump at the base or at the apex of the anther, less frequently forming a horn-like structure beyond the anthers; thecae latrorsely opening by longitudinal slits, sometimes anthers splitting into two halves (i.e. abaxial and adaxial) due to the reduction of the connective; pistillode 0. *Pistillate flowers* are green, yellowish or brownish coloured; sepals 6–9 in 2–3 unequal whorls, free, ovate-lanceolate, elliptic, ovate, broadly ovate, oblong or rhombic, glabrous adaxially, pubescent abaxially, tips of inner sepals usually reflexed after anthesis; petals 3(–6), free, spatulate, weakly concave, membranous, glabrous adaxially, glabrous to sparsely tomentellous abaxially; staminodes 0; carpels 3(–4), tomentose, free, sometimes proximal half coherent when young, style slightly tapering distally, stigma inconspicuous. *Infructescences* sometimes lenticellate or with the bark exfoliating; fruiting pedicels weakly clavate or terete, at times inconspicuous; carpophores are elongated and terete in understory species or short and subglobose in canopy species (drum-like in *C. candicans*). *Drupelets* oblongoid, ellipsoid, broadly obovoid or subglobose, sometimes weakly flattened laterally, sessile or rarely gradually narrowing toward the base, thus rarely forming a short stipe; stylar scar basal and frequently conspicuous; exocarp thin to thick (including the several layers immediately beneath the epidermal layer), coriaceous when dried, smooth, rugulose or muriculate, densely pilosulose, velutinous to glabrate, dull orange to yellow when ripe; mesocarp mucilaginous; endocarp thin and papyraceous or crustaceous, surface smooth or weakly ribbed along its long axis, less frequently there are also transversal ribs. *Seed* with endosperm absent, embryo hippocrepiform, cotyledons thick, accumbent, sometimes unequal.

#### Key to the species of *Curarea*

**Table:** 

1	Abaxial surface of leaf blades covered with coarse, golden, cream, dark brown or greyish tomentellous indumentum	**2**
–	Abaxial surface of leaf blades covered with finely silvery-tomentellous, strigillose-tomentellous or web-like indumentum	**4**
2	Staminate inflorescences with primary branches lax, these with (2–)4 or more branching orders	**6. *C. tecunarum***
–	Staminate inflorescences with primary branches compact, these with 0–2 branching orders	**3**
3	Staminate inflorescences with golden indumentum; drupelets broadly obovoid, weakly laterally compressed, centric or only weakly eccentrically attached; mature leaves broadly ovate to suborbicular; Atlantic Forest in Southeastern Brazil	**3. *C. crassa***
–	Staminate inflorescences with dark brown indumentum; drupelets narrowly obovoid to ellipsoid, laterally compressed, strongly eccentrically attached; mature leaves narrowly ovate to elliptic; foothill forests in Ecuadorian Amazon and eastern Peru	**1. *C. barnebyana***
4	Staminate inflorescences with condensed primary branches; carpophores discoid-shaped	**2. *C. candicans***
–	Staminate inflorescences with lax primary branches; carpophores inconspicuous or elongated	**5**
5	Staminate flowers with lateral margins of inner petals incurved and connate; stamens frequently 3; drupelets obovoid, ca. 4 × 2.8 cm, on inconspicuous carpophores	**5. *C. gentryana***
–	Staminate flowers with lateral margins of inner petals incurved but free; stamens frequently 6; drupelets ellipsoid or oblongoid, 1.3–3.2 × 0.8–1.8 cm, on conspicuously elongated carpophores	**6**
6	Staminate inflorescence with filiform primary branches	**4. *C. cuatrecasasii***
–	Staminate inflorescences with moderately stout primary branches	**7**
7	Staminate inflorescences with rufescent or silvery hispidulous indumentum; drupelets frequently with strongly muriculate surface	**8. *C. tomentocarpa***
–	Staminate inflorescences with greyish or brownish strigillose indumentum; drupelets with relatively smooth surface	**8**
8	Staminate flowers up to 1.8 mm long, anthers up to 0.3 mm long with connective strongly protruding apically either conically or as a horn, less frequently humped; endocarps 1.7–2.4 cm long	**7. *C. iquitana***
–	Staminate flowers up to 2.4 mm long, anthers up to 0.6 mm long with connective usually not apically protruding or overgrowing as a hump when older; endocarps 0.7–0.9 cm long	**9. *C. toxicofera***

### 1. Curarea barnebyana

Taxon classificationPlantaeRanunculalesMenispermaceae

R.Ortiz sp. nov.

urn:lsid:ipni.org:names:77185798-1

Figs 11 , 12

#### Diagnosis.

The species is distinguished by its coriaceous leaves that are narrowly ovate or elliptic, with dense brown villous indumentum, primary branches of the staminate inflorescence contracted/condensed and drupelets broadly obovoid or ellipsoid.

#### Type.

Ecuador. Pastaza: Pastaza Canton, pozo petrolero “Moretecocha” de ARCO 75 km al. este de Puyo, bosque húmedo tropical, 01°34'S; 77°25'W, 580 m, 4–21 Oct 1990, (♂ fl), *Gudiño, Quelal & Caiga 952* (holotype: MO!; isotypes: QCNE!, NY!, US!).

#### Note.

Ortiz-Gentry (2000; unpublished MS thesis) provisionally used the epithet *phaeofusca* for this taxon.

#### Description.

**Figure 11.**
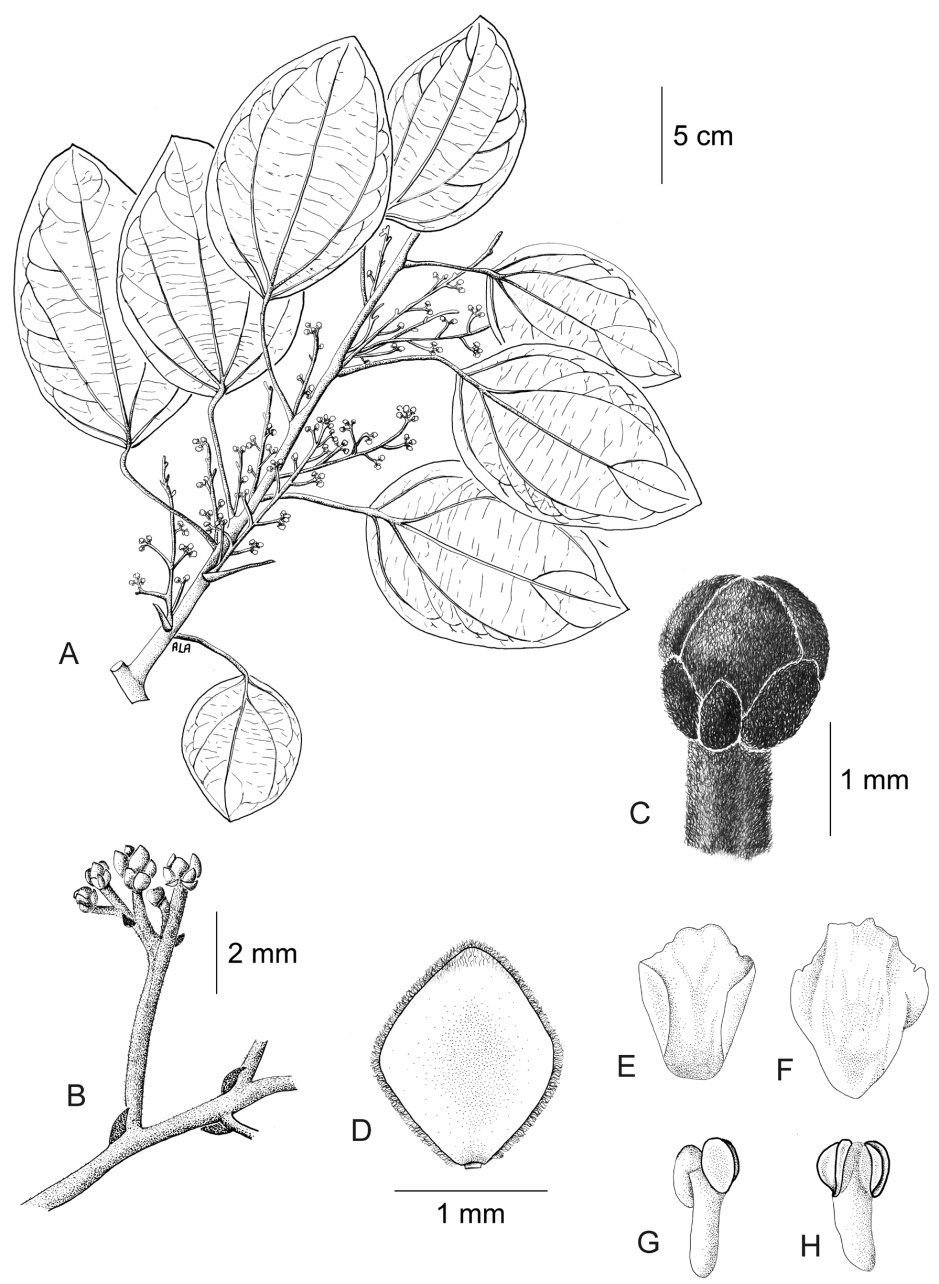
*Curarea barnebyana* staminate plant: **A** flowering branch **B** detail of inflorescence **C** flower bud **D** inner sepal, adaxial surface **E–F** outer and inner petals, adaxial and abaxial surfaces **G–H** outer stamens, latero-adaxial and abaxial surfaces (based on *Gudiño et al. 952*).

**Figure 12.**
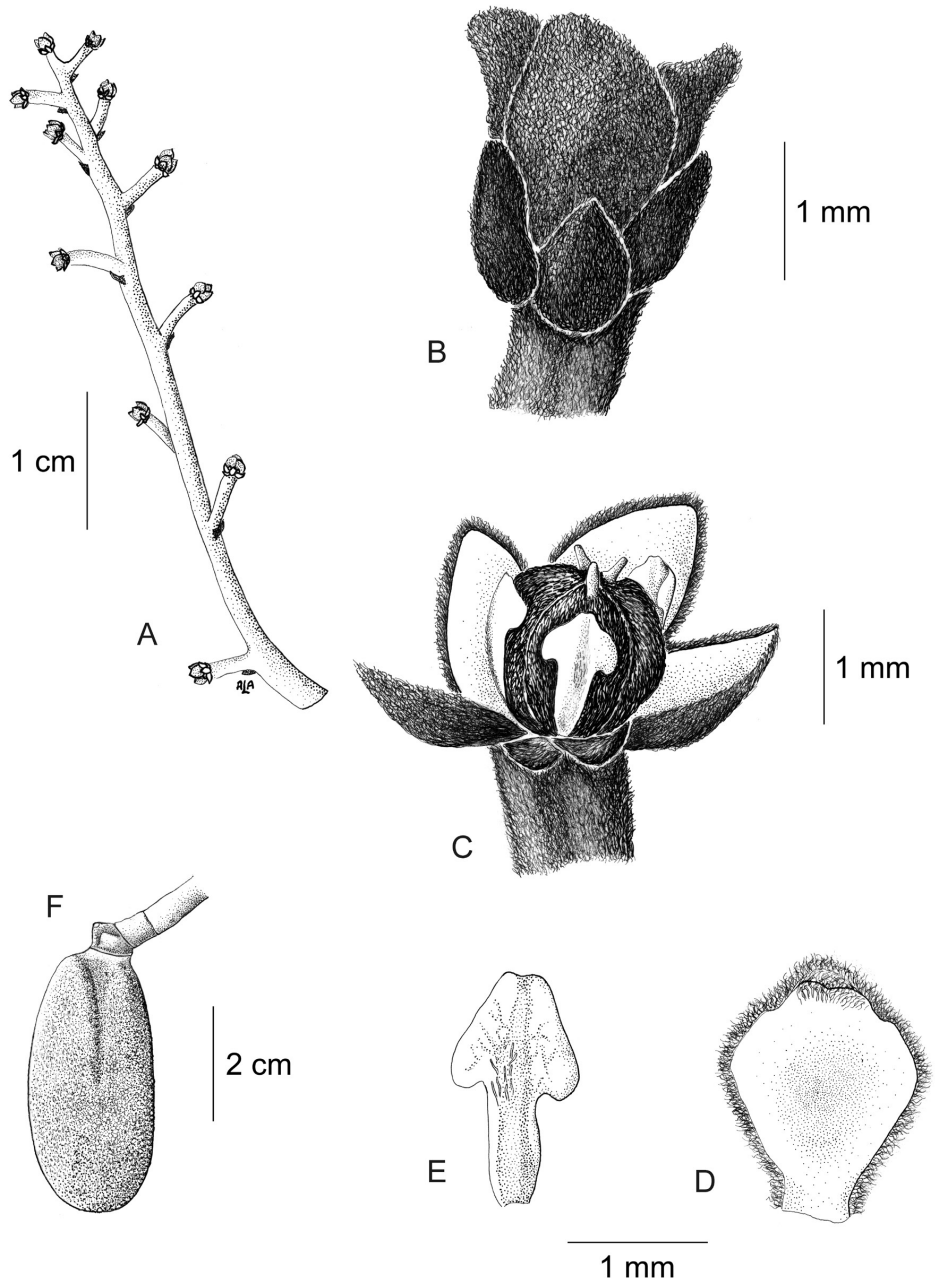
*Curarea barnebyana* pistillate plant: **A** inflorescence **B** flower **C** opened flower showing the petals and carpels **D** innermost sepal, adaxial surface **E** petal, abaxial surface **F** fruit, lateral surface. (**A–E** based on *Ortiz & Vargas 200* **F** based on *Dik 1213*).

Large canopy *lianas* about 20–30 m tall; older stem 6–20 cm wide, strongly flattened, with shallow lengthwise fissures; bark dark brown; branchlets densely coarsely dark brown villous tomentose. *Leaves*: blades 9–15 × 6–11 cm, ovate to elliptic, subcoriaceous to coriaceous when mature or when exposed to direct sunlight in the canopy; surfaces discolorous, lustrous and glabrous adaxially, coarsely dark brown or cream villous abaxially, trichomes concealing the epidermis at all stages, base obtuse to rounded, apex acuminate or retuse, cuspidate when juvenile, 5(7) palmati- or plinerved, innermost pair of main veins acrodromous perfect on mature leaves, acrodromous imperfect on juvenile ones, midrib and secondary veins slightly impressed adaxially, conspicuously raised abaxially, secondary veins 1–2 pairs, arising above the middle of the blade, veinlets immersed on the adaxial surface, raised abaxially; petioles 2.5–8.5(–15) cm long, terete, densely dark brown villous, apical pulvinus terete, rugulose. *Staminate inflorescences* solitary, axillary or slightly supra-axillary, thyrsi (Fig. 11A–B), densely dark brown villous; axes 7.4–9 cm long; primary branches, 1.2–1.9 cm long, with compact and reduced (0–1) branching orders; bracts 0.9–1.1 mm long, narrowly ovate, concave, fleshy, glabrous adaxially, dense dark brown villous abaxially. *Pistillate inflorescences*, solitary or fascicled, axillary, few-flowered thyrsi, the primary branches reduced to single flowers (Fig. 12A), indumentum as on staminate inflorescences; axes 4.8–5.3 cm long, terete or angular; bracts 0.7–1.1 mm long, ovate, concave, fleshy, glabrous adaxially, brown villous abaxially. *Staminate flowers* 1.4–1.7 mm long, cream to brownish; pedicels ca. 0.9–1.9 mm long, terete, indumentum as on the axis; bracteoles 1–2, 0.3 × 0.1–0.2 mm, ovate to oblong, fleshy, glabrous adaxially, brown tomentose abaxially; sepals 6, glabrous adaxially, brown tomentose abaxially; outer sepals 0.6–0.7 × 0.3–0.5 mm, ovate or oblong, base truncate, apex acute; inner sepals 1.3–1.5 × 1–1.3 mm, obovate or obovate-rhombic, base obtuse, apex obtuse or rounded, tip of inner sepals erect but not reflexed past anthesis; petals 6, 0.6–0.7 × 0.3–0.5 mm, inner ones a little narrower, obovate-rhombic, weakly concave, glabrous adaxially and abaxially, base obtuse to cuneate-truncate (shortly clawed), lateral margins inflexed, partially clasping the filaments, apex obtuse, acute or weakly retuse; stamens 6; filaments 0.4–0.5 mm long, clavate, moderately thick, free or shortly connivent, glabrous; anthers 0.2–0.3 mm long, erect, connective forming a conical-shaped protrusion adaxially, (Fig. 11G), not protruding beyond thecae apically. *Pistillate flowers* 1.6 mm long, brownish; pedicels 3.2–3.6 mm long, terete; bracteoles 2, 0.4–0.5 × 0.3 mm, ovate, fleshy, glabrous adaxially, brown tomentose abaxially; sepals 6–9, in 2–3 whorls, weakly concave, slightly fleshy to fleshy, glabrous adaxially, brown tomentose abaxially; outer sepals, 0.6–0.9 × 0.3–0.6 mm, ovate or oblong, base truncate, apex obtuse; middle sepals ca. 0.9 × 0.8 mm, obovate, base and apex obtuse; inner sepals 1.1–1.6 × 1–1.5 mm, weakly obovate or elliptic, base and apex obtuse, tips erect to reflexed past anthesis; petals 3, 0.8–1.3 × 0.6–1.4 mm, spatulate, weakly concave, glabrous adaxially, glabrous or sparsely tomentose abaxially, base clawed, apex retuse; carpels 3, 0.4–0.9 × 0.3–0.7 mm, dark brown villous tomentose, indumentum appressed-ascending; style 0.4–0.5 mm long. *Infructescences* axes 2–6.7 × 0.3–0.5 cm, indumentum as on pistillate inflorescences; fruiting pedicel 0.3–0.6 cm, clavate; carpophores triangular or subglobose, ca. 3.6 mm long, convex at apex, dark brown villous. *Drupelets* 2.9–4.5 × 2.3 3.1 cm, yellow or orange when ripe, narrowly obovoid to ellipsoid (Fig. 12F), weakly laterally flattened, at times gradually attenuate toward the base; base obtuse, strongly eccentrically attached; stylar scar not apparent; exocarp 4–6 mm thick, surface rugose, dark brown villous tomentose, granular when dried; mesocarp thin, mucilaginous; endocarp 2.5–3.5 × 1.5–1.9 cm, chartaceous, surface smooth. *Seeds* with embryo 5.8–6.9 cm long, cotyledons slightly unequal.

#### Distribution and ecology.

Andean foothills of eastern Ecuador and eastern Peru (Fig. 9), at elevations of 200–450 m in tropical wet forest. Staminate flowering specimens were collected in January, June and October; fruiting and old pistillate flowering specimens were collected in January and June.

#### Common names and uses. Ecuador.

“jondomebo” (Huaorani) (*Gudiño et al. 952*, ♂ fl).

#### Eponymy.

The specific epithet honours the late Dr. Rupert C. Barneby whose work has laid the foundations for all subsequent taxonomic studies of neotropical Menispermaceae.

#### Conservation status.

The calculated Extent of Occurrence (EOO) based on ten collections representing six localities of *C. barnebyana* resulted in 72,674 km^2^, whereas the Area of Occupancy (AOO) was estimated as 24 km^2^. Of the six sub populations representing four locations, two of the latter are found nearby private or national protected areas in eastern Ecuador and is very likely that more individuals will be found within these areas. Of the two other locations found across the border, in Peru, one of them is in a rather continuous tract of forests, whereas the other is found in a nearby area where there is increasing deforestation, which may result not only in a decline in habitat quality, but also likely reduction of the geographic range of the species in the future. Based on these considerations, *C. barnebyana* is assigned a preliminary status of “Vulnerable ” [VU, A3c + B1b(i,ii,iii,iv) + B2b(i,ii,iii,iv)].

#### Discussion.

*C. barnebyana* is recognised by its large obovoid or ellipsoid and weakly laterally flattened drupelets covered with a dark brown villous tomentose indumentum and borne on claviform fruiting pedicels. Similar indumentum is found in *C. tecunarum*, but the primary branches of the staminate inflorescences of the latter are laxly branched, while the primary branches of the inflorescences of *C. barnebyana* are condensed similar to those of *C. crassa* and *C. candicans* (see discussion under *C. crassa*). Shared anatomical features amongst these species, which make up group 1, are summarised in Table 5. In the fruiting condition, *C. barnebyana* loosely resembles *C. crassa*, but it is readily distinguished by its relatively long (ca. 8 cm in length) infructescence axis, clavate fruiting pedicels and narrowly obovoid to ellipsoid drupelets that are weakly laterally flattened and have a dark brown indumentum. *Curarea crassa*, on the other hand, has a short (ca. 0.5–2 mm long) infructescence axis, terete fruiting peduncles and broadly obovoid drupelets with a dense, golden villous indumentum.

#### Paratypes.

**ECUADOR**. **Napo**: Parque Nacional Yasuní, Pozo Petrolero Daimi 2, 00°55'S; 076°11'W, 200 m, 26 May–8 Jun 1988, (fl bud), *Cerón & Hurtado 4094* (MO!, QCNE!); Canton Tena Estación Biológica Jatun Sacha, 8 km al. este de Misahuallí, bosque muy húmedo tropical, 01°04'S; 077°36'W, 400 m, 23–31 Jan 1989, (imm fr), *Cerón 6008* (G!, MO!, NY!, QCNE!); Reserva Etnica Huaorani, Carretera y oleoducto de Maxus en construcción, km 86–89, 00°51'S; 76°15'W, 260 m, 25–30 Mar 1994, (mat fr), *Dik 1213* (MO!, QCNE n.v.); Canton Tena Estación Biológica Jatun Sacha, 450 m, 7 Jan 1989, (imm fr), *Neill 8705* (G!, NY!, QCNE!); ibid., (♀ fl & imm fr), *Neill 8712* (G! [2], MO!); Estación Biológica Jatún Sacha, 28 Jun 1996, (♀ fl & imm fr), *Ortiz & Vargas 194* (MO! [2]); ibid., 29 Jun 1996, (♀ fl & imm fr), *Ortiz & Vargas 195* (MO!); ibid., 3 Jul 1996, (♀ fl & imm fr), *Ortiz & Vargas 200* (MO!).

**PERU**. **Loreto**: Campamento Forestal, 16 km from the Ecuador border near Río Conventes, overgrown road cut margins, [02°25'S; 076°10'W], 12 Apr 1979, (old fr), *Aronson & Rodrigues 859* (MO!). **Ucayali**: Prov. de Padre Abad, distrito de Padre Abad, carretera al. caserío San Miguel y Mapuya, 12–17 km de Aguaytía, bosque primario con abundante luz solar, 09°05'S; 075°26'W, 350 m, 8 Oct 2004, (♂ fl), *Schunke & Graham 16307* (F n.v., MO!).

### 2. Curarea candicans

Taxon classificationPlantaeRanunculalesMenispermaceae

(Rich. ex DC.) Barneby & Krukoff

Figs 13A , 14

Curarea candicans (Rich. *ex* DC.) Barneby & Krukoff, Mem. New York Bot. Gard. 22(2): 12. 1971.
Abuta candicans Rich. *ex* DC., Syst. 1: 543. 1818. Type: French Guiana. Cayenne: “Cayensibus dicitur, genus affine Cissampelos”, no date [1781–1789], (sterile), *L.C. Richard s.n*. (lectotype, designated here [or perhaps holotype]): P–Jussieu Herbarium 10832, photocopy of microfiche P!, IDC microfiche 6206. 803.I). Guyana. Demerara: Mabura Hill Concession, ridgetop ecotone between Wallaba and Purpleheart forest, 05°25'N; 058°40'W, 50 m, 20 Nov 1986, (♂ fl), *Pipoly & Boyan 8982* (epitype, designated here: MO!; isoepitypes: NY!, US!). Note: Following Art. 9.8 of the Melbourne Code (MacNeill et al. 2012), I am here designating an epitype to serve as an interpretative type of Abuta candicans, whose sterile condition makes it ambiguous for identification purposes.
Sciadotenia candicans (Rich. *ex* DC.) Diels, in Engl. Pflanzenr. 4(94): 87. 1910. Type: Based on Abuta candicans Rich. *ex* DC.
Chondrodendron candicans (Rich. *ex* DC.) Sandwith, Bull. Misc. Informat. Kew. 1930: 342. Type: Based on Abuta candicans Rich. *ex* DC.
Cocculus dichroa Mart., Flora 24, Beibl. II: 46. 1841. Type: Brazil. Para: Habitat in silvis, no date, (sterile), *Martius s.n.* (lectotype, designated here: M! [image seen]; isolectotype: B! frag., likely of M).
Sciadotenia leucophylla Miers, Contr. Bot. 3: 344. 1871. Type: In Guiana [Guyana]. Guiana Batavana: no date, (sterile), *Anderson s.n.* (holotype: BM! [BM000071492]).
Abuta limaciifolia Diels, in Engler, Pflanzenr. IV.94(Heft 46): 194. 1910. Type: Brazil. Para: Peixe Boi, [25] July 1907, (♂ fl bud), *Rodr*. *Sigueira s.n.* [Herbário Amazônico Museu Paraênsis (Museu Goeldi) MG-8266], (lectotype designation effected by Krukoff and Moldenke 1938: 20: B!, F neg. 4993; isolectotypes: BM! [BM000071503], MG! [image seen]). Note: The F negative of B specimen has two annotations, one at the bottom right that reads: Abuta limaciifolia Diels and a second one to the bottom left that reads: “Anomospermum limaciifolium Diels sp. nov.”, a non-published name, this later annotation is missing in the B specimen available in this study, which may suggest that this second annotation label was not glued-down or the existence of another specimen at B. However, the B specimen I studied appears in every other feature to be the source of the F photograph. Additionally, the measurements included in the original description of the species, suggest that Diels did not examine the duplicates at BM and MG, hence the lectotype designated is the B specimen examined here. The citation by Diels of “Herbário Amazônico Museu Paraênsis” is in reference to the label of the material in B, not to a sheet in MG.
Chondrodendron limaciifolium (Diels) Moldenke, in Krukoff & Moldenke, Brittonia 3: 20. 1938. Type: Based on Abuta limaciifolia Diels.
Abuta (?) pullei Diels, Rec. Trav. Bot. Neerl. 22: 348. 1925. Type: Suriname. Without locality, 4 Sep 1920, (sterile), *Pulle 408* (lectotype designation effected by Krukoff and Moldenke 1938: 18: U! [U-30956, photographs at F!, G!, GH!, MO!; isolectotypes: B!, frag., U! [U-30955]). Note: the original material of Abuta ? pullei comprises juvenile leaves and is mounted on two sheets at U. Both appear to have Diels’ handwriting and the sheet U-30956 has one, more or less, complete leaf and two fragmentary leaves. The second sheet, labelled as U-30955, is a much younger leaf and it appears that its measurements were not included in the description of the species. Hence the U-30956 specimen has been considered the holotype and annotated as such by previous authors, a designation that is followed in this study.

#### Description.

**Figure 13.**
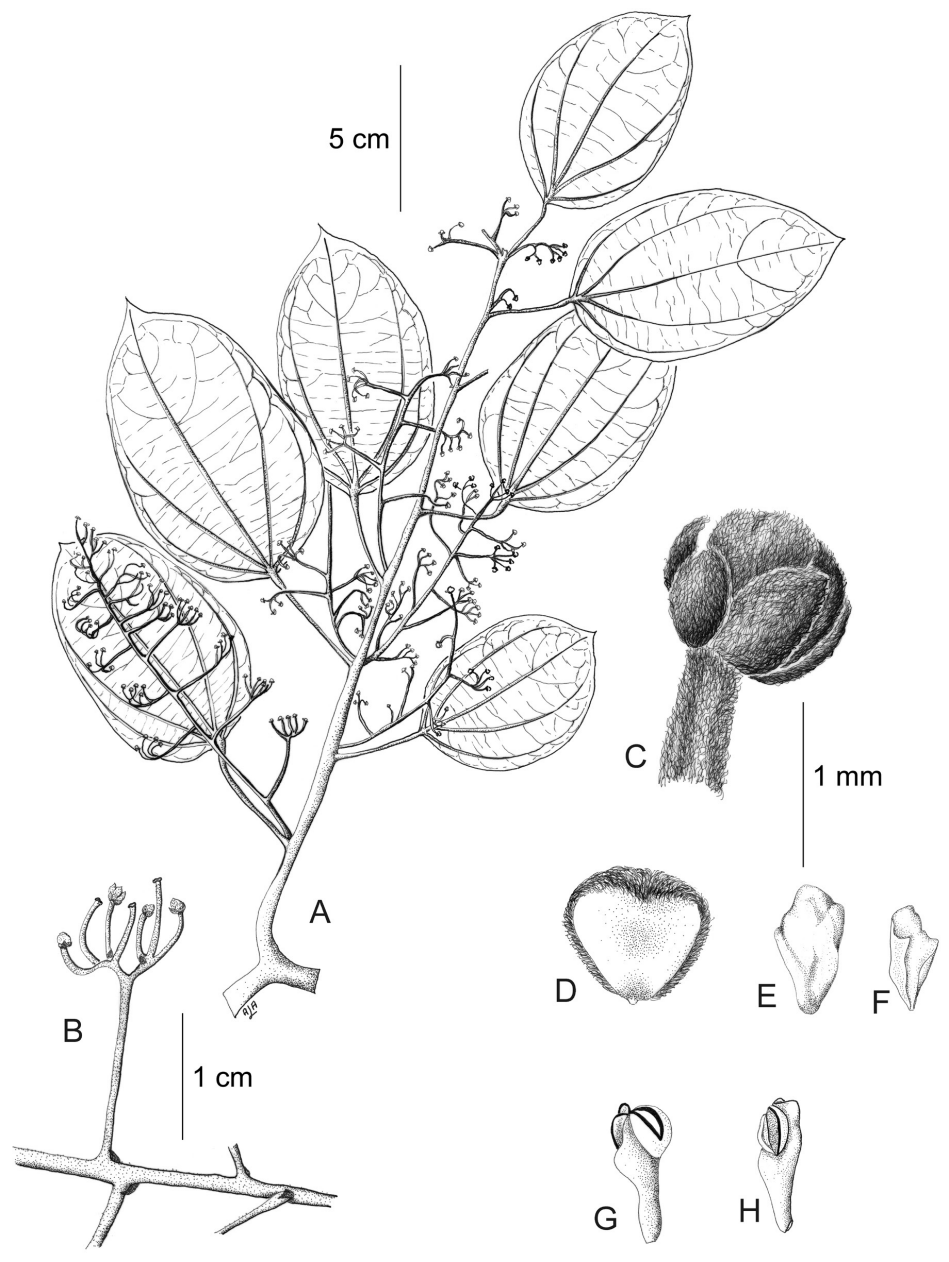
*Curarea candicans* staminate plant: **A** flowering branch **B** detail of inflorescence **C** flower bud **D** inner sepal, adaxial surface **E–F** outer and inner petals **G–H** outer stamens, latero-adaxial and lateral surfaces (based on *Pipoly & Boyan 8982*).

**Figure 14.**
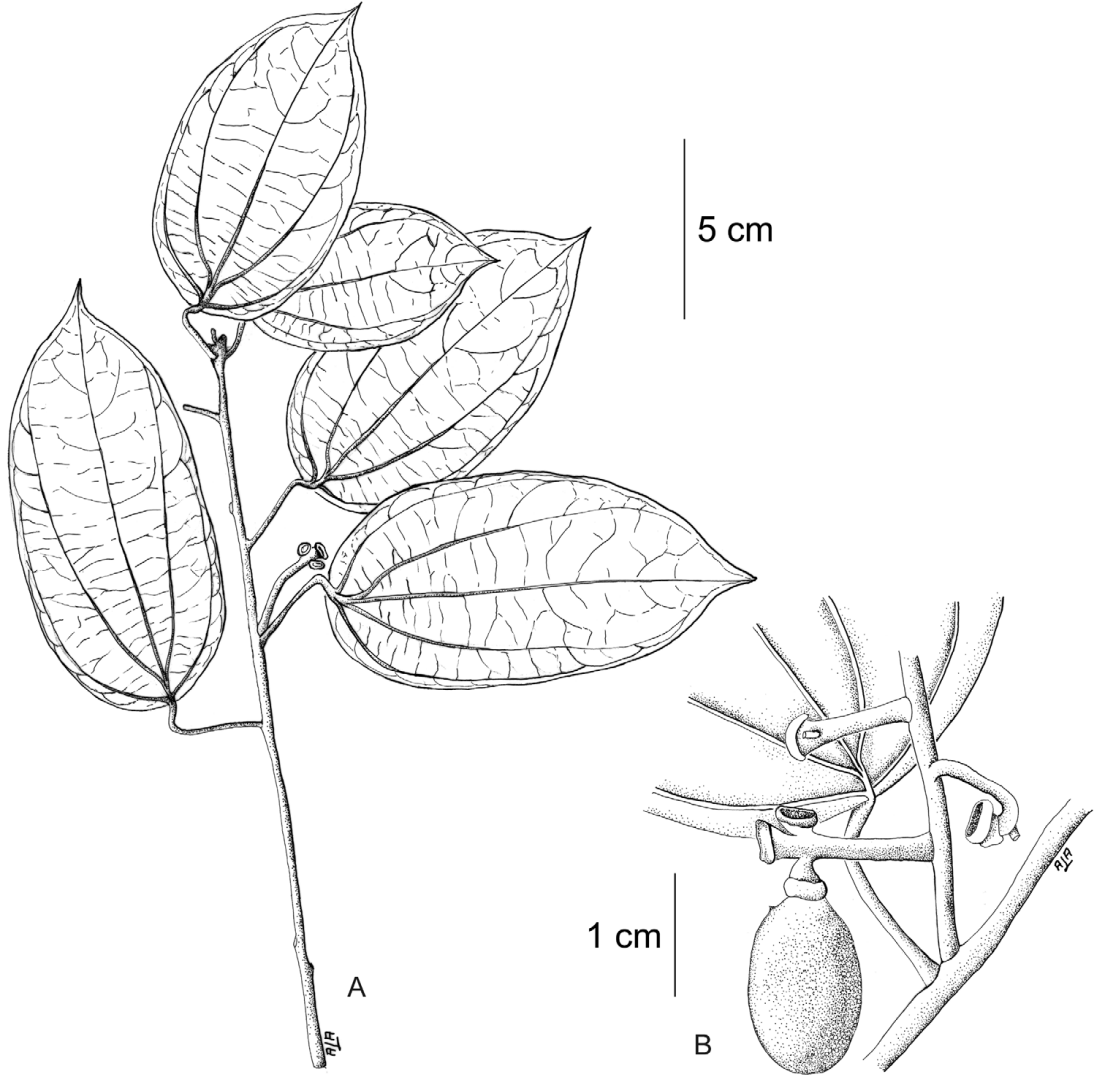
*Curarea candicans* pistillate plant: **A** branch showing fruiting peduncle with no fruits **B** fruiting branch (based on [*Jardim Botanico do Rio RB-19508*]).

Large canopy *lianas* ca. 25 m tall; older stem flattened (width unknown); bark dark brown, with shallow lengthwise fissures; branchlets densely brownish to silvery strigose. *Leaves*: blades 9–23 × 5–12 cm, elliptic, oblong or narrowly ovate, subcoriaceous to coriaceous when mature and up in the canopy; surfaces conspicuously discolorous when juvenile, lustrous and glabrate to glabrous when mature adaxially, silvery web-like indumentum concealing the abaxial epidermis when juvenile, with few brownish trichomes on main veins, densely tomentellous with age, indumentum usually confined to the areolae, base obtuse, rounded or cuneate, margin entire, (minutely undulate –“*crispatulo subdentato*”– (de Candolle 1818: 543), apex acuminate (bilobulate), cuspidate when juvenile, usually 3–5(7) palmatinerved, less frequently plinerved, innermost pair of main veins acrodromous perfect on mature leaves, usually acrodromous imperfect on leaves from young shoots, midrib immersed or raised adaxially, raised abaxially, secondary veins 0–3 pairs, usually arising above the middle of the blade, veinlets weakly prominent adaxially in juvenile leaves, immersed on mature ones, always raised abaxially, sparsely silvery-tomentose adaxially when juvenile; petioles 2.5–16 cm long, the smaller sizes are frequently associated with canopy leaves and thus fertile plants, silvery, greyish or rufous strigillose-tomentellous, the trichomes appressed or ascending, glabrate with age, apical pulvinus conspicuous, rugulose, shallowly grooved adaxially. *Staminate inflorescences* solitary or fascicled, slightly supra-axillary or axillary, narrowly branched thyrsi (Fig. 13A–B), densely silvery to greyish or rufescent strigillose tomentellous, conspicuously ridged; axes (1.2–)6.5–10.6 cm; primary branches 0.6–2.1 cm long, compact and 1–2(–3) branching orders (Fig. 13B); bracts 0.5–0.9 mm long, ovate, concave, fleshy, glabrous adaxially, abaxially indumentum as on inflorescence. *Pistillate inflorescences* unknown. *Staminate flowers* 1.2–1.3 mm long, greenish, yellowish or brownish; pedicels 0.9–5.6 mm long, conspicuously ridged, indumentum as on staminate inflorescence; bracteoles 1–3, 0.3–0.5 × 0.2–0.3 mm, ovate, ovate-lanceolate or oblong, fleshy, glabrous adaxially, silvery tomentellous abaxially; sepals 6, usually 2-whorled (spirally arranged (?) and 8 in number, *Steyermark et al. 125686*), glabrous adaxially, silvery tomentellous abaxially; outer sepals 0.7–1.2 × 0.2–0.6 mm, narrowly ovate or ovate-lanceolate, base obtuse or truncate, apex acute (middle sepals ca.1–1.2 × 0.7 mm, obovate, base obtuse, apex rounded); inner sepals 0.9–1.6 × 0.8–1 mm, obovate to suborbicular, base obtuse, apex acute or obtuse (weakly retuse), tip of inner sepals erect to reflexed past anthesis; petals 6, 0.4–0.8 × 0.2–0.6 mm, inner ones slightly shorter and narrower, obovate to obovate-trilobed, weakly concave, membranous, glabrous adaxially, glabrous to sparsely tomentellous abaxially, base cuneate, lateral margins inflexed, partially clasping the filaments, apex obtuse or truncate; stamens 6, filaments 0.2–0.6, mm long, inner ones slightly longer, clavate-terete, moderately thick, free or connivent, glabrous; anthers 0.1–0.3 mm long, erect, connective slightly protruding apically, thicker adaxially and forming a protruding keel at the base of the anthers or shortly overgrowing thecae when older (Fig. 13G–H). *Pistillate flowers* unknown. *Infructescences* axes 2.5–2.7 × 0.3 cm, thyrsi (Fig. 14A–B), velutinous; fruiting pedicel 0.5–1.1 cm long, terete; carpophore drum-like, ca. 1.3 mm long, apically weakly concave, velutinous. *Drupelets* ca. 2.5 × 1.7 cm, (colour when ripe unknown), broadly ellipsoid to subglobose (Fig. 14B), slightly eccentrically attached, base obtuse; stylar scar weakly protruding; exocarp 2 mm thick, surface rugulose or weakly muriculate, velutinous, granular when dried; mesocarp not seen; endocarp papyraceous, surface smooth. *Seeds* and embryo not seen.

#### Distribution and ecology.

North-eastern South America, from southern Venezuela, Guyana, Suriname, French Guiana and northern Para in Brazil (Fig. 9), in Terra Firme forest from near sea level to 710 m in Lely Mt., Suriname. Staminate flowering specimens were collected in January, July, October and November; the single fruiting specimen was collected in February.

#### Common names and uses.

**Guyana**: “teteabo (Arawak), “granny’s backbone” (Creole), (Sandwith 1930, *Sandwith 561*, ♂ fl); used in the preparation of “warrou’s other kind of poison” (*Jenman 5199*, ♂ fl). **Suriname**: “dobroedoea” (*Vreden 11706*, st). **Venezuela**: “shiña-ten” (*Steyermark et al. 125686*, ♂ fl bud).

#### Etymology.

The epithet “candicans” doubtlessly stems from the silvery indumentum on the abaxial surface of leaves, which is dense and matted in young individuals.

#### Conservation status.

Analysis of the seven collections representing seven localities resulted in an Extent of Occurrence (EOO) of 209,650 km^2^ and an Area of Occupancy (AOO) of 28 km^2^. Of the seven subpopulations, the most recent collection –a sterile specimen– was made in 2004. While the species has not been collected during the past decade, which may be suggestive of population decline, it is also likely that, due to its climbing habit, the species might have been overlooked by collectors. Additionally, one of the seven individuals occurred in a nature reserve in Venezuela and the locality where one collection was made in French Guiana in the early 1990s, has also become a nature reserve. Based on these observations and the results of the assessment, *C. candicans* is assigned a preliminary status of “Least Concern” (LC).

#### Discussion.

The drum-like carpophores of *C. candicans* (Fig. 14B) are unique in the genus. In all other species, these are elongated or subglobose. Vegetatively, *C. candicans* is also distinctive on account of the sparse appressed or ascending brownish trichomes on main veins and dense silvery web-like indumentum on the abaxial surface of leaves from young shoots. This indumentum later changes to a tomentellous cover that usually becomes restricted to the areoles with age. The only other species with similar web-like indumentum is *C. gentryana*, described below, but this lacks the brownish appressed trichomes on the main veins of *C. candicans* and, moreover, its web-like indumentum persists on old leaves. When leaves of *C. candicans* are tomentellous and this indumentum is not yet restricted to the areoles, they are indistinguishable from those of *C. toxicofera* and *C. cuatrecasasii*. However, neither of the two occurs in the Guianas and both have laxly branching rather than compact primary branches of the inflorescences of *C. candicans*.

In a family-wide phylogenetic analysis, *C. candicans* is recovered as sister to the remaining sampled species and support for this placement is high (Ortiz et al. 2016). It shares similar narrowly branched secondary axes in the staminate inflorescences with *C. crassa* and *C. barnebyana*.

The type of *Abuta candicans*, the basionym of *Curarea candicans* (Rich. *ex* DC.) Barneby & Krukoff, is a sterile and unnumbered Richard collection from French Guiana (deposited in the P herbarium). Although the specimen in question shows features usually not associated with the remaining specimens referred to *C. candicans*, such as leaves with bilobulate apex, minutely undulate margins and penninerved venation, these features have sporadically been observed in a few sterile specimens of other *Curarea* species, although not in the same combination on the same specimen. I follow earlier workers in accepting this sterile specimen as the type of the basionym of *Curarea candicans*; as described by de Candolle (1818), the leaves are abaxially “*glabris candicantibus*”, hence the specific epithet, which highlights a distinctive feature of this species. However, in order to unequivocally fix the application of the name, an epitype is being designated is this study.

On another unnumbered collection of Richard, also at P, one of the four labels has the annotation “Type coll. 2” made by Krukoff in 1968. This specimen is rather dissimilar from the type material: its leaf blades are elliptic, the apices are not bilobed and the secondary veins arise beyond the middle of the leaf, towards the apex; however, the abaxial surface is whitish. A comparison of this second specimen with other collections from the region suggests that it is conspecific with the fertile representatives of the species. However, there is no indication in de Candolle’s original description of the existence of another specimen and he described the lamina as having a bilobed apex; thus this second specimen should not be considered part of the original material.

In the protologue, von Martius (1841) contrasted *Cocculus dichroa* with *Abuta candicans*. Eichler (1864) considered the two conspecific and placed *Cocculus dichroa* as a synonym. Similarly, Miers (1871: 392) in listing Abuta ? candicans as a presumed species, imperfectly known species in current terminology, he also listed *Cocculus dichroa* as synonym of *A. candicans*. Miers also noted that, due to its lack of inflorescences, it was uncertain whether the specimen in question belonged in *Abuta* or in *Chondrodendron*. Subsequently, *Cocculus dichroa* Mart., has been considered a synonym of *Sciadotenia candicans* (Rich. *ex* DC.) Diels (Diels 1910), *Chondrodendron candicans* (Rich.) Sandwith (Sandwith 1930) and, more recently, *Curarea candicans* (Rich. *ex* DC.) Barneby & Krukoff (Barneby and Krukoff 1971).

The sterile type material of *Cocculus dichroa* at M, image from JStore, has the leaf blades ovate, with a long-acuminate apex and the adaxial surface somewhat bullate. While ovate and long-acuminate leaves are characteristic of juvenile leaves of all species of *Curarea*, however a somewhat bullate adaxial surface has not been observed in *C. candicans*. This vegetative feature might turn out to be a distinguishing character when studied in more specimens and the extent of morphological variation *C. candicans* is better understood. At this time however, *C. candicans* is the only other species known to occur in Para and, in this study, I hesitantly follow earlier workers in the family in including *C. dichroa* as a synonym of *C. candicans*.

#### Specimens examined.

**BRAZIL. Pará**: Santa Isabel. Ea. de F. [Estrada de Ferro] de Bragança, 15 Feb 1909, (detached old fr), *collector unknown*, (RB n°. 19508!).

**FRENCH GUIANA. Cayenne**: *Richard s.n.*, no date, (st), (P!). **Saül**: Vicinity of Eaux Claires, Sentier Botanique, between Crique Tortue and Split in trail, 03°37'N, 053°12'W, 200-400 m, 1 Nov 1992, (♂ fl), *Mori et al. 22743* (NY!).

**GUYANA. Demerara**: Mabura Hill, Ekuk compartment, mixed forest on loamy sand, 05°10'N; 058°45'W, 12 Oct 1989, (♂ buds & fl) *Jansen-Jacobs et al. 1995*, (NY!, U!); Upper Demerara-Berbice, Pibiri Research Site, 53 km S of Mabura Hill, 05°01'N; 058°37'W, 2 Feb 2004, (st), *Torke et al. 310* (MO!). **Cuyuni-Mazaruni Region**: Kartabo, Willems Timber Concession, Wallaba (Eperua) forest on white sand, 06°21'N; 58°50'W, 100 m, 22 Jan 1989, (old ♂ fl), *Hahn & Tiwari 5136* (US!). **Essequibo**: Basin of Issororo River, 1888, (♂ fl), *Jenman 5199* (K!); Essequibo River, Moraballi Creek, near Bartica, near sea level, 6 Nov 1929, (♂ fl), *Sandwith 561* (K! [3], NY n.v.).

**SURINAME.** Without locality, Nov 1941, (st), *Krukoff 12305* (GH!, NY n.v.); Railroad Paramaribo-Dam, Nov 1941, (st), *Krukoff 12335* (GH! [2], NY n.v., US n.v.); In hellingbos tussen, km 11.0 en 11.1, montibus, qui dicuntur Nassau, 17 Mar 1949, (st), *Lanjou & Lindeman 2775a* (NY n.v., U!); ibid., (st), *Lanjouw & Lindeman 2779* (NY n.v., U!); Nickerie, area of Kabalebo Dam Project, 30–130 m, Boegroemaka forest on gentle slope towards creek, west of road, km 80, 04°00'N; 57°30'W, 22 Sep 1980, (st), *Lindeman et al. 539* (U!); Lely Mts., SW plateaus covered by ferrobauxite, forest on plateau S of camp 4, 550–710 m, 5 Oct 1975, (st), *Lindeman et al. 805* (MO!, NY n.v.);

Open spot from fallen tree near 7100 m in line from road, km 80 eastward, area of Kabalebo Dam Project, 30–130 m, 11 Nov 1981, (st), *Lindeman & de Roon 808* (U!); Mapane Creek area, along Sarwa road, plot in succession, no date, (st), *Vreden 11663* (U!); Mapane Creek area, 29 Apr 1967, (st), *Vreden 11706* (U!); Without locality, (st), no date, *collector unknown* (U-32454-B!).

**VENEZUELA. Amazonas**: Atabapo, Rio Cunucunuma, entre las comunidades La Culebra y Huachamacari, entre El Cerro Duida y Huachamacari, 03°40'N; 065°45'W, 180-210 m, 28 Jan–8 Feb 1982, (♂ fl bud), *Steyermark et al. 125686* (MO!, NY!, US!).

### 3. Curarea crassa

Taxon classificationPlantaeRanunculalesMenispermaceae

Barneby

Figs 15 , 16

Curarea crassa Barneby, Brittonia 48: 20. 1996. Type: Brazil. Bahia: Uruçuca, 14°25'S; 39°01'W [39°03'W], 2 Dec 1994 (old fr), *Jardim et al. 351* (holotype: CEPEC n.v.; isotypes: K!, NY! [image seen].

#### Description.

**Figure 15.**
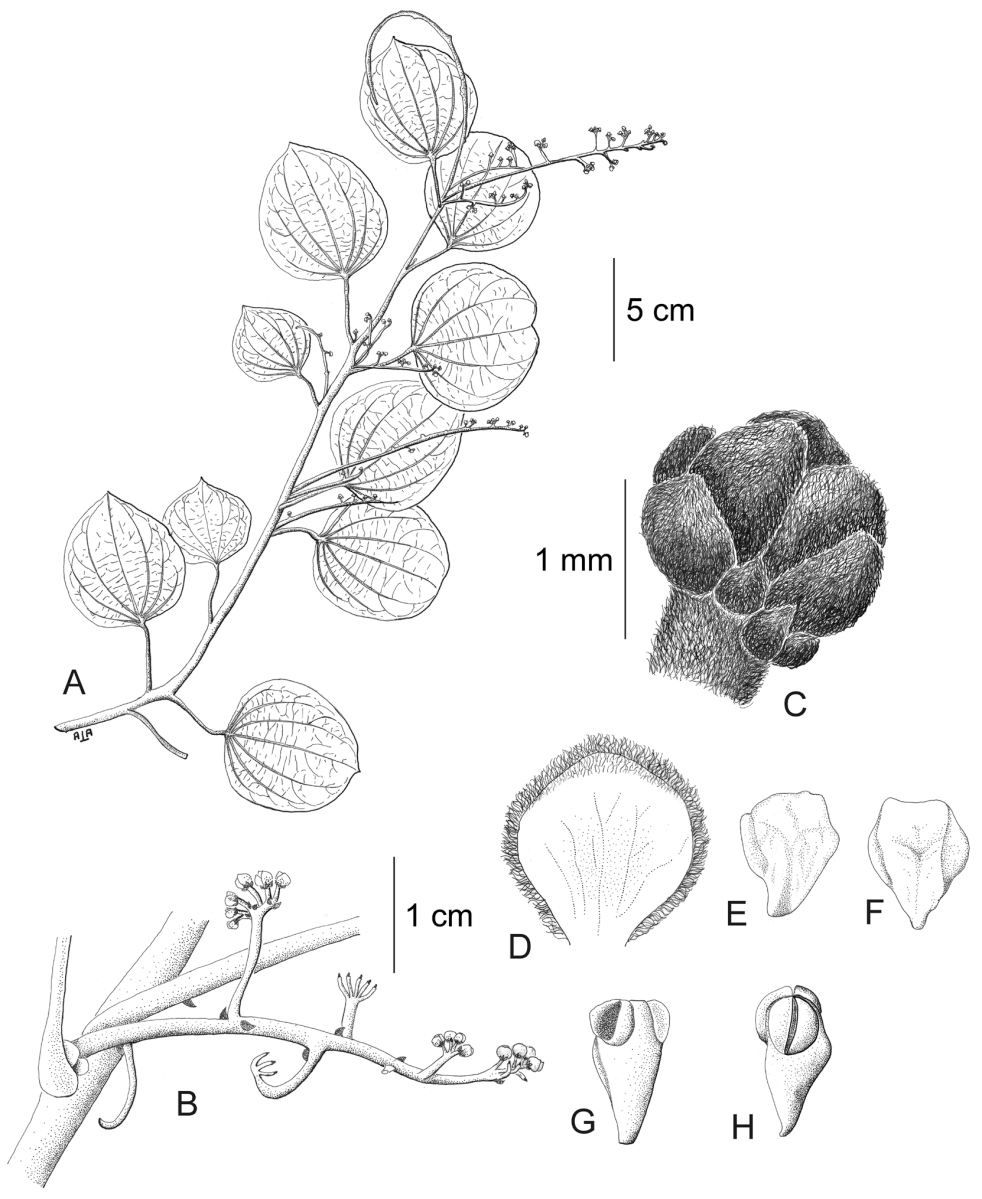
*Curarea crassa* staminate plant: **A** flowering branch **B** detail of inflorescence **C** flower bud **D** inner sepal, adaxial surface **E–F** outer and inner petals, abaxial views **G–H** outer stamens, abaxial-lateral surfaces (based on *Thomas et al. 10900*).

Large canopy *lianas* ca. 24 m tall; older stems with the lower part strongly flattened (width unknown); bark greyish to dark brown; branchlets dense and coarsely golden to silvery villose. *Leaves*: blades 8–10 × 6–9 cm, broadly elliptic to suborbicular, (narrowly ovate), coriaceous when mature or when directly exposed to sunlight in the canopy; surfaces discolorous, lustrous and glabrous adaxially, but sometimes sparsely tomentellous on main veins, golden or cream villous abaxially, the indumentum concealing the epidermis at all stages, base truncate to widely obtuse, margin entire, apex acuminate (especially when juvenile or not directly exposed to sunlight) to acute or retuse when mature or exposed to direct sunlight up in the canopy, 5–7 palmati- or plinerved, innermost pair of main veins acrodromous perfect on mature leaves, acrodromous imperfect on juvenile ones, secondary veins 0–2 pairs, arising almost at the apex of the blade, all veins slightly immersed adaxially, raised abaxially, but concealed by the indumentum; petioles 2.7–4.5 cm long, ridged, sparsely to densely golden or silvery villous, apical pulvinus conspicuous, rugulose, slightly flat to shallowly grooved or rounded adaxially. *Staminate inflorescences* solitary or fascicled, axillary or supra-axillary, thyrsi (Fig. 15A–B), golden villous tomentose; axes 9.8 cm long; primary branches compact and up to 1.1 cm long, with reduced (0–1) branching orders; bracts ca. 0.8 mm long, ovate, concave, fleshy, glabrous adaxially, golden villose abaxially.

**Figure 16.**
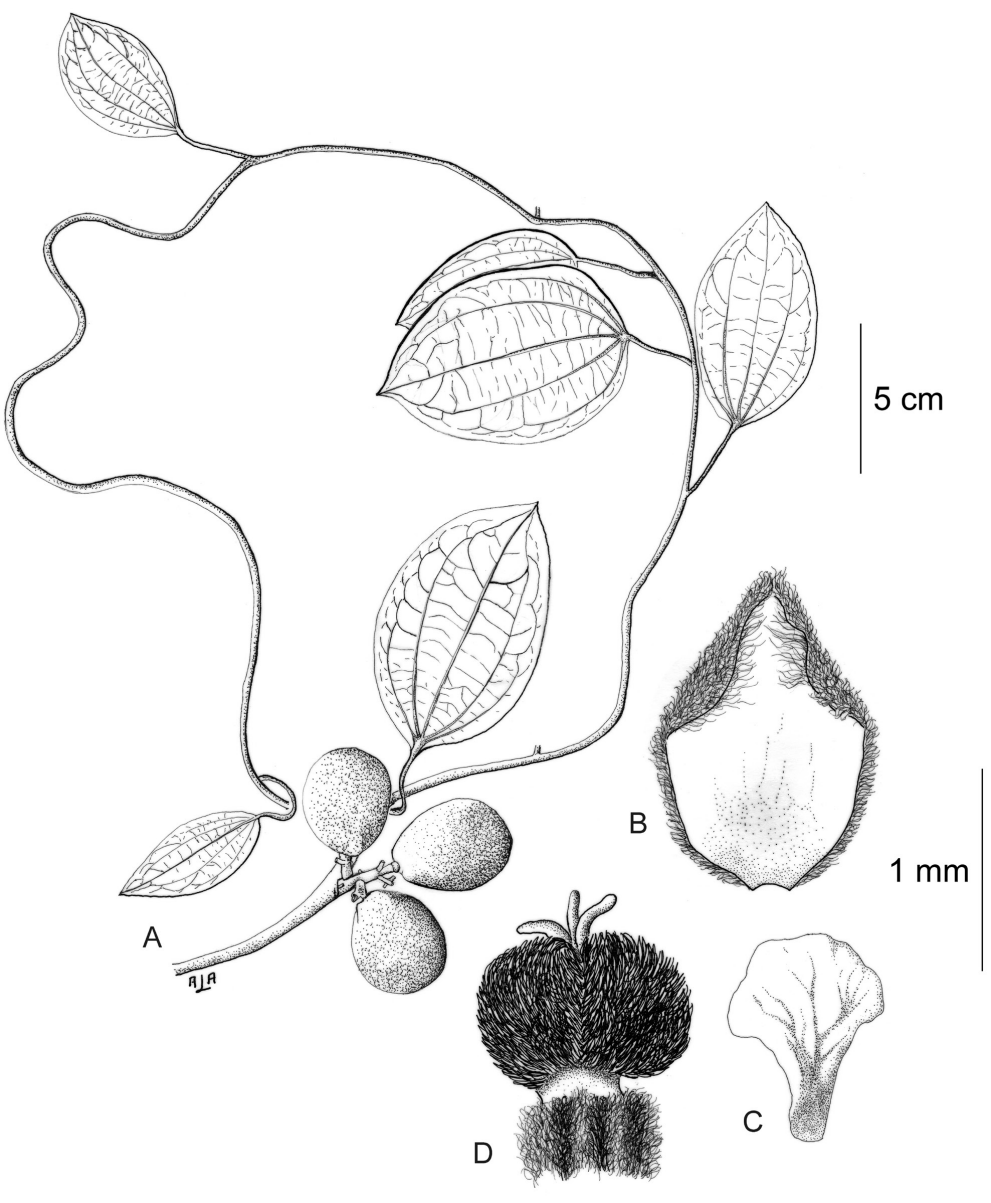
*Curarea crassa* pistillate plant: **A** fruiting branch **B** innermost sepal, adaxial surface **C** petal, abaxial surface **D** carpels (based on *Jardim et al. 609*).

*Pistillate inflorescences* (old) solitary or fascicled, axillary, few-flowered thyrsi, indumentum as on staminate inflorescences; axes ca. 3.8 cm long; bracts not seen, primary branches 0.6 cm long, indumentum as on the axis. *Staminate flowers* ca. 1.4 mm long, pale cream; pedicels ca. 1.3 mm long, terete, indumentum as on the axis; bracteoles 2, ca. 0.5 × 0.3 mm, narrowly ovate, fleshy, glabrous adaxially, light golden tomentose abaxially; sepals 6, indumentum as on bracteoles; outer sepals ca. 0.8 × 0.5 mm, ovate-lanceolate to ovate, base truncate, apex acute to obtuse; inner sepals ca. 1.4 × 1.3 mm, obovate or rhombic, base and apex obtuse, tip of inner sepals erect to hardly inflexed past anthesis; petals 6, ca. 0.7–0.8 × 0.6 mm, obovate, weakly concave, membranous, glabrous adaxially and abaxially, base cuneate, lateral margins inflexed, partially clasping the filaments, apex truncate or weakly retuse; stamens 6; filaments ca. 0.5 mm long, clavate, thick, free, glabrous adaxially and abaxially; anthers ca. 0.3 mm long, erect, connective thicker adaxially and forming a protruding keel at the base of the anthers (Fig. 15H), not overgrowing the thecae when older. *Pistillate flowers* ca. 2.1 mm long, greenish; pedicels ca. 0.5 mm long, appearing ridged, indumentum as on staminate inflorescences; bracteoles 2, 0.7 × 0.4 mm, ovate-lanceolate, fleshy, glabrous adaxially, light golden tomentose abaxially; sepals 9, in three whorls, glabrous adaxially, light golden tomentose abaxially; outer sepals ca. 0.9 × 0.5 mm, ovate-lanceolate; middle sepals ca. 1.6 × 0.7 mm, lanceolate to ovate-lanceolate; inner sepals 2.1 × 1.2 mm, elliptic or rhombic, their tips erect or weakly reflexed past anthesis; petals 3, ca. 1.3 × 1 mm, spatulate, weakly concave, membranous, glabrous adaxially, sparsely to densely silvery tomentellous abaxially, base clawed, apex truncate to slightly retuse; carpels 3, 1.1 × 0.7 mm, villous; style 0.4 mm long. *Infructescences* axes ca. 0.5–1.2 × 0.4–0.6 cm, indumentum moderate greyish-silvery tomentose; fruiting pedicels 5.8–6.1 mm long, terete; carpophores subglobose, ca. 2.4 mm long, convex at apex, golden villous. *Drupelets* 3.5 × 2.9 cm, (colour when ripe unknown), broadly obovoid (Fig. 16A), centrically or only weakly eccentrically attached, base oblique, stylar scar inconspicuous, surface golden villous; exocarp 6.7 mm thick, outer surface rugose, granular when dried; mesocarp not seen, but likely thin and mucilaginous; endocarp ca. 2.6 × 2.2 cm, papyraceous to chartaceous, surface smooth. *Seeds* and embryo not seen, (horny, ca. 23 × 18 mm, Barneby 1996).

#### Distribution and ecology.

*Curarea crassa* is known only from the coastal Atlantic Forest of Bahia in Brazil (Fig. 9), at ca. 76 m in elevation. Staminate flowering specimens were collected in May, whereas a pistillate flowering specimen was collected in February and immature fruiting specimens were collected in July and December.

#### Common names and uses.

**Brazil**: “buta” (*Fróes 12701/67*, st).

#### Etymology.

Not explained in protologue, but likely the name is in reference to its thick exocarp (mesocarp in original description).

#### Conservation status.

The five collections representing three localities resulted in an estimated EOO of 45 km^2^ and an AOO of 12 km^2^. The three localities correspond to two subpopulations and two locations, of which one is found within a protected area.

However, ongoing deforestation in the largely degraded Brazilian Atlantic forest may negatively affect the already localised subpopulations of *C. crassa*, further reducing suitable habitat. Based on these considerations, *C. crassa* is here assigned a preliminary status of “Endangered” [EN B1ab(i,ii,ii,iv,v) + B2ab(i,ii,ii,iv,v)].

#### Discussion.

*Curarea crassa* is vegetatively conspicuous because of its broadly elliptic or suborbicular leaves covered with a dense golden or creamy villous indumentum on the abaxial leaf blade surface. Similar indumentum may sometimes also be observed on the abaxial leaf blade surfaces of *C. barnebyana* and *C. tecunarum*; from *C. barnebyana*, it differs by its woolly, greyish-golden indumentum in the staminate inflorescences (*vs.* dark-brown). From *C. tecunarum*, *C. crassa* differs by its primary branches with few branching orders in the staminate inflorescence (*vs.* primary branches with several branching orders in *C. tecunarum*). Also, the large and broadly obovoid drupelets are unique to *C. crassa*.

The thick layer, from which the specific epithet is likely derived, was described as the mesocarp (Barneby 1996), however it is likely that the mesocarp in *C. crassa*, as in *C. barnebyana*, *C. tecunarum* and *C. aff. iquitana*, is mucilaginous and no longer noticeable in dried fruits, as was the case reported for *C. tecunarum* (Barneby and Krukoff 1971).

On morphological grounds, *C. crassa* is placed with the species of Group I. Micro-morphological characters shared amongst species in Group I are summarised in Table 5. *Curarea crassa* shares its staminate inflorescences with condensed branch orders with species of that group. They differ in the inflorescence indumentum colour, this being cream or golden in *C. crassa vs.* dark brown or greyish in *C. barnebyana*. The primary branches of the staminate inflorescences are also condensed in *C. candicans*, but there, trichomes are strigillose-tomentellous and not villous as in *C. crassa*.

In the protologue of *C. crassa*, Barneby (1996) described staminate flowers as having 9 sepals, but only 6 sepals were found in the only staminate plant available in this study (*Thomas et al. 10900*). This discrepancy is likely due to infraspecific variation as was also occasionally observed in *C. candicans* and in *C. aff. iquitana*.

**Table 5.** Morphological features of the two species groups within *Curarea*. * = species not studied for wood anatomy.

	Group I				Group II				
Macro- and micro-morphological Characters	*barnebyana*	*candicans*	crassa*	*tecunarum*	*cuatrecasasii*	Gentryana*	*iquitana*	*tomentocarpa*	toxicofera*
**Wood**									
rays in stem	relatively narrow	relatively narrow	n/a	relatively narrow	relatively narrow	n/a	wide	wide	n/a
average # of vascular bundles around pith	39	51	n/a	37	25	n/a	28	16	n/a
medullary sclerenchyma in pith	strong	strong	n/a	strong	weak	n/a	weak	weak	n/a
**Leaf**									
indumentum type on abaxial leaf blade surface	villous	web-like to tomentellous	villous	villous	strigillose-tomentellous	web-liketomentellous	tomentellous	tomentellous	strigillose-tomentellous
type of acrodromous venation	perfect	perfect	perfect	perfect	imperfect	imperfect	imperfect	imperfect	imperfect
veinlets	thick	thick	thick	thick	thin	thin	thin	thin	thin
mesophyll palisade cell arrangement	compact	compact	compact	compact	loose	loose	compact	loose	loose
mesophyll palisade size (mm)	23 × 5	24 × 6	28 × 3	23 × 6	14 × 7	14 × 7	15 × 6	14 × 5	19 × 5
**Reproductive**									
staminate inflorescence type of higher branching order	condensed	condensed	condensed	loose	loose	loose	loose	loose	loose
staminate inflorescence branching order	few	few	few	several	several	several	several	several	several
carpophore	subglobose	discoid	subglobose	subglobose	elongate	elongate	elongate	elongate	elongate

#### Specimens examined.

**BRAZIL. Bahia**: Uruçuca, Serra Grande, 7.3 km na Estrada Serra Grande/Itacaré, Fazenda Lagoa do Conjunto Fazenda Santa Cruz, 14°25'S; 39°01'W [39°03'W], 19 Jul 1994, (♂ fl), *Carvalho et al. 4563* (CEPEC n.v., NY! [image seen]; Região da Mata Atlântica, 14 Jul 1995, (imm fr), *Carvalho et al. 6031* (NY!); Una, Reserva Biológica do Mico-Leão (IBAMA), entrada no km 46 da rod, BA-001, Ilhéus/Una, Região da Mata Higrófila Sul Bahiana, 8–10 km na Estrada que margeia ao Rio Maruim, 15°09'S; 39°05W, 6 Jun 1996, (imm fr), *Carvalho et al. 6222* (CEPEC n.v., NY! [image seen]); Ilhéus, basin of Rio Santa Ana, high forest, near Bom Gosto, 8 Dec 1942 (st), *Fróes 12701/67* (A!, NY n.v.); Uruçuca, Serra Grande, 7 .3 km na estrada Serra Grande/Itacaré, Fazenda Lagoa do Conjunto Fazenda Santa Cruz, 14°25'S; 39°01'W [39°03'W], 9 Feb 1995, (♀ fl), *Jardim et al. 609* (NY!); 7.4 km north of Serra Grande on road to Itacaré, 14°25'24"S; 039°03'38"W, 12 May 1995, (♂ fl), *Thomas et al. 10900* (MO!, NY!).

### 4. Curarea cuatrecasasii

Taxon classificationPlantaeRanunculalesMenispermaceae

Barneby & Krukoff

Figs 17 , 18

Curarea cuatrecasasii Barneby & Krukoff, Mem. New York Bot. Gard. 22(2): 14. 1971. Type: Colombia. Antioquia: Rain forest of Villa Agraria, Las Caucheras, basin of Río León o Bacubá, 95 m, 2 Oct 1961, ([imm?] fr), *Cuatrecasas & Willard 26168* (holotype: COL!, NY neg. 8433; isotypes: NY! [NY00008325, frag.], US! [US00104020]).

#### Description.

**Figure 17.**
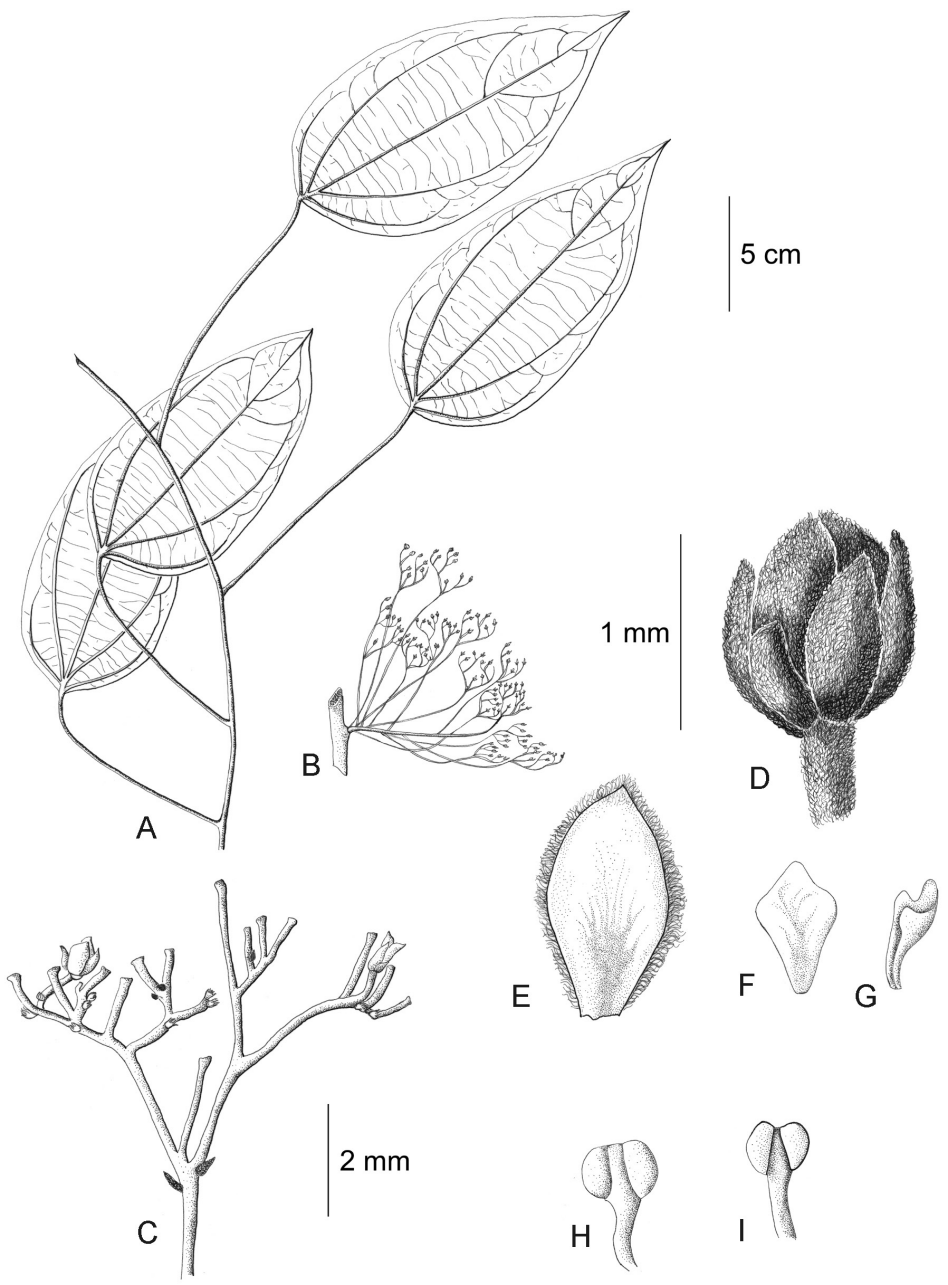
*Curarea cuatrecasasii* staminate plant: **A** habit **B** inflorescence **C** inflorescence detail **D** flower bud **E** inner sepal, adaxial surface **F–G** outer and inner petals, abaxial and lateral surfaces **H–I** outer stamens, adaxial and abaxial surfaces (based on *Aguilar 3303*).

Medium-sized understory *lianas* about 5–10 m tall; older stem more or less terete to weakly irregularly flattened, 0.5–1.5 cm wide; bark dark brown, with shallow lengthwise fissures and conspicuously tuberculate-lenticellate; branchlets densely brownish to silvery strigillose-tomentellous to glabrate. *Leaves*: blades 9–26 × 4.3–13.5 cm, ovate to elliptic, chartaceous at all stages, base obtuse to rounded, apex acuminate, cuspidate when juvenile; surfaces discolorous, lustrous and glabrous adaxially, indumentum finely silvery strigillose-tomentellous abaxially, rarely confined to the areoles with age; 3(5) palmati- or plinerved, innermost pair of main veins acrodromous imperfect at all stages, midrib and lateral nerves slightly raised above, conspicuously raised abaxially, secondary veins 2(3) pairs, veinlets slightly prominent adaxially in both juvenile and mature leaves, raised abaxially; petioles (2.9–)6.2–18 cm long, ridged, rugulose, brownish or silvey strigillose-tomentellous to glabrate, distal pulvinus rugulose. *Staminate inflorescences* fascicled, cauliflorous, lax thyrsi (Fig. 17B–C), densely silvery or brownish strigillose-tomentellous; axes 2.2–8 cm long; primary branches 1.8–6.2 cm long, filiform, with several (2–)4–5 branching orders; bracts 0.4–1.1 mm long, lanceolate to narrow ovate, concave, fleshy, indumentum as on inflorescence.

**Figure 18.**
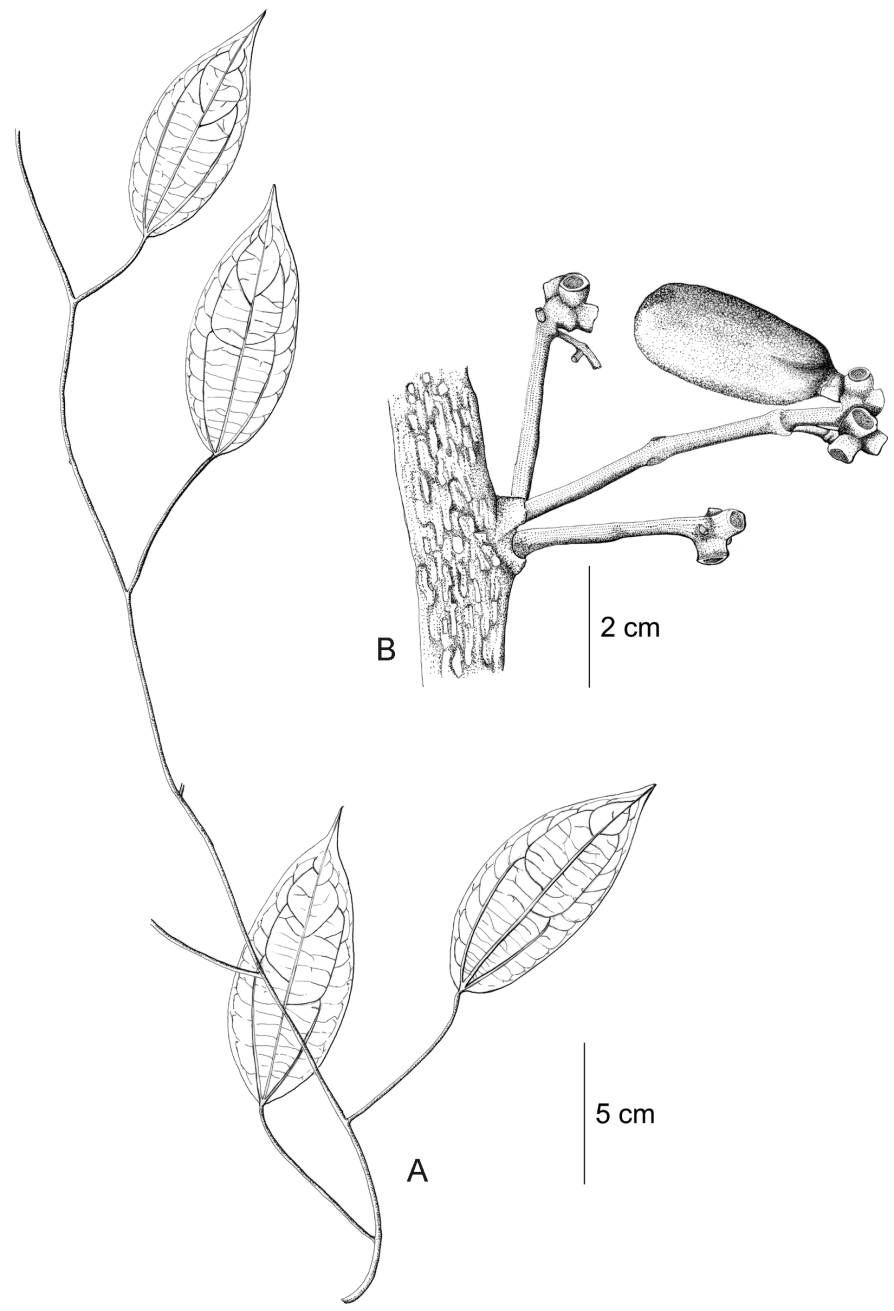
*Curarea cuatrecasasii* pistillate plant: **A** habit **B** infructescence (**A**, based on *Cuatrecasas & Willard 26168*; **B** based on *Marin 246*).

*Pistillate inflorescences* unknown. *Staminate flowers* 1.2–1.6 mm long, green, greenish or brownish; pedicels 0.4–2.8 mm long, terete, sometimes ridged, indumentum as on the axis; bracteoles 2–3, 0.2–0.6 × 0.1–0.4 mm, ovate-lanceolate, oblong or ovate-rhombic, fleshy, glabrous adaxially, light brown tomentellous abaxially; sepals 6, glabrous adaxially, light brown to silvery tomentellous abaxially; outer sepals 0.8–1 × 0.5–0.8 mm, ovate-lanceolate, ovate or obovate, base and apex obtuse; inner sepals 1.2–1.7 × 0.6–1.1 mm, obovate or elliptic, sometimes oblong or ovate-rhombic, base cuneate, apex acute to obtuse, tip of inner sepals erect to strongly reflexed past anthesis; petals (5)6, 0.5–0.9 × 0.2–0.5 mm, inner ones slightly longer, narrowly obovate-trilobed or spatulate, weakly concave, membranous, glabrous adaxially, glabrous to sparsely silvery tomentellous abaxially, weakly to strongly recurved above the cuneate base, lateral margins inflexed, partially clasping the filaments, apex acute, sometimes obtuse; stamens (3–)6; filaments 0.3–0.7 mm long, inner ones slightly longer clavate to clavate-sigmoid, moderately thick, free or connate at base, glabrous; anthers 0.2–0.3 mm long, erect, connective thicker adaxially and forming (or not) a protruding keel at the base of the anther, sometimes apically overgrowing thecae and forming a hump adaxially when older (Fig. 17H–I). *Pistillate flowers* unknown. *Infructescences* axes 1.3–4 × 0.2–0.7 cm, bark exfoliating, cauliflorous, lax thyrsi, with simple dichasia as primary branches, brownish strigillose-tomentellous; fruiting pedicels (1.2–)6.5 mm long, terete to weakly clavate; carpophores 2.4–4.5 mm long, elongate, weakly terete to claviform, truncate or convex at apex, velutinous. *Drupelets* 1.9–3.2 × 1.2–1.8 cm, yellow or pale orange when ripe, oblongoid or ellipsoid (Fig. 18B), (weakly reniform), weakly to strongly eccentrically attached, base truncate, obtuse to cuneate (gradually narrowed toward the base in a short (3–3.5 mm) stipe; stylar scar conspicuous; exocarp 1.2–1.9 mm thick, surface rugulose or muriculate, velutinous, granular when dried; mesocarp thin, mucilaginous; endocarp 1.5–2.4 × 0.9–1 cm, chartaceous, surface smooth. *Seeds* with embryo 3.8–4.8 cm long, crustaceous, cotyledons sometimes unequal.

#### Distribution and ecology.

From Costa Rica throughout Panama to northwestern Colombia (Fig. 9), at elevations of 10–650(–1100) m. In wet tropical lowland to pre-montane forests. Staminate flowering specimens were collected in March, April, May, June and September; pistillate flowering specimens are unknown; fruiting specimens were collected in January, March and July–December.

#### Common names and uses.

**Costa Rica**: edible fruits (*Morales et al. 3243*, mat fr). **Colombia**: used as magic plant by the Waunana (*Forero 666*, st).

#### Eponymy.

As per Barneby and Krukoff (1971), “the specific epithet honors Dr. José Cuatrecasas, who contributed greatly to the knowledge of the Colombian Flora and was a co-collector of the holotype”.

#### Conservation status.

The calculated Extent of Occurrence (EOO) based on 26 collections representing 25 localities is 184,445 km^2^, whereas the Area of Occupancy (AOO) is estimated as 100 km^2^. Of the 23 subpopulations, 12 occurred in protected areas in Panama and Costa Rica and, although the species is not abundant where it occurs, it has a broad distribution. Hence, *C. cuatrecasasii* is assigned a preliminary category of “Least Concern” (LC).

#### Discussion.

*Curarea cuatrecasasii* is distinguished from its congeners by the combination of slender staminate inflorescences bearing filiform primary branches, petals weakly to strongly recurved shortly above the base, connectives forming an adaxial hump at the apex of thecae when older and ovate or ovate-elliptic, 3–5-veined leaves with silvery strigillose-tomentellous indumentum on the abaxial surface. The staminate inflorescence of this species resembles those of *C. gentryana* and is discussed under the latter species.

In fruit, *C. cuatrecasasii* is indistinguishable from *C. iquitana*, *C. tomentocarpa* and *C. toxicofera* and they all share elongate carpophores, but they can be separated geographically: *C. cuatrecasasii* is restricted to the Pacific side of the Andes from North Eastern Colombia to Costa Rica and the other species are known only from the eastern side of the Andes in Colombia, Ecuador, Peru, Brazil and Bolivia.

The vessels width of *C. cuatrecasasii* is like that of *C. tecunarum* and *C. barnebyana* in being, on average, larger than the remaining species in group II (Table 5).

#### Selected specimens examined.

**COSTA RICA. Puntarenas**: Reserva Forestal Golfo Dulce, Aguabuena, Sector Norte, [08°42'20’’N; 083°28'30’’W], 50–150 m, 21 Nov 1991, (imm fr), *Aguilar 682* (MO!); Parque Nacional Corcovado, Sirena, Corcovado basin trail, [08°29'N; 083°35'W], 50 m, 30 May 1989 (♂ fl), *Kernan & Phillips 1147* (CR!, F [2]!, INB!, MO!). **San José**: Puriscal, Z.P. La Cangreja, faja Costeña del Valle de Parrita, Mastatal de Puriscal, bosque primario en la cuenca del Río Negro, por La Ceiba, 09°41'24"N; 084°23'24"W, 300 m, 25 Nov 1994, (mat fr), *Morales et al. 3243* (CR!, MO!).

**PANAMA. Canal Zone**: Along Rio Mendoza and small tributary, 1/2-1 km upstream from Pipeline Road bridge, 8 km NW of Gamboa, Premontane wet forest, 100 m, 1 Nov 1973, (imm fr), *Nee 7731* (COL!, F n.v., MO-2035834, US!. **Coclé**: Along Llano Grande to Coclesito road above Cascajal, near divide, forest, 08°42'N; 080°28'W, 500 m, 11 Jan 1986, (imm fr), *McPherson 7956* (MO!). **Colón**: Rio Guanche, ca. 2.5 km upriver from bridge on road to Portobelo, [09°30'N; 079°39'W], 10–100 m, 14 Dec 1974, (imm fr), *Mori & Kallunki 3695* (MO!). **Darien**: Area from below the Rancho Frio to near ridgetop of Pirre Chain, [07°49'N; 077°43'W], 600–1100 m, 15 Nov 1977, (imm fr), *Folsom et al. 6357* (MO!, NY!). **Panama**: Pipeline road, premontane wet forest, [09°14'42N; 079°48'53"W–09°07'26"N; 079°42'33"W], 50–120 m, 11 Mar 1983, (♂ fl), *Gentry & Hamilton 41126* (MO!); Primary forest, along road between El Llano and Carti-Tupile road, from 12 miles above Pan American Hwy to continental Divide, [09°18'N; 078°56'W–09°19'N; 078°57'W], 200–500 m, 30 Mar 1973, (imm fr), *Liesner 1325* (MO-2035837); Pipeline Road, ca. 12 km NW of Gamboa, Tropical wet forest, [09°10'N; 079°45'W], 100 m, 26 Aug 1975, (imm fr), *Mori 7946* (MO!). **San Blas**: Quebrada E of town of Puerto Obaldia, upriver from the dam (represa), [08°40'N; 077°24'W], 0–50 m, 18 Apr 1982, (♂ fl), *Knapp & Mallet 4706* (MO!).

**COLOMBIA. Antioquia**: Mpio. Taraza, corregimiento “El Doce”, Hacienda Las Mercedes, La Quebradona, 200 m NE de Medellin, 650 m, 3 Jul 1980, (imm fr), *Callejas 1183* (NY!); Mpio. Chigorodó, vereda Bohios, Finca La Cabaña, 30 m, 4 Apr 1985, (♂ fl.), *Renteria 3754* (JAUM n.v., MO!). **Bolivar**: Mpio. Achi, Inspección de La Raya, 100 m, 6 May 1987, (♂ fl), *Cuadros & Gentry 3606*, (JBGP n.v., MO!, NY!). **Chocó**: Mpio. de Riosucio, Zona de Urabá, Cerros del Cuchillo, Sector Cuchillo Negro, 10–30 m, 7 Sep 1987, (imm fr), *Cárdenas 374*, (JAUM n.v., MO!); Mpio. Bahia Solano, Corregimiento El Valle, trocha El Valle-Boro Boro, 06°21'N; 076°26'W, 17 Apr 1989, (♂ fl), *Espina et al. 2645* (MO!).

### 5. Curarea gentryana

Taxon classificationPlantaeRanunculalesMenispermaceae

R.Ortiz sp. nov.

urn:lsid:ipni.org:names:77185799-1

Fig. 19

#### Diagnosis.

The species is distinguished from its congeners by its staminate flowers with the lateral margins of inner petals adaxially connate or connivent, also by its large broadly obovoid or ellipsoid drupelet that has a silvery tomentellous indumentum.

#### Type.

Ecuador. Esmeraldas: San Lorenzo Canton, Reserva Indígena Awá, Parroquia Ricaurte, Comunidad Balsareño, Río Palabí, bosque muy húmedo tropical, bosque primario, disturbado, 01°09'N; 078°31'W, 100 m, 15–29 Apr 1991, (♂ fl), *Rubio & Quelal 1503* (holotype: MO!; isotypes: NY!, QCNE!).

#### Note.

The earlier listing of the name in Ortiz-Gentry (2000) does not constitute effective publication as per article 30.8 of the Melbourne Code (MacNeill et al. 2012), and is therefore here being validated.

#### Description.

**Figure 19.**
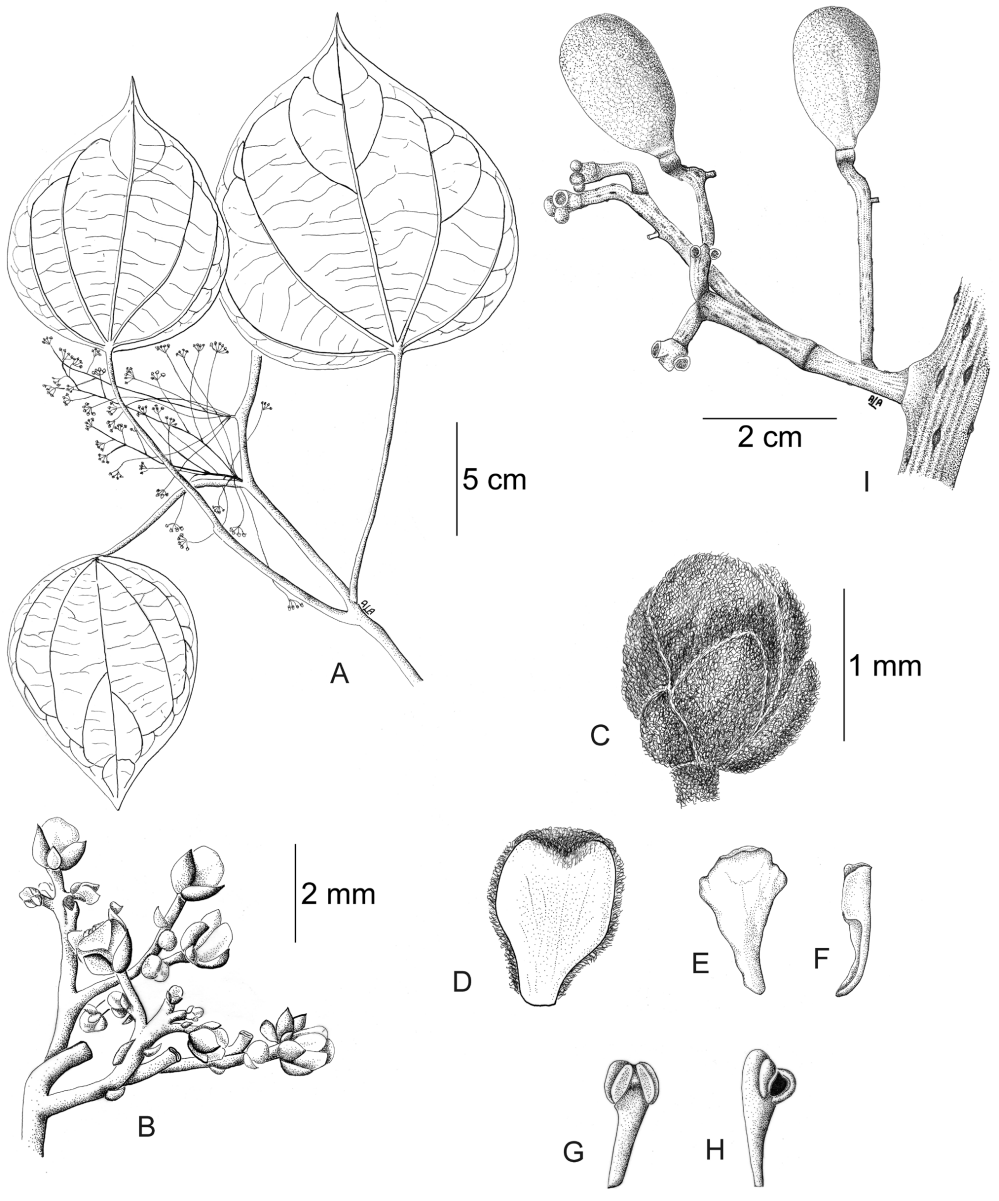
*Curarea gentryana* staminate plant: **A** flowering branch **B** detail of inflorescence **C** flower bud **D** inner sepal, adaxial surface **E–F** outer and inner petals, abaxial and latero-adaxial surfaces **G–H** outer stamens, abaxial and lateral views **I** pistillate plant, infructescence (**A–H** based on *Rubio & Quelal 1503* **I** based on *Aulestia et al. 9*).

Medium-sized understory *lianas* ca. 8 m tall, older stems more or less terete, 0.5–1 cm diameter, bark dark brown, with shallow lengthwise fissures and conspicuously tuberculate-lenticellate; branchlets brownish strigillose-tomentellous to glabrate. *Leaves*: blades 17–22 × 14–23 cm; broadly ovate, base truncate or weakly cordate, apex acuminate, cuspidate when juvenile; chartaceous when mature or when exposed to direct sunlight, otherwise membranous; surfaces discolorous, lustrous and glabrous adaxially, finely silvery web-like abaxially, indumentum concealing the surface, persistent at all stages; 5-palmatinerved, innermost pair of main veins acrodromous imperfect at all stages, midrib and lateral nerves slightly raised to weakly sunken adaxially, conspicuously raised abaxially, secondary veins 2(3) pairs, arising above the middle of the blade, veinlets raised on both surfaces; petioles 11.3–16.9 cm long, ridged, silvery or brownish strigillose-tomentellous to glabrate, weakly pulvinate at both ends, apical one conspicuous, rugulose. *Staminate inflorescences* fascicled, axillary or cauliflorous, lax thyrsi (Fig. 19A–B), silvery or brownish strigillose-tomentellous to glabrate; axes ca. 7.6 cm long; primary branches ca. 4 cm long, with several (2–5) orders of cymose branching, less frequently the higher branch orders are reduced on alternating sides, appearing racemiform; bracts ca. 1.1 mm long, lanceolate, concave, fleshy, indumentum as on inflorescence. *Pistillate inflorescences* unknown. *Staminate flowers* ca. 1.3 mm long, cream; pedicels ca. 0.7 mm long, terete, indumentum as on inflorescence; bracteoles 1–2, ca. 0.2 × 0.2 mm, ovate or oblong, fleshy, glabrous adaxially, silvery tomentellous abaxially; sepals 6, glabrous adaxially, silvery tomentellous abaxially; outer sepals ca. 1 × 0.6 mm, narrowly ovate or ovate-rhombic, base obtuse to truncate, apex acute or obtuse; inner sepals ca. 1.6 × 0.9 mm, obovate or weakly rhombic, base obtuse to cuneate, apex acute to obtuse, tip of inner sepals erect to strongly reflexed at anthesis; petals 6, ca.0.8– 0.9 × 0.4–0.5 mm, narrowly obovate-trilobed to strongly spatulate, the latter the more so when there are only three stamens, moderately concave, membranous, glabrous adaxially, glabrous or sparsely silvery tomentellous abaxially, base cuneate or strongly clawed, lateral margins strongly inflexed, partially clasping the filaments and sometimes the inner ones are adaxially connate or coherent, when 3 stamens are present, apex rounded to slightly retuse or truncate; stamens 3(–6), 1(2)-whorled, filaments ca. 0.6 mm long, clavate, free (or variously connate when 4–6), glabrous; anthers ca. 0.3 mm long, erect, connective adaxially thicker, sometimes forming a keel at the base of the anthers, apically overgrowing thecae and forming a hump when older (Fig. 19H). *Pistillate flowers* unknown. *Infructescences* axes ca. (1.3)3.5 × 0.3–0.4 cm, conspicuously lenticellate, strigillose-tomentellous or glabrate; fruiting pedicels (in immature fruits) 4.7–6.3 mm long, terete; carpophores 2.5–3.1 mm long, terete in immature fruits, not seen in mature fruits, truncate at apex, velutinous. *Drupelets* 3.6–4.6 × 2.1–2.8 cm, (colour when ripe unknown), weakly obovoid or ellipsoid (Fig. 19I), weakly eccentrically attached, base obtuse, stylar scar not apparent; exocarp ca. 2.6 mm thick, surface rugose, sparsely brownish velutinous tomentellous or glabrate, granular when dried; mesocarp thin and mucilaginous; endocarp ca. 3.7 × 2.2 cm, papyraceous, surface smooth. *Seeds* with embryo ca. 8 cm long, cotyledons unequal.

#### Distribution and ecology.

*Curarea gentryana* is known only from northwestern Ecuador (Fig. 9), at elevations of 80–225 m. It is found along creek margins in lowland tropical wet forest. Flowering specimens were collected in April and fruiting specimens were found in February, April and July.

#### Common names and uses. Ecuador.

“granadilla” (*Rubio & Quelal 1434*, imm fr).

#### Eponymy.

The specific epithet honours the late Dr. Alwyn H. Gentry, a dedicated and extraordinary botanist and inspiring mentor, who died tragically in a plane crash in August 1993 while surveying a dry forest reserve in western Ecuador.

#### Conservation status.

The species is known only from four collections from three localities from northwestern Ecuador. Assessment based on these collections resulted in an Extent of Occurrence (EOO) of 92.6 km^2^ and an Area of Occupancy (AOO) of 12 km^2^. The three localities represent three subpopulations, each found in a small communal protected area (Reserva Etnica Awá), the surrounding area of which has been subject to increased land conversion. Therefore, it is expected that the species will in the future be negatively affected by loss of its habitat quality that may lead to reduction in the population and potentially threatening its survival. Thus, *C. gentryana* is assigned a preliminary status of Endangered [EN, B1ab(i,ii,iii,iv,v) + B2ab(ii,iii,iv,v)].

#### Discussion.

The velutinous tomentellous indumentum covering the large obovoid or ellipsoid drupelets and the web-like indumentum on the abaxial surface of the broadly ovate leaves are unique to *Curarea gentryana*. The staminate inflorescences of *C. gentryana* somewhat resemble those of *C. cuatrecasasii*. However, *C. gentryana* has the spatulate inner petals with the lateral margins adaxially connate; spatulate inner petals may also be found in *C. cuatrecasasii*, but the lateral margins are not adaxially connate.

#### Paratypes.

**ECUADOR. Esmeraldas**: San Lorenzo Canton, Reserva Indígena Awá, Parroquia Ricaurte, Centro Guadualito, 01°15'N; 078°40W, 80 m, 20–29 Apr 1992, (fr), *Aulestia et al. 9* (MO!, QCNE!); Creek on left side of Rio Palaví, going up river, 2 bends up from Awá encampment past first island, 01°07N; 078°37'W, 225 m, 14 Feb 1988, (imm fr), *Hoover et al. 4530* (MO!); Comunidad Balsareño, Río Palabí, 01°09'N; 078°31W, 100 m, 15–29 Apr 1991, (fr), *Rubio & Quelal 1434* (MO!, QCNE!).

### 6. Curarea iquitana

Taxon classificationPlantaeRanunculalesMenispermaceae

(Diels) R.Ortiz comb. nov.

urn:lsid:ipni.org:names:77185800-1

Fig. 20

Chondrodendron iquitanum Diels, in Mildbr., Notizbl. Bot. Gart. Berlin 9: 997. 1926. Type: Peru. “Oberes Marañon-Gebit. Mündung des Santiago”, 160 m, [3] Oct 1924, (♂ fl), *Tessmann 4196* (lectotype designation effected by Krukoff and Moldenke 1938, pg. 24: B!, photographs at MO!, NY!; isolectotypes: G! [G (2), F neg. 27514], NY! [NY00008329, frag.]).

#### Description.

Medium-sized understory *lianas*, (0.5–)2–7 m tall; older stems more or less terete, up to 1.5 cm diameter, bark dark brown, with shallow lengthwise fissures and scarcely tuberculate-lenticellate; branchlets glabrescent. *Leaves*: blades (11–)14–37 × (5–)14 –30 cm; ovate to broadly ovate; chartaceous when mature or when exposed to direct sunlight, base obtuse, truncate or weakly cordate, apex acute, acuminate, to cuspidate; surfaces discolorous, lustrous and glabrous adaxially, finely silvery tomentellous abaxially, indumentum concealing the surface at all stages, 3–5 palmati- to shortly plinerved, innermost pair of main veins acrodromous imperfect at all stages, midrib and lateral nerves slightly raised to weakly sunken adaxially, conspicuously raised abaxially, secondary veins 3(4) pairs, arising above the middle of the blade, veinlets raised above and below; petioles (3.9–)12–29.2 cm long, ridged, brownish-greyish strigillose to glabrate, apical pulvinus conspicuous, rugulose, scarcely flattened adaxially. *Staminate inflorescences* solitary or fascicled, axillary or cauliflorous, thyrsi, brownish to silvery strigillose, indumentum adpressed; axes (2.2–)7–14 cm long; primary branches 1–6 cm long, with (1)4–5 orders of branching; bracts (0.2–)0.7–1.2 mm long, ovate, concave, ascending, fleshy, glabrous adaxially, brown villous tomentellous abaxially. *Pistillate inflorescences* basically a thyrse, sometimes the primary branches reduced to single flowers, hence appearing racemose, brownish to silvery strigillose; axes 0.6–3.8 cm long; bracts 0.6–1 mm long, ovate, rather fleshy, glabrous adaxially, brownish villous abaxially. *Staminate flowers* 1.3–1.8 mm long, whitish, greenish, green-yellowish or orangish; pedicels (0.6–)1.3–3.1 mm long, terete, relatively thick, indumentum as on the axis, but somewhat spreading; bracteoles 2–3, 0.23–0.4 mm long, ovate or oblong, fleshy, glabrous adaxially greyish or silver villous-tomentellous abaxially; sepals 6, indumentum as on bracteoles; outer sepals 0.6–1.3 × 0.4–0.7 mm, ovate or ovate-elliptic, base truncate, apex acute; inner sepals 1.2–2.2 × 1–1.5 mm, narrowly ovate, ovate, elliptic or weakly obovate, base obtuse or cuneate, apex acute to rounded, tip of inner sepals erect, sometimes reflexed past anthesis; petals 6, 0.6–1.2 × 0.3–0.8 mm, inner ones slightly smaller and narrower, obovate, obovate-trilobed or flabelliform, weakly to moderately concave, membranous, glabrous adaxially, sparsely silvery tomentellous abaxially, base cuneate or slightly clawed, lateral margins inflexed, partially clasping the filaments, apex acute, sometimes obtuse in the inner ones; stamens 6, filaments 0.4–0.8 mm long, inner ones slightly longer, clavate, free or connivent, less frequently connate ca. half their length, glabrous; anthers 0.2–0.3 mm long, erect, connective protruding adaxially and forming a hump or a horn apically or not, protrusion more conspicuous when older. *Pistillate flowers* (mostly old) 1.7–2.2 mm long, greenish; pedicels 1.3–5.1 mm long, terete, indumentum as on the axis; bracteoles 1–3(4), 0.2–0.6 × 0.3–0.6 mm, ovate or oblong, rather fleshy, glabrous adaxially, greyish or silvery villous tomentellous abaxially; sepals 6–9(12), weakly concave, moderately fleshy, in 2–3 whorls, glabrous adaxially, greyish or silvery villous tomentellous abaxially; outer sepals ca. 0.5–1.3 × 0.4–0.8 mm, ovate, base truncate, apex acute; middle sepals 0.6–2.2 × 0.6–1.4 mm, broadly ovate to obovate, base obtuse or cuneate, apex acute; inner sepals 1.6–2.2 × 1.3–1.7 mm, ovate, obovate or elliptic, base cuneate or clawed, apex obtuse or rounded, tips erect to weakly reflexed past anthesis; petals 3(4), 1.3–1.7 × 0.6–0.8 mm, spatulate or obovate-trilobed, weakly concave, membranous, glabrous adaxially, glabrous or sparsely silvery tomentellous abaxially, base clawed, apex obtuse; carpels 3, 0.6–0.9 × 0.4–0.6 mm, dark to light brown villose, trichomes appressed-ascending; style 0.6–1.1 mm long. *Infructescences* axes 1–6 × 0.3–0.9 cm long, at times moderately stout, brown to silvery-strigillose-tomentose or glabrous; fruiting pedicels 2.3–4.6 mm long, terete, sometimes rather thick; carpophores 4–8 mm long, clavate in mature fruits, truncate at apex, cream or brownish velutinous. *Drupelets* 1.8–2.8 × 1.2–1.6 cm, yellow when ripe, ellipsoid or weakly oblongoid, weakly eccentrically attached, base obtuse, stylar scar basal; exocarp 0.9–1.2 mm thick, surface rugulose, cream or silvery velutinous or glabrescent, granular when dried; mesocarp said to be “clear and slimy” (*Berlin 542*); endocarp 1.7–2.4 × 0.8–1.2 cm, chartaceous, surface reticulate by scarcely pronounced veins. *Seeds* with embryo 3.6–4.9 cm long, cotyledons unequal.

#### Distribution and ecology.

Known from the lowlands to mid elevations in eastern to central Peru (Fig. 21), including the departments of Amazonas, Loreto and Pasco, from elevations of 106 m in Loreto up to 1380 m in Pasco. Staminate plants were collected in January-February, July-August and November. Pistillate flowers were found in February, October and December, whereas fruiting specimens were found all year round.

**Figure 20.**
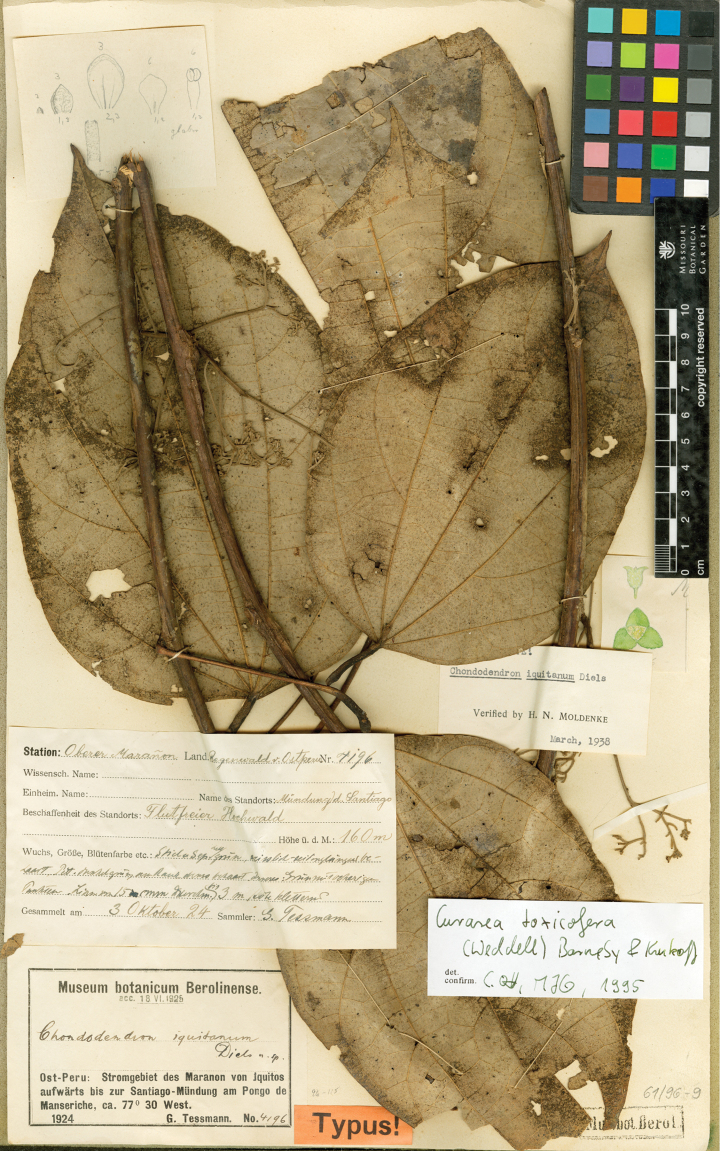
*Curarea iquitana* (photograph of the type of *Chondrodendron iquitanum*, *Tessmann 4196*, B).

**Figure 21.**
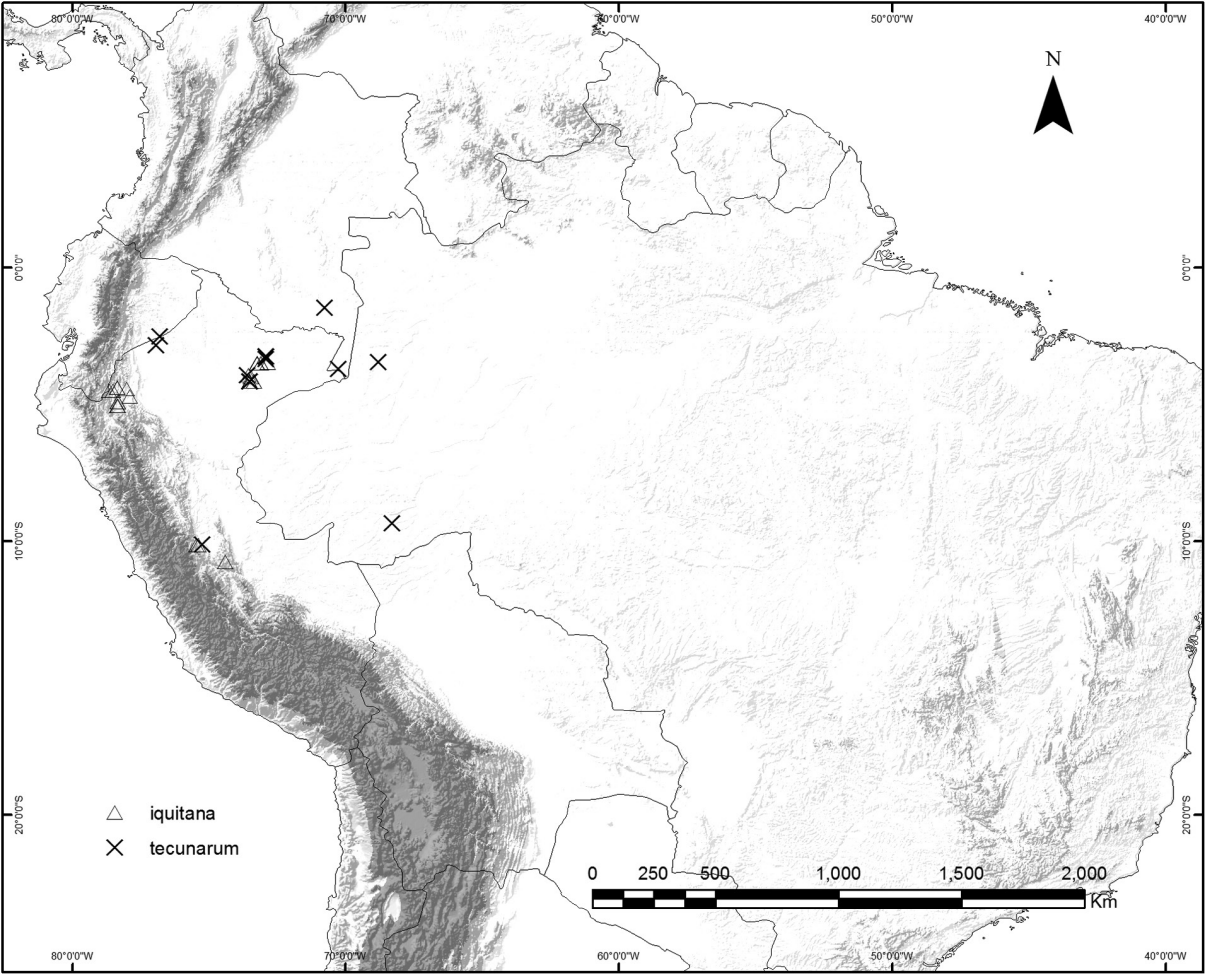
Geographic distribution of *Curarea iquitana* and *C. tecunarum*, for the latter selected specimens only, because most of the specimens are sterile.

#### Common names and uses.

**Peru**: “dabau” (*Ancuash 660*, ♂ fl, *Kayap 749*, imm fr, *1205*, mat fr); “dúpam” (*Berlin 542*, mat fr); “tsegásnum” (Aguaruna) (*Castro et al. 18929*, imm fr; *Lewis et al. 18475*, imm fr); “namaú” (*Kayap 156*, mat fr); “tseas daek” (*Kayap 1032*, imm fr); “tsegas” (*Kujikat 107*, imm fr); “tseusnum” (*Ancuash 1219*, ♂ fl); “ampihuasca amarilla” (*Schunke Vigo 2981*, mat fr); “ampihuasca delgadito” (*Schunke Vigo 6836*, ♂ fl); “ampihuasca negra” (*Schunke Vigo 7125*, ♂ fl).

#### Note.

Krukoff and Moldenke (1938) cited *Chondrodendron iquitanum* (*Curarea iquitana* in this study) as a “source of poison for darts of Indians” based on the label of the sterile *Mexia 6321a*. This specimen was later identified as *Curarea toxicofera* (Barneby and Krukoff 1971). I have not examined the referred specimen and hence I have not been able to confirm its identity.

#### Etymology.

Presumably in reference to Iquitos, the largest city in Peruvian Amazonia and in which general area the type specimen was surely collected.

#### Conservation status.

The assessment was based on 37 collections corresponding to 18 localities that yielded an Extent of Occurrence (EOO) of 279,551 km^2^ and an Area of Occupancy (AOO) of 72 km^2^. The 18 localities represent 17 subpopulations of which four occur within protected areas (Allpahuayo-Mishana National Reserve in the Iquitos area) and three are found on private lands. Although *Curarea iquitana* is not abundant where it occurs, it is however broadly distributed and hence it is assigned a preliminary status of “Least Concern” (LC).

#### Discussion.

*Curarea iquitana* is here resurrected from synonymy under *C. toxicofera*. *Curarea iquitana*, as defined here, encompasses a broad range of morphological variation and includes, at least provisionally, what is labelled the allpahuayo group (**al.**) in the linear discriminant analysis. While the iquitana (**iq**) and allpahuayo (**al.**) groups may represent different entities, at present it is premature to recognise them as different species, given that the morphological quantitative characters evaluated in this study partially or completely overlap between the two groups (Fig. 10B).

Additionally, features of pistillate flowers are still fragmentary or lacking, hence the extent of variation, if any, in these features, remains unknown. Collections from the foothills of central-eastern Peru in the Amazonas department from 200–800 m elevation closely resemble the type of *Chondrodendron iquitanum* Diels (*Tessmann 4196*), which was collected in the same general area – in the basin of the Marañon River, at 160 m in elevation – around which my concept of *Curarea iquitana* is centred. These collections tend to have conspicuously large, ovate or broadly ovate leaves with 5–7 main veins. The staminate inflorescences have a brownish to silvery strigillose indumentum and small flowers that range from 1.6 to 1.8 mm long, (mostly greyish villous and 1.3–1.8 mm long in the allpahuayo group). The adaxial horn-like protrusion of the connective at the apex of anthers, characteristic of *Curarea iquitana*, is variable amongst the studied collections, appearing as a horn (long and weakly incurved), as an apical-adaxial keel or as an adaxial hump, the latter more frequently being observed in the allpahuayo group; anthers are for the most part immersed in the connective. An old and fragmentary pistillate inflorescence of *C. iquitana* *s.s.* is a thyrse with brownish or silvery villous indumentum and the rugulose or muriculate drupelets have a golden-brownish velutinous-hispidulous indumentum. The only pistillate inflorescence known in the allpahuayo group has cymose primary branches proximally, distally these being reduced to single flowers giving the appearance of being racemose. The immature condition of the drupelets in most collections studied precludes their use in analyses of quantitative variation of the groups. Collections from non-flooded areas around Iquitos in eastern Peru, ca. 110–140 m in elevation and those from the Andean foothills in Pasco department, from 500–1380 m elevation, are here provisionally included in *C. iquitana*.

#### Selected specimens examined.

**PERU. Amazonas**: [Prov.] Condorcanqui, Río Cenepa, Camino Etseketal, norte de Huampami, monte, 600–800 ft, 31 Jul 1974, (♂ fl), *Ancuash 660* (MO!, NY!); Prov. Bagua, Distrito Imaza, Región Nororiental del Marañon, Comunidad Aguaruna de Kusú-Listra, Cerro Apág, margen derecha Quebrada Kusú, 05°02'24"S; 078°19'12"W, 600–700 m, 17 Sep 1996, (imm fr), *Díaz et al. 8239* (MO!); Rio Cenepa región, monte, orilla de Quebrada Huampami, 18 Jan 1973, (mat fr), *Kayap 156* (MO!); Río Cenepa, vicinity of Huampami, ca. 5 km east of Chávez Valdivia, Que Quebrada Najamtai entsa, en bosque primario, 04° 30'S; 78° 30'W, 200–250 m, 3 Aug 1978 (♀ fl & imm fr), *Kujikat 107* (MO!); Comunidad de Yamayakat, Río Marañon, bosque primario, 04°55'S; 078°19'W, 320 m, 15 Jul 1994, (♂ fl), *Vásquez et al. 18715* (MO!). **Loreto**: Prov. Maynas, Distrito de Indiana, campamento turístico de Explorama, bosque, 03°30'S; 073°14'W, 110 m, 10 Feb 1996, (♂ fl), *Ortiz et al. 167* (MO!); ibid., 11 Feb 1996, (mat. fr.), *Ortiz et al. 172* (MO!); Distrito de Iquitos, Estación Experimental de Allpahuayo, IIAP, bosque, 04°10'S; 073°30'W, 120–140 m, 20 Feb 1996, (♂ fl), *Ortiz 176* (MO!); ibid., 22 Feb 1996 (♂ fl), *Ortiz 181* (MO!); ibid., 23 Feb 1996, (♀ fl), *Ortiz 184* (MO!); ibid., 23 Feb 1986, (♀ fl, imm & mat fr), *Ortiz 186* (MO!). **Pasco**: Dist. Palcazu, Comunidad Nativa Alto Lagarto (Reserva Comunal Yanesha), remanente de bosque primario, 10°08'04"S; 075°22'06"W, 500 m, 9 Feb 2011, (mat fr), *Rojas & Ortiz 7674* (HOXA n.v., MO!, USM!); Prov. Oxapampa, Parque Nacional Yanachaga-Chemillen, sector Alto Lagarto, remanente de bosque primario, 10°07'44"S; 075°26'41"W, 1380 m, 20 Aug 2011, (imm fr), *Rojas et al. 7909* (HOXA n.v., MO!, USM!); Prov. Oxapampa, Gran Pajonal, trail between Chequitavo and Shumahuani, primary forest, 1200–1300 m, 10°45'S; 074°23'W, 30 Mar 1984, (mat fr), *Smith 6584* (MO!).

### 7. Curarea tecunarum

Taxon classificationPlantaeRanunculalesMenispermaceae

Barneby & Krukoff

Figs 22 , 23

Curarea tecunarum Barneby & Krukoff, Mem. New York Bot. Gard. 22(2): 12. 1971. Type: Brazil. Amazonas: Basin of Rio Solimões, Municipality São Paulo de Olivença, basin of creek Belem, 26 Oct–11 Dec 1936, (♂ fl), *Krukoff 8713* (holotype: NY!; isotypes: BM!, BR!, F!, MO!, U!, US!).

#### Description.

**Figure 22.**
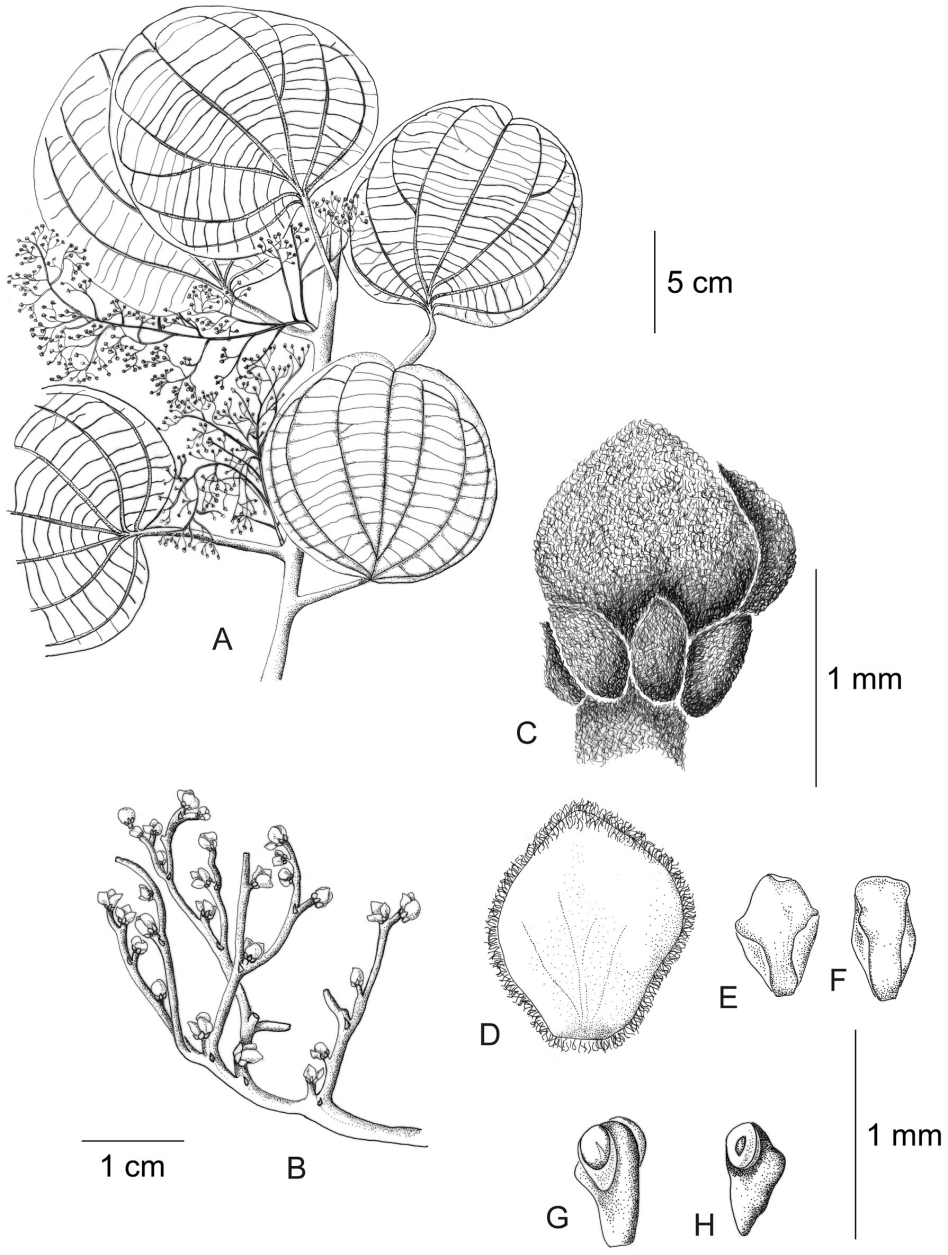
*Curarea tecunarum* staminate plant: **A** flowering branch **B** detail of inflorescence **C** flower bud **D** inner sepal, adaxial surface **E–F** outer and inner petals, adaxial surfaces **G–H** outer stamens, latero-abaxial and lateral surfaces (**A–B** based on *Krukoff 8713* **C–H** based on *Pipoly et al. 12846*).

**Figure 23.**
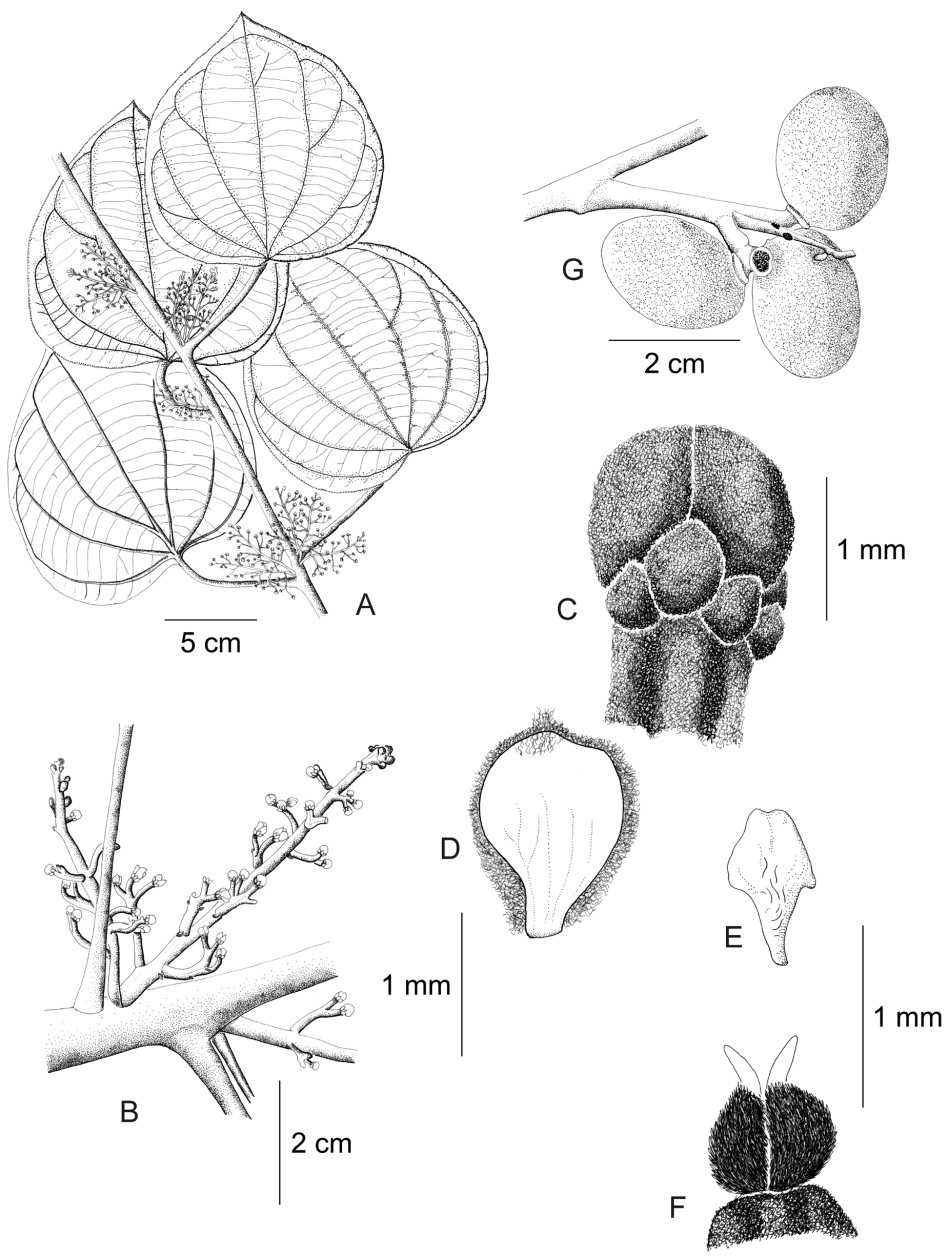
*Curarea tecunarum* pistillate plant: **A** flowering branch **B** detail of inflorescence **C** flower bud **D** innermost sepal, adaxial surface **E** petal, abaxial surface **F** carpels **G** infructescence (**A–F** based on *Fróes 21446* **G** based on *van der Werff & Vásquez 13909*).

Large canopy *lianas* about 20–30 m tall; older stems, 5–15 cm wide, strongly flattened; bark dark brown, with shallow lengthwise fissures; branchlets brown to creamy villous. *Leaves*: blades 9–30 × 7–20 cm, ovate to broadly ovate; chartaceous to subcoriaceous when mature or when directly exposed to sunlight, base truncate, obtuse or shallowly cordate, margin entire, apex acuminate to retuse (cleft in *Krukoff 8713*), cuspidate when juvenile; surfaces conspicuously discolorous, lustrous and glabrous adaxially, coarsely cream to silvery tomentulose abaxially, matted when older, indumentum concealing the abaxial surface at all stages, (5–)7–9 palmati- or plinerved, innermost pair of main veins acrodromous perfect on mature leaves, acrodromous imperfect on juvenile ones, midrib and lateral nerves slightly impressed adaxially, conspicuously raised abaxially, secondary veins 1–2 pairs, arising above the middle of the blade, sometimes absent, veinlets immersed adaxially, raised abaxially; petioles 4.7–21 cm long, smooth, densely creamy or brownish villous to glabrate, apical pulvinus more conspicuous, rugose, weakly flattened adaxially. *Staminate inflorescences* solitary or fasciculate, axillary or slightly supra-axillary, thyrsi (Fig. 22A–B), densely brownish or greyish tomentellous; axes 5.7–19 cm long; primary branches 1.1–3.5 cm long, lax, with several (3–6) cymose orders of branching, higher order branching frequently reduced; flowers frequently sessile at the centre of the irregularly further dichotomous branchings; bracts 0.7–1.1 mm long, narrowly ovate, concave, fleshy, glabrous adaxially, brown tomentellous abaxially. *Pistillate inflorescences* solitary or fascicled, axillary stout thyrsi (Fig. 23B), with dichasial primary branches or these less frequently reduced to single flowers and hence appearing racemiform, densely light brown or greyish tomentellous; axes ca. 5 cm long; bracts ca. 2 mm long, ovate, concave, fleshy, indumentum as on inflorescence. *Staminate flowers*, 1.2–1.7 mm long, green; pedicels (0–)0.3–3.3 mm long, thick, terete, indumentum as on staminate inflorescence; bracteoles 1–2, 0.2–0.4 × 0.1–0.3 mm, narrowly ovate, fleshy, glabrous adaxially, light brown or creamy tomentellous abaxially; sepals 6; outer sepals 0.4–0.9 × 0.3–0.7 mm, ovate or oblong, indumentum as on bracteoles, base truncate, apex acute; inner sepals 1.1–1.9 × 1–1.7 mm, obovate or ovate-rhombic, mostly light brown or greyish tomentellous abaxially, obtuse at base, apex obtuse or rounded, tip of inner sepals erect or spreading, less frequently reflexed past anthesis; petals 6, 0.4–0.8 × 0.2–0.6 mm, inner ones slightly shorter and narrower, obovate-trilobed or rhombic, weakly concave, membranous, glabrous adaxially and abaxially, base cuneate-truncate, lateral margins inflexed, partially clasping the filaments, apex obtuse, truncate or retuse; stamens 6; filaments 0.2–0.5 mm long, clavate, thick, free or shortly connate at base, glabrous; anthers 0.2–0.3 mm long, erect, connective thicker adaxially forming a keel (Fig. 22 G–H), not overgrowing thecae apically. *Pistillate flowers* ca. 1.6 mm long, brownish; pedicels ca. (0–)1.7 mm long; terete, indumentum as on pistillate inflorescence; bracteoles 3, ca. 0.4 × 0.2 mm, oblong or ovate, fleshy, glabrous adaxially, brownish tomentellous abaxially; sepals 6, weakly concave, fleshy, indumentum as on bracteoles; outer sepals ca. 0.5 × 0.3 mm, ovate or oblong; inner sepals ca. 1.5 × 0.9 mm, obovate tips erect to reflexed past anthesis; petals 3(4), ca. 0.9 × 0.6 mm, opposed to and/or alternating with the carpels, spatulate, weakly concave, membranous, glabrous adaxially, glabrous to sparsely light brown tomentellous abaxially, clawed at base, apex acute to retuse; carpels 3(4), ca. 0.6 × 0.5 mm, brown tomentose; style ca. 0.4 mm long. *Infructescences* axes ca. 4 (–6) × 0.3–0.4 cm, indumentum as on inflorescences; fruiting pedicels inconspicuous; carpophores ca. 2.7 × 4.3 mm, subglobose (seen in immature fruits only), brownish to creamy villose. *Drupelets* ca. 2.9 × 2 cm, ellipsoid to oblongoid (Fig. 23G), conspicuously eccentrically attached, base obtuse, stylar scar not apparent; exocarp 1.8–1.9 mm thick, surface rugose, brownish villous, granular when dried; mesocarp thin and mucilaginous; endocarp 2–2.5 × 1.1–1.5 cm, papyraceous, surface smooth. *Seeds* with embryo 4–6 cm long, cotyledons slightly unequal.

#### Distribution and ecology.

Lowland Amazonia in Brazil, Colombia, and Peru (Fig. 21 –fertile and only a few sterile ones are mapped), at elevations of 120–300 m (550 m in Bagua, based on *Vásquez et al. 19467*, a sterile collection from Bagua, in Peru, here identified tentatively) in tropical wet forest. It is also expected in the eastern lowlands of Ecuador (see below). Staminate flowering specimens were collected in February, July and September–November; the only pistillate flowering specimen was collected in November and fruiting specimens were collected in February and March.

#### Common names and uses

(sterile specimens are indicated as st). **Brazil**: component of curare made by Tecuna Indians, “atinupa” (Krukoff and Smith 1937, Krukoff and Moldenke 1938, Macbride 1938), (*Krukoff 7535*, st; *Krukoff 7578*, ♂ fl buds); as ingredient of arrow and dart poison, “bicava” (*Prance et al. 13931*, st); as ingredient of Jamamadi Indian arrow poison “bicafo” (*Campbell et al. P21256*, st); as a contraceptive by the Deni Indians, “beku” (*Prance et al. 16453*, st). **Colombia**: “taufe-lleida” (Huitoto) (*Díaz 10*, st); “arrow poison” (*Naranjo & Wiederhold 16*, st); as curare (*Pinkley 392*, st); “awa puh”, as ingredient of arrow poison (Bara maku), (*Silverwood-Cope 23*, st). **Ecuador**: “oonta”, to make dart poison (*Davis & Yost 943*, st); “ontame”, hunting, (Huaorani), (*Freire & Naranjo 685*, st); “oonta”, used to make blowgun dart poison, (Huaorani), (*King et al. 972*, 977, st); “palahuasca”, as ingredient of curare (Quichua) (*Lewis et al. 13848*, st); “largancho” (*Moya & Reyes 274*, st); “zapepa”, employed in arrow poison (*Naranjo 7*, st); “ontame”, hunting (Huaorani) *Naranjo & Freire 363*, st); “unta”, hunting (Huaorani) (*Naranjo & Freire 635*, st); as curare (*Pinkley 285*, st). **Peru**: “abuta, abote”, used as vermifugue (*Huamán et al. 417*, st); “tseás” as ingredient of curare (Mayna Jívaro) (*Lewis et al. 10224*, st); “macháp” (Mayna Jívaro) (*Lewis et al. 10425*, st); “machaap”, as main ingredient of curare (Achual Jívaro) (*Lewis et al. 11759*, st); “macháp” (Achuar Jívaro), “nakaapur, papur” (Achuar Jívaro), medicinal, to treat leishmaniasis lesions, also as ingredient of curare (*Lewis et al. 14349*, st); “abuta”, to prepare poison for hunting (*Martin & Lau-Cam 1204*, st); “abuta hembra” (*Martin & Lau-Cam 1273*, st); “abuta hembra (*Mathias & Taylor 3555*, st); “abuta” (*Mathias & Taylor 5004*, *5010*, st); “abuta amarilla” (*Rimachi 11394*, ♂ fl); “abuta ancho” (*Schunke 5848*, *5849*, *5850*, and *5851*, all st); “abuta amarilla” (*Tina & Oliveira* 2343, st); “medicinal, (*Vásquez 16803*, st); “abuta negra” (*Woytkowski 5336*, st); “ampi” (*Woytkowski 5354*, *5355*, st).

#### Etymology.

In reference to the Tecuna Indians who used the plant as a source of curare (Barneby and Krukoff 1971),

#### Comments.

Specimens from Brazil (*Prance et al. 13991* and *Campbell et al.* P21256) were previously identified as *C. toxicofera*. Likewise, the common name of “atinupa” attributed to *Chondrodendron polyanthum* or to *Chondrodendron toxicoferum* (= *Curarea toxicofera*) (Krukoff and Smith 1937, Krukoff and Moldenke 1938, Macbride 1938, Barneby and Krukoff 1971) are here confirmed to refer to *Curarea tecunarum*.

#### Conservation status.

The 14 collections evaluated correspond to 11 localities and yielded an Extent of occurrence (EOO) of 712,212 km^2^ and an Area of Occupancy (AOO) of 44 km^2^. The 11 localities, represent 11 subpopulations and 9 locations, four are found within protected areas (one in Parque Nacional Natural Amacayacu and another in Parque Nacional Cahuinarí, both in Colombia and two in Allpahuayo-Mishana National Reserve in Peru). Additionally, one was found in private lands in Peru. Based on several sterile specimens that are here assigned tentatively (and were not mapped nor included in the conservation assessment), the species appears to be distributed across the Amazon basin, hence the estimated AOO may be greater than that reported here. Therefore *C. tecunarum* is assigned a preliminary status of “Least Concern” (LC).

#### Discussion.

Laxly multi-branched staminate inflorescences covered with brownish or creamy tomentellous indumentum and flowers that are usually sessile on irregular higher order branches are unique to *C. tecunarum*. The species shares with *C. barnebyana* and *C. crassa* a tomentellous indumentum on the abaxial leaf surface as well as subglobose carpophores, but the latter two have staminate inflorescences with condensed/contracted primary branches. These species also share some anatomical features (Table 5; discussion of *C. crassa*).

#### Selected specimens examined.

**BRAZIL. Acre**: Sena Madureira, Floresta Estadual do Antymari, ramal do Ouro, ca./abbrev> 30 km da sede Úirapuro (Servicio Florestal), 09°19'47"S; 68°18'12"W, 9 Mar 13, (mat fr), *Medeiros et al. 1091* (NY!). **Amazonas**: Igarapé Curucuhy, São Gabriel, 27 Nov 1945, (♀ fl), *Fróes 21446* (F!, IAN!, NY!); Munic. São Paulo de Olivença, ibid., near Palmares, 11 Sept 1936–26 Oct 1936, (♂ fl), *Krukoff 8370* (A!, BM!, BR!, F [2]!, G!, MO!, NY!, U!). **Rio de Janeiro**: Cultivated in Rio de Janeiro, no specific locality (st), *Glaziou 9610* (P [2]!–a duplicate at F! as “9610” instead = *C. toxicofera*. **Rondônia**: Rodovia, Alvorada-Costa Marques, km 90, colectado no transectum, mata de terra firme, solo areno-argiloso, 2 Jul 1983, (♂ fl), *Silva 6538* (IAN!, MG!, RB-2!); Margen direita do Río Pacáas Novos, entre a 1ra e 2da cachoeira, mata de várzea, 20 Mar 1978, (imm fr), *Ubiratan et al. 218* (GH!, MO!, NY!).

**COLOMBIA. Amazonas**: La Pedrera, Inspección de Santa Isabel, Parque Nacional Natural Cahuinarí, Estación Biológica Puerto Barbados; várzea (rebalse alta) sobre suelos lateríticos, dominada por *Astrocaryum*, 01°28'S; 070°46'W, 300 m, 29 Nov 1990, (♂ fl), *Pipoly et al. 12846* (MO!, NY!). **Comisaría del Putumayo**: Between Río San Miguel and Río Guamués, Aug 1963, (st), *Naranjo 7* (AMES!). **Vaupés**: Right tributary of Rio Macu-Parana, 1–8 Jun 1970, (st), *Silverwood-Cope 23* (AMES!).

**ECUADOR. Napo**: Confluence of Quiwado and Tiwaeno Rivers, 13 Apr 1981, (st), *Davis & Yost 943* (AMES!). **Orellana**: Loreto, Reserva Étnica Huaorani, Comunidad Miwaguno a 140 km al. sur del Coca, vía al. Pindo, bloque 14 (ENCAN), Río Shiripuno, 00°43'32S; 076°43'36"W, 250 m, 9 May 2004, (st), *Freire & Naranjo 685* (MO!, QCNE n.v.). **Pastaza**: Kapawí (Amuntai), Río Pastaza, village area, secondary and primary forests and pastures, 02°31'S; 076°48'W, 235 m, 25–29 July 1989, (st), *Lewis et al. 13848* (MO!).

**PERU. Amazonas**: Bagua, Distrito Imaza, Región del Marañon, Comunidad de Yamayakat, Quebrada Kusu-Chapi, Río Marañon, 04°55'S; 078°19'W, 550 m, Feb 1995, (st), *Vásquez et al. 19467* (MO!). **Loreto**: Maynas, Distrito Las Amazonas, Quebrada Sucusari, bosque maduro en tierra firme, 03°15'S; 072°55'W, 140 m, 1 Feb 1996, (imm fr), *Ortiz et al. 143* (MO!); ibid., (♂ fl), *Ortiz et al. 144* (MO!); Distrito de Iquitos, Estación Experimental de Allpahuayo, IIAP, 04°10'S; 073°30'W, 120 m, 24 Feb 1996, (old ♂ fl), *Ortiz & Ruiz 188* (MO!); Carretera Iquitos-Nauta, km 21, trocha de penetración del fundo Pichiri, en terreno alto y arenoso, 150 m, 18 Oct 1995, (♂ fl), *Rimachi 11394* (MO!). **Pasco**: Oxapampa, vivero, Proyecto Pichis Palcazu, Puerto Bermudez, transect 2, 10°12'S; 074°57'W, 200 m, 16 Jun 1983, (st), *Gentry et al. 42061* (MO!). **San Martin–Ucayali**: Vicinity of Aguaytía, high ground in forest, east of Aguaytía, between Pucallpa road and Río Aguaytía, 28 Jun 1960, (st), *Mathias & Taylor 5004* (F!). **Ucayali**: Aguaytía, Fundo Vista Alegre, 29 Mar 1962, (st), *Schunke 5848* (F! [2]).

**VENEZUELA. Amazonas**: Cerro Neblina base camp on Rio Mawarinuma, mature forest on sandy “ultisol”, 140 m, 23 Apr 1984, (st), *Gentry & Stein 46878* (MO!).

### 8. Curarea tomentocarpa

Taxon classificationPlantaeRanunculalesMenispermaceae

(Rusby) R.Ortiz comb. nov.

urn:lsid:ipni.org:names:77185802-1

Fig. 24

Cissampelos tomentocarpa Rusby, Descr. So. Amer. Pl. 17. 1920. Type: Bolivia. San Buena Ventura, 1400 feet, 22 Nov 1901, (imm fr), *Williams 616* (lectotype, designated by Dorr 1991, pg. 230: NY! [NY320534]; isolectotype: NY! [NY320535]).
Chondrodendron tomentocarpum (Rusby) Moldenke in Krukoff & Moldenke, Brittonia 3: 21. 1938. Type: based on Cissampelos tomentocarpa Rusby
Abuta boliviana Rusby, Mem. New York Bot. Gard. 7: 241. 1927. Type: Bolivia. Rurrenabaque, [Beni: Gral. Jose Ballivian], 1000 ft, 25 Nov 1921, (♂ fl), *White 1812* (holotype: NY! [NY320439]; isotypes: BKL! [2] [image seen], the inflorescence on the left of the BKL00004029 sheet only, leaves do not belong to Menispermaceae, GH! [GH00038884), K!, MICH! [image seen], US! (US-1232429).

#### Description.

Small to medium-sized understory *lianas* about (0.5–)3–10 tall; older stems more or less terete ca. 0.5–1.5 cm diameter; bark greyish to dark brown, with shallow lengthwise fissures and scarcely tuberculate-lenticellate; branchlets greyish to brownish hispidulous. *Leaves*: blades 7–22 × 5–14 cm, ovate, narrowly ovate or elliptic; membranous when juvenile, chartaceous when mature or when directly exposed to sunlight; surfaces conspicuously discolorous, lustrous and glabrous adaxially, finely silvery tomentellous abaxially, intermixed with some coarser hairs, concealing the surface at all stages, base truncate, obtuse, rounded or shallowly cordate, apex acute, long-acuminate when juvenile, 3(5) palmati- or plinerved, innermost pair of main veins acrodromous imperfect at all stages, midrib shallowly impressed to flat adaxially, conspicuously raised abaxially, secondary veins 2–4 pairs, arising above the middle of the blade, impressed adaxially, raised abaxially, veinlets slightly prominent adaxially, conspicuously raised abaxially, but then concealed by the indumentum; petioles 3–11 cm long, ridged, rufescent or greyish strigillose to glabrate, distal pulvinus moderately conspicuous, rugulose, sometimes weakly flat adaxially. *Staminate inflorescences* solitary or fascicled, cauliflorous or axillary, thyrsi, densely brownish rufescent or silvery hispidulous, trichomes moderately long; axes 6–15(–25) cm long; primary branches slender, 1.7–3.7(–7.5) cm long, with 2–3(–5) branching orders laxly arranged; bracts 0.7–1.3 mm long, narrow ovate to ovate, concave, ascending, moderately fleshy, glabrous adaxially, brownish, rufescent or silvery villose abaxially, trichomes moderately spreading. *Pistillate inflorescences* fascicled, cauliflorous, moderately slender, few-flowered thyrsi, these with the primary branches reduced to single flowers, light brown or rufescent hispidulous; axes 4.2(–8.6) cm long; bracts 0.8(–1.2) mm long, ovate, concave, scarcely fleshy, glabrous adaxially, brown tomentellous abaxially. *Staminate flowers*, 1.2–2 mm long, brownish, greyish, yellowish or greenish; pedicels 0.8–2.8 mm long, terete, thick, indumentum as on the staminate inflorescence; bracteoles 1–4, 0.2–0.4 × 0.2–0.4 mm, ovate, moderately fleshy, glabrous adaxially, rufescent or light golden villose abaxially, trichomes moderately spreading; sepals 6(9), 2(3)–whorled, glabrous adaxially, abaxially with a rufescent to silvery or greyish indumentum, trichomes moderately spreading; outer sepals 0.5–1.1 × 0.4–0.8 mm, ovate or elliptic, base truncate, apex obtuse; (middle sepals 1.2–1.6 x 0.8–0.9 mm, narrow ovate to ovate, base and apex obtuse, moderately fleshy); inner sepals 1.1–2.2 × 1.1–1.7 mm, ovate or elliptic, base obtuse or cuneate, apex acute or obtuse, tip of inner sepals mostly erect past anthesis; petals 6(9), 0.6–1.3 × 0.4–0.9 mm, the inner one slightly smaller and narrower, obovate-trilobed (spatuliform-trilobed), strongly concave, membranous, glabrous adaxially, glabrous to sparsely silvery tomentellous abaxially, base cuneate or distinctly clawed, lateral margin inflexed, partially clasping the filaments, apex obtuse or truncate; stamens 6, filaments 0.4–0.9 mm long, clavate, rarely weakly terete, free (shortly connate at base), glabrous; anthers 0.2–0.3 mm long, erect, connective frequently thinner apically, thecae splitting into two halves, connective thicker adaxially (protruding as a hump at the base or at the apex of thecae). *Pistillate flowers* ca. 1.8 mm long, yellowish to brownish; pedicels ca. 6.4 mm long, terete, indumentum as on the pistillate inflorescence; bracteoles 2, ca. 0.4 × 0.3 mm, ovate, weakly concave, fleshy, glabrous adaxially, brownish, silvery or rufescent villous abaxially, trichomes moderately spreading; sepals 6–9, weakly concave and scarcely fleshy, in 2–3 whorls, indumentum as on bracteoles; outer sepals ca. 0.5 × 0.4 mm, ovate, base truncate, apex acute or obtuse; (middle sepals, ca. 0.9 × 0.8 mm, ovate to broadly ovate, base and apex obtuse); inner sepals ca. 1.5 × 1.4 mm, elliptic, apex and base obtuse, tips erect to weakly reflexed past anthesis; petals 3(6), ca. 1.2 × 0.7 mm, spatulate, weakly concave, membranous, glabrous adaxially and abaxially, base cuneate or clawed, apex obtuse, (when six petals, the inner ones usually narrower); carpels 3(4), ca. 0.6 × 0.3 mm, pilose; style ca. 0.6 mm long. *Infructescences* moderately slender, rufescent, brownish or silvery villous, indumentum spreading; axes 1.7–6 × 0.2–0.5 cm; fruiting pedicels (of mature fruits only) 0.4–1.6 cm long, terete; carpophores 4.1–10.6 mm long, free, clavate or terete, brownish-silvery velutinous. *Drupelets* 1.3–2.4 × 0.8–1.5 cm, dull orange to yellow when ripe, oblongoid or ellipsoid, eccentrically attached, shortly stipitate, base attenuate or truncate, stylar scar usually conspicuous; exocarp 0.7–1.5 mm thick, surface usually strongly muriculate, brownish to greyish hispidulous; mesocarp mucilaginous; endocarp 1.3–2.5 x 0.7–1.2 cm, papyraceous, surface weakly reticulate by slightly prominent fibers throughout. *Seeds* with embryo 2.6–4.4 cm long, cotyledons equal.

#### Distribution and ecology.

The species ranges from eastern Ecuador (Napo Prov.) through central-southern Peru (Huánuco, Madre de Dios, Puno, San Martín and Ucayali departments), northern Bolivia (Beni, La Paz, and Pando) to western Brazil (Acre and Rondônia) (Fig. 25). It occurs mostly on non-flooded forest, from about 71 m in Acre (Brazil) up to 1550 m elevation in Franz Calabatea (Bolivia).

**Figure 24.**
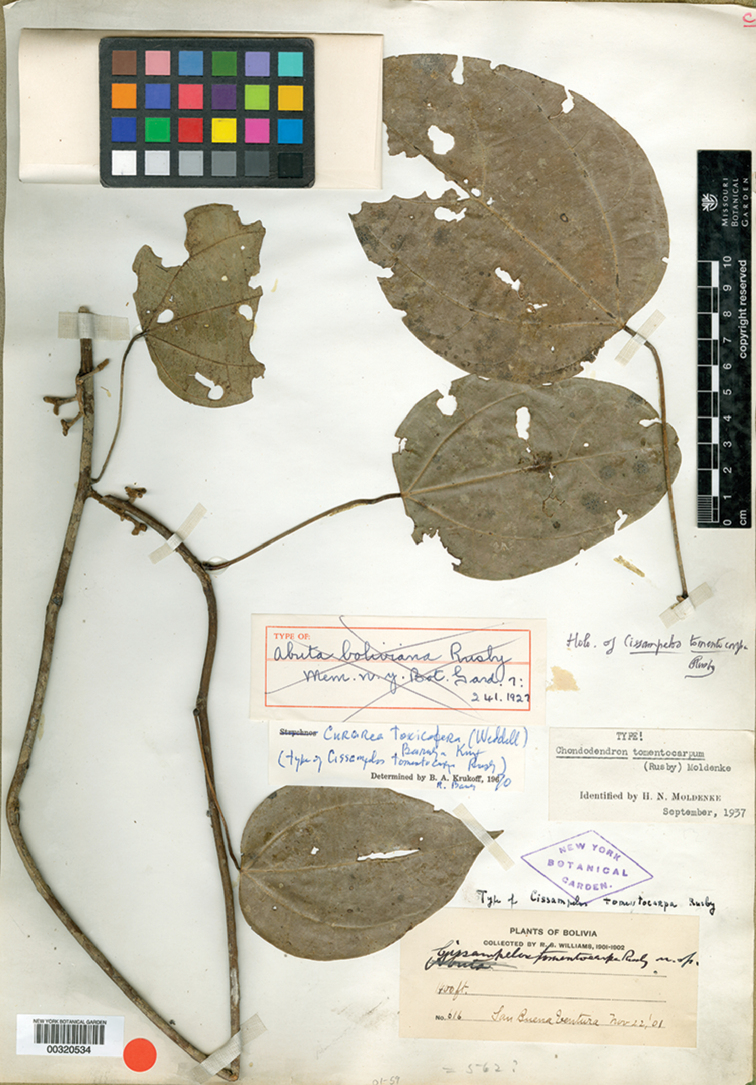
*Curarea tomentocarpa* (photograph of the type of *Cissampelos tomentocarpa*, *Williams 616*, NY).

**Figure 25.**
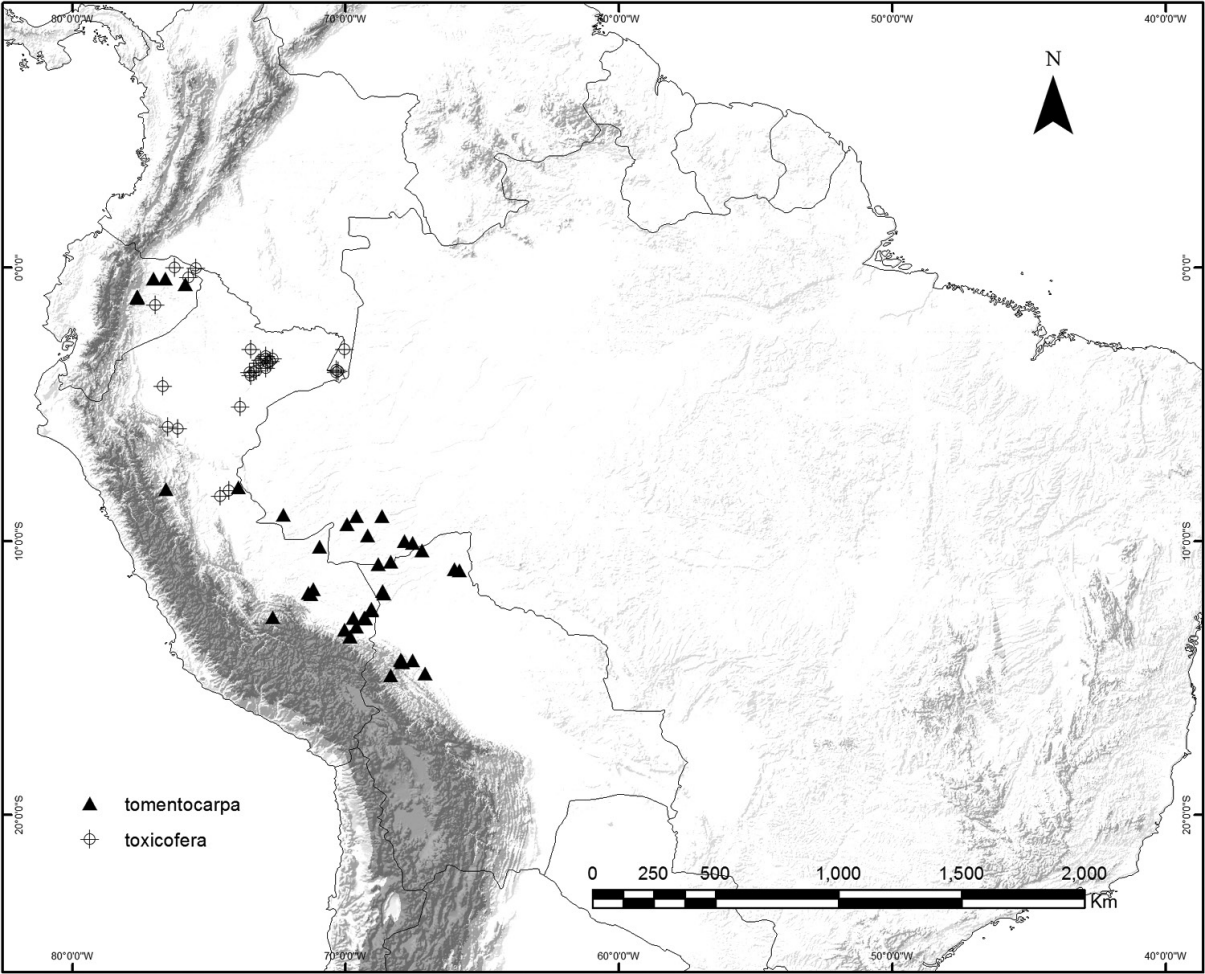
Geographic distribution of *Curarea tomentocarpa* and *C. toxicofera*.

#### Common names and uses.

**Bolivia**: “betchusajaja” (Tacana name), and “huevo de mono” (Spanish name), fruits edible when ripe (*DeWalt et al. 865*, imm fr); “chocolatillo” (*Perry et al. 654*, st); “uvilla”, fruit edible, but astringent (*Smith et al. 12882*, mat fr). **Brazil**: “cipó cacau” (Portuguese) (*Daly et al. 9172*, ♂ fl); “cipó cacauí” (*Daly et al. 9233*, imm fr; *11472*, imm fr). **Ecuador**: “curare” (*Palacios et al. 881*, ♂ fl); “chichico huasca” (Kichwa), fruits edible (*Reyes & Carrillo 827*, mat fr; *Carrillo & Reyes 695*, mat fr). **Peru**: “ampihuasca amarilla” (*Schunke 2981*, mat fr); “ampihuasca delgadito” (*Schunke 6836*, ♂ fl, MO sheet sterile); “ampihuasca negra” (*Schunke 7125*, ♂ fl).

#### Etymology.

Possibly in reference of the hispidulous-muriculate surface of the drupelets which is frequently described as warty in herbarium specimens.

#### Conservation status.

The Extent of Occurrence (EOO), calculated for the 67 collections corresponding to 45 localities of *C. tomentocarpa*, is 984,251 km^2^ and its Area of Occupancy (AOO) is 176 km^2^. Although the species is not abundant where it occurs and, of the 39 subpopulations, 10 are found within protected areas across its distribution in Bolivia, Ecuador and Peru. This suggests that its AOO may be larger than the one estimated here; therefore, *C. tomentocarpa* is assigned a preliminary category of “Least Concern” (LC).

#### Discussion.

The species is distinguished by its staminate inflorescence with densely brownish, rufescent or silvery hispidulous, moderately long and spreading indumentum and by the anthers with thin connective that the former frequently split into two halves. The only pistillate inflorescence available in this study has the primary branches reduced to single flowers, thus appearing racemiform. However, fruiting specimens from the same areas, that are similar in other features, appear to be clearly thyrsoid. It is likely that the inflorescence becomes more differentiated during further development, but that has not been confirmed here. The drupelets are always strongly muriculate. However, a single fruiting collection from Huánuco in central Peru (*Schunke 2981*) has a smooth surface and velutinous indumentum, although no pistillate flowers from the area were available in this study. The quantitative characters evaluated here in a few staminate plants from San Martín (*Gentry 25720*; *Schunke 6836*, *7125*) and from Ucayali (*Schunke 2690*) that resemble, in leaf shape and indumentum, the fruiting specimen mentioned above, completely overlap with those of *tomentocarpa* (Fig. 11B). Hence, I am unable to match variation in the staminate specimens to that in the fruiting material. Therefore, all of these collections are here provisionally placed under *C. tomentocarpa*.

#### Selected specimens examined.

**BOLIVIA. Beni**: Vaca Diez, 9.2 km N from the road between Riberalta and Guayaramarín, on the old road to Cachuela Esperanza, primary forest, 11°05'S; 065°50'W, 230 m, 18 Sep 1981, (mat fr), *Solomon 6317* (MO!, NY!). **La Paz**: Franz Tamayo, Parque Nacional Madidi, laguna Chalalan, senda Jaguar, bosque amazonico preandino, 14°25'30S; 067°55'16"W, 300 m, 26 Sep 2006, (mat fr), *Araujo-M. et al. 3131* (LPB n.v., MO!, NY n.v.); Abel Iturralde, Comunidad de Buena Vista, parcela permamente de estudio etnobotánico, 3 km al. NE de Buenavista, 14°22'S; 067°33'W, 180 m, 12 Apr 1995, (imm fr), *DeWalt et al. 865* (MO!); Parque Nacional y Area de Manejo Integrado Madidi, Laguna Chalalán, entrando 45 min., sobre orilla izquierda de Río Tuichí, vegetación borde de laguna, dominada por *Mauritia flexuosa* y helechos arbóreos, 14°26'S; 067°55'W, 450 m, 23 Apr 1997, (mat fr), *Paniagua & Beck 1171* (LPB n.v., MO!). **Pando**: Manuripi, Carretera entre Cobijá y Chicé km al. S del Río Manuripi, 11°55'S; 068°36'W, 200 m, 1 Oct 1991, (mat fr), *Perry et al. 413* (LPB n.v., MO!).

**BRAZIL. Acre**: Mun. Sena Madureira, basin of Rio Purus, Rio Macauã, Colocacãa Apuí, river descending, 3 m below high water mark, terra firme forest on poorly drained soil, undulating terrain dissected by numerous streams, 09°48'S; 069°11'W, 29 Mar 1994, (imm fr), *Daly et al. 8073* (MO!, NY! [image seen]); Rio Acre, Antimary, Jul 1904, (fr), *Huber 4286* (B! frag., MG, n.v.); Km 16 from Rio Branco on Rio Branco-Brasiléia road, 20 Oct 1980, (♂ fl), *Lowrie et al. 595* (NY!, US [2]!); Estrada Rio Branco-Brasiléia, km 42, mata primaria de terra firme, 10°00'S; 067°50'W, 16 Oct 1980, (mat fr), *Nelson 716* (NY!).

**ECUADOR. Napo**: Tena, Estación Biológica Jatun Sacha, bosque muy húmedo tropical, suelo principalmente compuesto por arcilla roja, 01°04'S; 077°36'W, 450 m, 29 Jun 1996, (imm fr), *Ortiz & Vargas 197* (MO!); ibid., 3 Jul 1996, (♂ fl), *Ortiz & Vargas 199* (MO!); Río Arajuno, Sola Cocha, bosque muy húmedo tropical, suelo rojo arcilloso (ultisol), colinas pendientes, 01°07'S; 077°36'W, 500 m, 23–27 Oct 1985, (♂ fl), *Palacios et al. 881* (AAU n.v., MO-2!, NY!, QCA n.v., QCNE n.v.); Estación Biológica Jatun Sacha, bosque muy húmedo tropical, bosque primario sobre suelos rojos de colinas, 01°04'S; 077°37'W, 450 m, 06–14 Oct 1988, (♂ fl), *Palacios 3106* (MO!, NY!).

**Orellana**: Reserva Florística El Chuncho, 5 Km al. norte de Coca, bosque húmedo tropical, suelos rojos sobre colinas disectadas, parcela permanente, 00°25'S; 077°01'W, 250 m, 23 May 1993, (mat fr), *Palacios 10725* (MO!, QCNE n.v.); Aguarico, Parroquia Capitán Agusto Rivadeneira, comunidad Chiro Isla (Kichwa), bosque de matorrales, 00°37'28S; 075°51'31"W, 200 m, 22 Feb 2005, (mat fr), *Reyes & Carrillo 827* (MO!, QCNE n.v.).

**PERU. Cusco**: La Convención, Río Manguriari (Manguyari), Tropical Forests –Alto Urubamba; upstream to Río Manguriari, forest edge, 12°47'S; 072°40'W, 750 m, 2 Feb 1991, (st), *Núñez & Ortíz 12765* (MO!). **Huánuco**: Pachitea, Dtto. Puerto Inca, Bosque Nacional de Iparia, región de bosque seco tropical a lo largo del Río Pachitea, cerca del pueblo de Puerto Inca (unos 85 km en distancia lineal de la confluencia con el Río Ucayali), 12 Jan 1969, (mat fr), *Schunke 2981* (F!, G!, NY!, US!). **Madre de Dios**: Manu. Parque Nacional de Manu, in forest near Cocha Cashu Station, on an old ox-bow lake of the Rio Manu, 11 Aug 1973, (mat fr), *Foster 2543* (NY!); Vicinity of Cocha Cashu, 400 m, 4 Aug 1977, (mat fr), *Foster & Terborgh 6480* (F!, NY!); Trails 4, 5 and 9, Cashu Cocha Camp, Manu National Park, non-inundated forest on alluvial soil, 380 m, 21 Oct 1979, (♀ fl), *Gentry et al. 27080* (F!, MO!, NY!); Tambopata Reserve, Río Tambopata at mouth of Río D’Orbigny, non-transect, [12°50'S; 069°17'W], 200 m, 2 Mar 1981, (imm fr), *Gentry & Young 31927* (MO!, USM n.v.); Zona reservada de Tambopata, 12°49'S; 069°18'W, 280 m, 10 Aug 1990, (imm fr), *Reynel & Meneses 5025* (MO!); Dist. Puerto Maldonado, Cusco Amazónico, bosque Primario, 250–300 m, 13°08'S; 069°36'W, 22 Nov 2002, (♂ fl), *Valenzuela et al. 975* (CUZ, n.v., MO!, USM, n.v.). **Puno**: Carabaya, Cabeceras del Río Candamo, 550–600 m, 13°15'S; 070°02'W, 24 Oct 1996, (mat fr), *Cornejo 2650* (MO!, fruit not seen); Río Candamo, fila at mouth of Río Guacamayo, ridge top forest with cloud forest aspect, 13°30'S; 069°50'W, 800 m, 26 May 1992, (imm fr), *Gentry et al. 77256* (MO!). **San Martin**: Fundo Curareland near Tinanta, 20 km NW of Tocache, at N edge of palm plantation, mature forest and forest edge, on alluvial soil near Rio Huallaga, 08°06'31"S; 076°3314"W, 500 m, 14 Mar 1979, (♂ fl), *Gentry et al. 25720* (F!, MO!, NY!, USM!); Mariscal Caceres, Dtto. de Tocache Nuevo, Santa Rosa de Mishollo (margen derecha del Rio Mishollo), 500 m, 15 Aug 1976, (♂ fl), *Schunke 6836* (MO!, NY!); Quebrada de Huaquisha (margen derecha del Rio Huallaga), 450 m, 2 Jul 1974, (♂ fl), *Schunke 7125* (F!, MO!, USM!). **Ucayali**: Coronel Portillo, Vicinity of LSU base camp, Quebrada Shesha (tributary of Río Abujao), ca. 65 km NE of Pucallpa, 08°02'S; 073°55'W, 250 m, 25 Jun 1987, (imm fr), *Gentry & Díaz 58512* (F!, MO!); [Distrito de] Iparia, a lo largo del Río Ucayali, cerca del pueblo de Iparia (unos 80 km arriba de la confluencia con el Río Pachitea), 300 m, 26 Aug 1968, (♂ fl buds & fl), *Schunke 2690* (F!, G, NY!); Dtto. Purús, Rio la Novia, margen derecha del caserio de la comunidad nativa San José, bosque alto, terreno húmedo, 10°12'S; 070°57'W, 180 m, 13 Feb 2002, (mat fr), *Schunke & Graham 14779* (MO!).

### 9. Curarea toxicofera

Taxon classificationPlantaeRanunculalesMenispermaceae

(Wedd.) Barneby & Krukoff

Figs 26A–H , 27A–F

Curarea toxicofera (Wedd.) Barneby & Krukoff, Mem. New York Bot. Gard. 22(2): 9. 1971. fig. 1.
Cocculus toxicoferus Wedd., in Castelnau, Expéd. Part. Cent. Amér. Sud. 5: 22. 1851. Type. Peru. “Vulg. Pani−base d’un poison pour les flèches utilisé chez les Indiens Pebas−Hte. Amazone”, [1847], *Castelnau s.n.* (sterile) (lectotype designation effected by Krukoff and Moldenke 1939, pg. 338: P! [P00048602] which is annotated as Cocculus toxicoferus in the hand of Weddell and has the eight leaves mentioned by Krukoff and Moldenke. Moreover, this specimen was also annotated in 1939 by Moldenke as “Type”, photograph at NY!; presumed isolectotypes: F! [F-893667, frag.], P!). Peru, Florida, Río Putumayo, at mouth of Río Zubineta, forest, 200 m, Mar 1931–Apr 1931, (♂ fl), *Klug 2042* (epitype, designated here: MO!; isoepitypes: BM!, F!, GH!, K!, NY!, US!). Note: Following Art. 9.8 of the Melbourne Code (MacNeill et al. 2012), I am here designating an epitype to serve as an interpretative type of Cocculus toxicoferus, whose sterile condition makes it ambiguous for identification purposes.
Chondrodendron toxicoferum (Wedd.) Moldenke & Krukoff, Brittonia 3(2): 338. 1938. Type: Based on Cocculus toxicoferus Wedd., in Castelnau Expéd. Part. Cent. Amér. Sud. 5: 22. 1851.
Hyperbaena polyantha Diels, Verh. Bot. Ver. Brand. 50: 73. 1908. Type: Brazil. Amazonas: Juruá Miry, Lago de Esperança, Aug 1901, (♂ fl), *Ule 5631* (lectotype designation effected by Krukoff and Moldenke 1938, pg. 23: B!, F neg. 4985]; isolectotypes: CORD! [image seen], F! [F-1014713, frag.], G!; K! [image seen], MG!, [image seen], NY! [NY00320584, frag.]).
Chondrodendron polyanthum (Diels) Diels, in Engler, Pflanzenr. 4(94): 78. 1910. Type: Based on Hyperbaena polyantha Diels.
?Chondrodendron bioccai G. Lusina, Revista Mus. Paul. Univ. São Paulo II. 8: 227, figs 1–2. 1954. Type: Brazil? Locality unknown, collector unknown.

#### Description.

**Figure 26.**
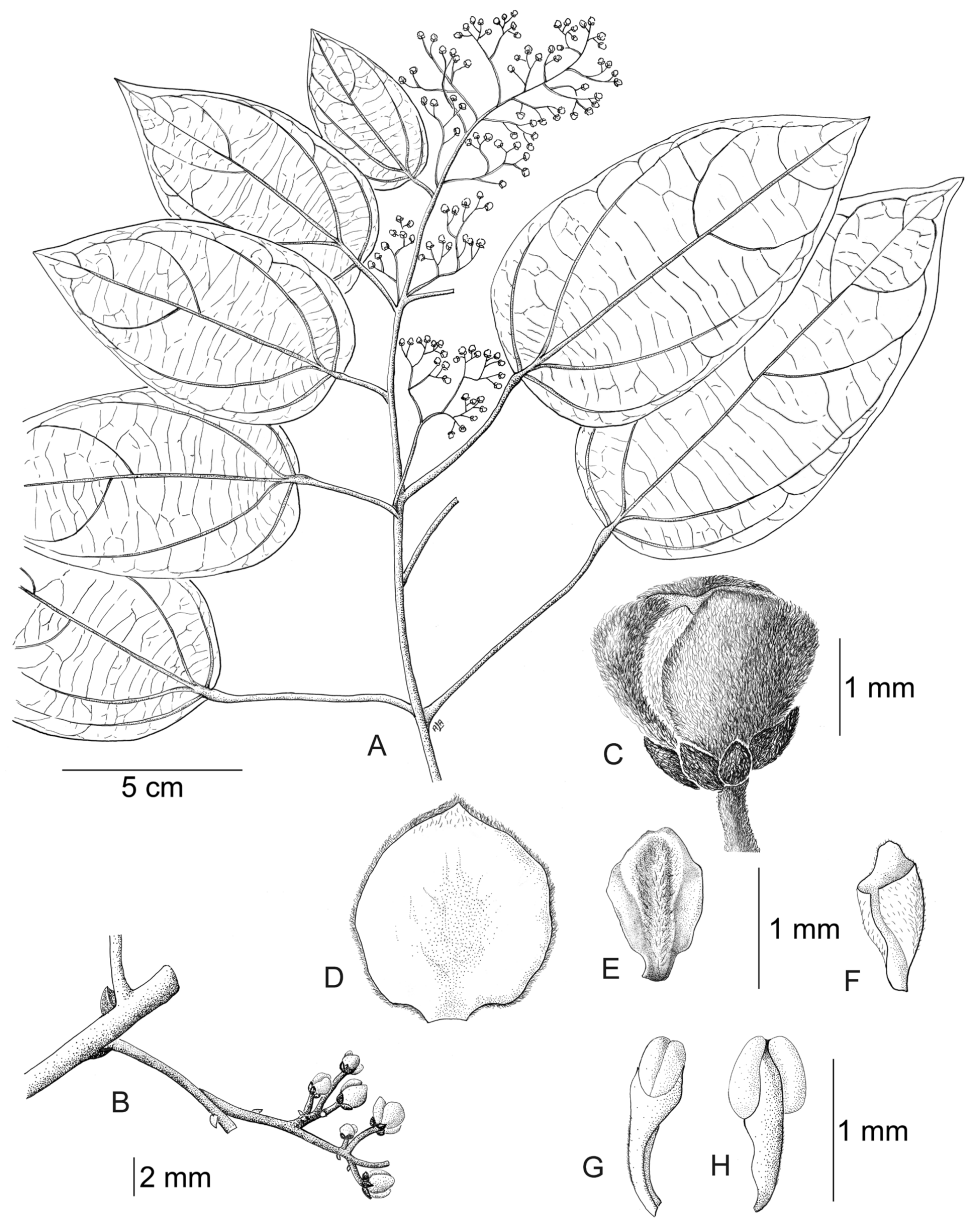
*Curarea toxicofera* staminate plant: **A** flowering branch **B** detail of inflorescence **C** flower bud **D** inner sepal, adaxial surface **E–F** outer and inner petals, abaxial and latero-adaxial surfaces **G–H** outer stamens, lateral and abaxial surfaces (**A–B** based on *Ule 5631* **C–H** based on *Encarnación 1094*).

**Figure 27.**
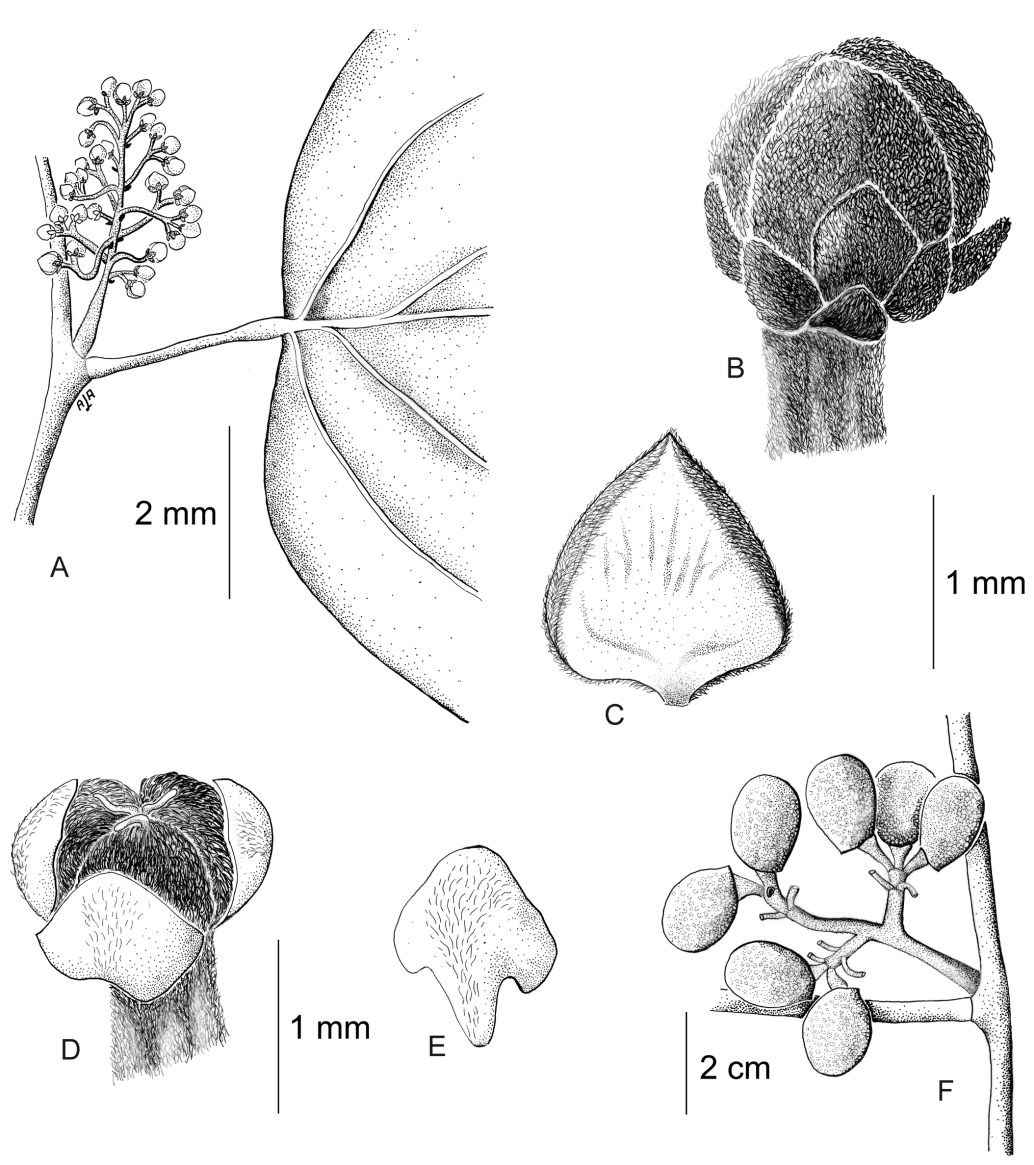
*Curarea toxicofera* pistillate plant: **A** flowering branch **B** flower bud **C** innermost sepal, adaxial surface **D** petals and carpels **E** petal abaxial surface **F** infructescence (**A–E** based on *Gentry et al. 21624* **F** based on *Revilla et al. 2566*).

Medium-sized understory *lianas* about 10–12 m tall; older stems more or less terete or less frequently flattened, then ca. 3 cm wide; bark greyish to dark brown, with shallow lengthwise fissures; branchlets brownish, greyish to silvery puberulent-strigillose. *Leaves*: blades 8–24 × 5–17 cm, narrowly to broadly ovate, chartaceous at all stages; surfaces discolorous, lustrous and glabrous adaxially, finely silvery tomentellous abaxially, sometimes creamish when older, indumentum mostly concealing the surface at all stages, base truncate, obtuse or shallowly cordate, apex acute or acuminate, long-acuminate when juvenile, 3–5(–7) palmati- or shortly plinerved, innermost pair of main veins acrodromous imperfect at all stages, midrib adaxially shallowly sunken at the base, becoming flat, conspicuously raised abaxially, secondary veins 2–3(–5) pairs, arising above the middle of the blade, raised on both surfaces, more conspicuous abaxially, veinlets slightly prominent adaxially, more conspicuous abaxially; petioles (2.7–)6–25 cm long, ridged, brownish to silvery strigillose-tomentellous or glabrate, distal pulvinus more conspicuous, rugulose, rounded, sometimes more or less flat adaxially. *Staminate inflorescences* solitary or fascicled, cauliflorous or axillary thyrsi, brownish, greyish or silvery strigillose-tomentellous, (trichomes spreading); axes, 10–42 cm long (less frequently simple dichasia arising in young terminal shoots, then the dichasia axes 2–4 cm long) (Fig. 26A), densely brown; primary branches 1.4–9.4 cm long, with several (2–4) branching orders, these laxly arranged; bracts 0.5–1.2 mm long, narrow ovate to ovate, concave, moderately fleshy, indumentum as on the inflorescence. *Pistillate inflorescences* solitary or fascicled, cauliflorous, moderately stout, few-flowered thyrsi (Fig. 27A), brownish strigillose- tomentellous; axes ca. 2.5 cm long; primary branches 0.7 cm long; bracts ca. 0.8 mm long, ovate, concave, moderately fleshy, glabrous adaxially, brown strigillose-tomentellous abaxially. *Staminate flowers* 1.6–2.4 mm long, greenish, green-yellowish or whitish; pedicels 1.6–6.0 mm long, mostly slender, ridged, indumentum as on staminate inflorescence; bracteoles 1–2, 0.2–0.6 × 0.1–0.4 mm, ovate, moderately fleshy, glabrous adaxially, greyish or silvery villous-tomentellous abaxially; sepals 6, glabrous adaxially, greyish or silvery villous-tomentellous abaxially; outer sepals 0.5–1.4 × 0.3–0.8 mm, narrowly ovate or elliptic, base truncate, apex obtuse; inner sepals 1.5–2.4 × 0.8–1.8 mm, elliptic, ovate, oblong or weakly obovate, base obtuse, cuneate or shortly clawed, apex acute or obtuse, tips mostly strongly reflexed past anthesis; petals 6, 0.7–1.6 × 0.6–1.1 mm, inner ones slightly smaller and narrower, obovate-trilobed or spatulate, weakly concave, membranous, glabrous adaxially, sparse silvery tomentellous abaxially, base cuneate or distinctly short- to long-clawed, lateral margins inflexed, partially clasping the filaments, apex obtuse or truncate; stamens (5)6, filaments 0.3–)0.7–1.4 mm long, clavate or clavate-sigmoid, free (shortly connate), glabrous adaxially, glabrous to mostly silvery tomentellous abaxially; anthers 0.3–0.6 mm long, erect or weakly incurved, especially when older, connective thicker adaxially and protruding as a hump (Fig. 26G) or as a keel at the base of thecae (also apically); thecae not separating apically and, for the most part, not immersed in the connective. *Pistillate flowers* ca. 2.1 mm long, green; pedicels ca. 1.1 mm long, ridged, brown strigillose-tomentellous; bracteoles 3, ca. 0.4 × 0.3 mm, ovate, fleshy, weakly concave, glabrous adaxially, brownish villose-tomentellous abaxially; sepals 6–9, weakly concave and slightly fleshy to fleshy, glabrous adaxially, brownish, greyish to silvery villous-tomentellous abaxially; outer sepals ca. 0.7 × 0.6 mm, ovate, base truncate, apex acute; middle sepals ca. 1.6 × 1.8 mm, ovate to broadly ovate, base truncate, apex acute, inner sepals ca. 2.0 × 1.3 mm, broadly ovate, obovate, spatulate, elliptic or rhombic, base obtuse or truncate, apex acute or rounded, tips strongly reflexed past anthesis; petals 3, ca. 1.6 × 1.5 mm, spatulate, membranous, weakly concave, glabrous adaxially, moderate silvery tomentellous abaxially, clawed at base, apex obtuse or retuse; carpels 3, ca. 0.7 × 0.7 mm, brown villous tomentellous; style ca. 0.7 mm long. *Infructescences* axes (2–)8 × 0.3–0.7cm, indumentum as on pistillate inflorescences; fruiting pedicels 1.3–3.5 mm long, terete, slender to moderately stout; carpophores 3.8–11.3 mm long, free or basally connate up to 1/3 of their lengths, claviform or terete, spreading or incurved distally, brown velutinous-hispidulous. *Drupelets* 1.8–2.1 × 0.9–1.3 cm, dull orange to yellowish when ripe, oblongoid, ellipsoid or subglobose (Fig. 27F), eccentrically attached, base attenuate or truncate, (shortly stipitate); stylar scar conspicuous; exocarp 0.6–0.8 mm thick, surface smooth, rugulose or weakly muriculate, silvery to greyish velutinous; mesocarp mucilaginous; endocarp 0.7–0.9 × 1.5–1.8 cm, papyraceous to chartaceous, smooth, with slightly prominent fibres or weakly rugulose throughout. *Seeds* with embryo 3.2–4 cm long, cotyledons equal.

#### Distribution and ecology.

In north-western Amazonia, including the eastern slopes of the Andes in Peru (Fig. 25). The species commonly grows in periodically flooded forests, but also in non-flooded lowland forests and up to 350 (500) m in elevation. Flowering and fruiting material were collected all year round.

#### Common names and uses

(sterile specimens are indicated as st). **Brazil**: fruits edible (*Fróes 26364*). **Colombia**: “taufe-lleida” (*Diaz 36*, st); “Ñamitá” (en Karijona) (*García-Barriga 14578*, st); cure for fevers, antimalarial (*Grassl 10076*, st); said to be “formerly used by Karijona Indians in arrow-poison” (Krukoff and Barneby 1970) (*Schultes 5526*, st). **Ecuador**: to hunt wild animals, “ambi-huasca” (Quichua) (*Cerón et al. 39668*, st). **Peru**: “pani’ (*Castelnau s.n.*,1851); “abuta amarilla, “isaveño” (Huitoto) (*Martin & Lau-Cam 1266*, st); “ampihuasca” (*Mathias & Taylor 3900*, imm fr); “abuta amarilla” (*Rimachi 10636*, mat fr; *Tina & Tello 2066*, ♂ fl); “sarívana” (*Weiss 132*, st); “ampihuasca”, medicinal (*Woytkowski 108*, st); “ampihuasca” (*Woytkowski 5108*, st); “abuta negra” (*Rimachi 10503*, st).

#### Note.

Sterile collections such as *Fox 12* (K), from Peru and *Schultes 3522* from Colombia, had previously been identified as *Chondrodendron toxicoferum* (= *Curarea toxicofera*) (Krukoff and Barneby 1970), the first being reported as “poison used for blow pipe” and the second as a source of curare with the common name of “sa pe pa” (Kofán) (Krukoff and Barneby 1970). Both specimens cannot be determined with certainty and are here tentatively identified as *Curarea* sp.

#### Etymology.

Presumably in reference to the toxic effects attributed to the plant, as the type specimen was collected in the Pevas region, amongst the Yaguas who use the plant in the preparation of arrow poison (de Castelnau 1851: 22).

#### Conservation status.

The Extent of Occurrence (EOO), based on 34 collections corresponding to 28 localities of *C. toxicofera*, is calculated as 419,469 km^2^ and an Area of Occupancy (AOO) of 108 km^2^. The 28 localities correspond to 27 subpopulations, of which two are within protected areas in Ecuador and four subpopulations are found in private reserves near Iquitos, Peru. In addition the species is widespread across its distribution. Therefore, *C. toxicofera* is assigned a preliminary category of “Least Concern” (LC).

#### Discussion.

The typical form of *C. toxicofera* as circumscribed here, ranges from the Amazonian lowlands of Ecuador, Colombia, Peru and Brazil (including the sterile type specimen of *Cocculus toxicoferus* Wedd., *Castelnau s.n.*, Peru and the type of *Hyperbaena polyantha* Diels, *Ule 5631*, Brazil).

Individuals are frequently found in periodically flooded forests, but can also be found in non-flooded forests up to 500 m in elevation. Most have ovate leaves with 5 main veins and staminate and pistillate inflorescences with very short, brownish to greyish, appressed indumentum, but slightly longer (up to 0.3 mm long) and silvery or cream indumentum is also observed in specimens from the lower Amazon basin in Brazil (e.g. *Prance 11272*, *Fróes 29639* and *Ducke 2134*).

In the staminate condition, *C. toxicofera* is readily distinguished from *C. tomentocarpa* by its brown strigillose-tomentellous indumentum (*vs.* a rufescent to silvery hispidulous) and its larger flowers –they are the largest in the genus– with greyish to silvery villous-tomentellous, adpressed indumentum (*vs.* rufescent or silvery spreading indumentum). However, separation from *C. iquitana* is more challenging and at present is distinguished by its slender pedicels in staminate flowers (vs. thicker in *C. iquitana*). However, more field and taxonomic work remains to be done in order to be able to confidently match the staminate and pistillate specimens of the taxa involved.

*Chondrodendron bioccai* is here included in the synonymy with hesitation. As discussed by Krukoff and Barneby (1970), the species appears to have been described from two collections (from the region of Rio Tiquiê and the region of Rio Uaupés by Biocca and Giacone, respectively). A holotype was not stated, two photographs (listed as Figs 15, 16, but published as Figs 1, 2) presumably of the original material were published in the protologue (Lusina 1954). Krukoff and Barneby (1970) hesitantly placed *Chondrodendron bioccai* as a synonym of *Chondrodendron toxicoferum* and later as a synonym of *Curarea toxicofera* (Barneby & Krukoff, 1971). The repository of the type collection remains uncertain at the present time and hence the taxonomic identity cannot be established firmly.

#### Selected specimens examined.

**BRAZIL. Acre.** Proje. RADAM-Sub-base de Cruzeiro do Sul-Ponto 7-Sb-18-ZD, 23 Feb 1976, (imm fr), *Marinho 291* (NY!). **Amazonas**: Paraná do Careiro (Boca do Solimões), (varzea, igapó do Lago Capitari), 8 Jun 1948 (♂ fl), *Ducke 2134* (COL!, GH!, NY!, R!); Mun. Eirunepe, Lago Dois Unidus, Ituxy, restinga, 30 Nov 1946, (♂ fl), *Fróes 21802*A (NY!); Beira do lago de Badajós, igapó, 24 Ago 1950, (imm fr), *Fróes 26364* (IAN!); Vicinity of Manaus, Igarapé Ipixuna, Lower Rio Negro opposite Manaus, Igapó, 1 Apr 1971, (♂ fl), *Prance, et al. 11272* (F!, MG!, NY!, US!). **Rondônia**: Esperança (ad ostium fluminis Javari), silva loco alto, high forest, 27 Oct 1945, (♂ fl bud), *Ducke 1968* (NY!, R!); Rio Machado, curso inferior, igapó, Jan 1981, (♂ fl), *Goulding 1187* (MG!).

**COLOMBIA. Amazonas**: Quebrada El Mochilero, afluente del Yarí, márgenes y zona de rebalse aluvial, 150 m, 24 Apr 1986, (imm fr), *Galeano et al. 1135* (COL!); Río Caquetá, La Pedrera, 1–4 Oct 1952, (st.), *García-Barriga 14578* (COL!, MO!); Puerto Nariño, Trocha de Panduro a San Martin, bosque no inundable, 03°50'S; 070°20'W, 100 m, 24 Feb 1993, (♂ fl), *Madriñán 702* (MO!); Leticia, Parque Nacional Natural Amacayacu, Centro Administrativo Mata-matá (Inderena), a orillas de la quebrada Mata-matá en zona de várzea, 03°47'S; 070°15'W, 100 m, 11 Mar 1991, (imm fr), *Rudas et al. 1528* (MO!); Corregimiento de Tarapacá; Caño Pupuña (afluente del Río Cotuhé), a las orillas del caño, 02°59'S; 070°02'W, 100 m, 25 Jun 1991, (♂ fl), *Rudas et al. 2512* (MO!). **Vaupés**: Mayaca River, vicinity of Cachivera del Diablo and mouth of river, 300 m, 1 May 1943, (mat fr), *Schultes 5526* (AMES!).

**ECUADOR. Napo**: Rio Yasuni, periodically inundated forest, ca. 80 km upriver from Nuevo Rocafuerte, 225 m, 17 Sep 1977, (♂ fl bud & fl), *Foster 3722* (F!). **Orellana**: Cantón Nuevo Rocafuerte Isla aproximadamente 10 Ha. entre la Laguna de Jatun Cocha, cerca del Río Yasuní, Parque Nacional Yasuní, Igapó, 00°01'S; 075°28'W, 280 m, 17 Sep 1999, (st), *Cerón et al. 39668* (MO!). **Pastaza**: Curaray, SE of the airstrip, rain forest and *Mauritia* várzea, understorey dominated of Melastomataceae, Rubiaceae and palms, 01°22'S; 076°57'W, 250 m, 20 Mar 1980, (mat fr), *Holm–Nielsen et al. 22169* (AAU n.v., NY!). **Sucumbíos**: Lago Agrio, Reserva Cuyabeno, Río Cuyabeno, 2-3 km arriba de Laguna Grande, área inundada estacionalmente por aguas negras, 00°00'S; 076°14'W, 230 m, 16 Nov 1991, (♂ fl), *Palacios et al. 9032* (MO!); Río Zábalo, bosque húmedo tropical, bosque afectado por inundaciones de aguas negras, 00°22'S; 075°45'W, 230 m, 22 Nov 1991, (imm fr), *Palacios et al. 9493* (MO!, NY!).

**PERU. Loreto**: Yanamono, campamento Explorama Lodge, terreno de altura, 03°25'S; 072°45'W, 120 m, 31 May 1979, (mat fr), *Díaz et al. 1187* (MO!); Requena, Caño Yarina, a 300 m de la Base Yarina en la Zona Reservada del Río Pacaya, margen izquierda del Río Ucayali, bosque inundable, 6 Apr 1977, (♂ fl), *Encarnación 1094* (G!, MO!, NY!, US!); Rio Itaya, inundated tahuampa forest, [03°46'S; 073°15'W], 120 m, 20 Mar 1977, (♂ fl), *Gentry et al. 18505* (F!, MO!, NY!); Maynas, tahuampa near Río Amazonas between Punchana and Santa Clara de Nanay, outskirts of Iquitos, 120 m, 3 Feb 1978, (♀ fl & imm fr), *Gentry et al. 21624* (F!, MO!); Alto Amazonas, Balsapuerto (lower Rio Huallaga basin); dense forest, [05°49'S; 76°33'W], 150–350 m, 28–30 Aug 1929, (♂ fl), *Killip & Smith 28665* (F!, NY!); Alto Amazonas, Fortaleza, near Yurimaguas, [05°54'S; 76°07'W], 140 m, Nov 1932, (♂ fl), *Klug 2782* (BM!, F!, G!, GH!, K!, MO, NY!, US!); Distrito Las Amazonas, Quebrada Sucusari, bosque maduro en tierra firme, camino de ACEER hacia el Napo, 03°15'S; 072°55'W, 140 m, 4 Feb 1996, (♂ fl), *Ortiz et al. 157* (MO!); Distrito Iquitos, Rio Itaya, San Juan de Muniches, borde de purma, 16 Oct 1976, (mat fr), *Revilla & Carillo 1501* (F!, MO!, NY!); Indiana, Yanamono, Explorama Lodge, Bosque inundable estacional (aguas blancas), 106 m, 03°28'S; 072°50'W, 19 Feb 1989, (♂ fl), *Vásquez & Jaramillo 11714* (MO!); Explor Napo Camp at Río Sucusari, primary forest, flooded forest, collected from canoe along Río Sucusari, 03°20'S; 072°55'W, 120 m, 22 Mar 1996, (♂ fl buds & fl), *van der Werff & Vásquez 13990* (MO!). **Ucayali**: Prov. Coronel Portillo, Distrito Calleria, Quebrada Pumayacu, margen izquierda del Río Utiquinia, al. borde de la quebrada, en bosque alto, 150–175 m, 08°09'S; 074°15'W, 13 Ma 2003, (♂ fl bud & fl), *Schunke 15323* (F n.v., MO!).

**VENEZUELA. Amazonas**: Embanchure du Guaviare, Plantes du Haut Orenoque, 1887, (♂ fl), *Gaillard 184* (P!); Forest immediately behind “El Tobogan de La Selva” camping area; 35 km south of Puerto Ayacucho, 85 m, 21 Feb 1979, (st), *Plowman 7712* (F!).

## Tribute

This contribution is dedicated to the memory of Alwyn H. Gentry, an extraordinary field biologist, inspiring mentor, dear friend and colleague who was always eager to share his knowledge and who died tragically in a plane crash during the summer of 1993, while surveying a dry forest in western Ecuador.

## Supplementary Material

XML Treatment for Curarea

XML Treatment for Curarea barnebyana

XML Treatment for Curarea candicans

XML Treatment for Curarea crassa

XML Treatment for Curarea cuatrecasasii

XML Treatment for Curarea gentryana

XML Treatment for Curarea iquitana

XML Treatment for Curarea tecunarum

XML Treatment for Curarea tomentocarpa

XML Treatment for Curarea toxicofera

## Supplementary materials

Supplementary material 1 List of specimens examined Data type: Index of specimens examined Explanation note: Index of collectors, collector numbers, and corresponding curarea species. This is an open access article distributed under the terms of the Creative Commons Attribution License (CC BY 4.0), which permits unrestricted use, distribution, and reproduction in any medium, provided the original author and source are credited.Rosa del C. Ortiz
