# Supplementary material for: A taxonomic revision of Curarea Barneby & Krukoff (Menispermaceae)
Source: PhytoKeys. 2018 Jun 21;(100):9–89. doi: 10.3897/phytokeys.100.21828 (PMC6023953; doi:10.3897/phytokeys.100.21828)
Supplement: Supplementary material 1 — List of specimens examined [file phytokeys-100-009-s001.doc]

**Supplementary material**

Author: Rosa del C. Ortiz

Data type: Index of specimens examined

Explanation note: Index of collectors, collector numbers, and corresponding *curarea* species.

Specimens examined are listed alphabetically by main collector, the number in parenthesis corresponds to the taxa as cited in the text, and types are in boldface. Vouchers are marked with ^1^ for stem anatomy, ^2^ for leaf anatomy and stomata morphology observations, ^3^ for venation patterns, and ^4^ for trichome morphology.

Aguilar, R. 682^1^, 3303 (cuatrecasasii, ♂ fl).

Ancuash, E. 660^4^,1219^2^ (iquitana, ♂ fl).

**Anderson, s.n.** (candicans, type of synonym Sciadotenia leucophylla, sterile).

Angulo, L. 452 (cuatrecasasii, imm fr).

Araujo, A., et al. 3131 (tomentocarpa, mat fr).

Aronson, J. & F. Rodrigues 859 (barnebyana, old fr)

Aulestia, C. et al. 9 (gentryana, imm fr).

Aulestia, M. 1653 (aff. tomentocarpa, mat fr).

Barbosa Rodriguez, J. s.n. (Jardim Botanico do Rio do Janeiro 154967) (Curarea sp.).

Barbour, P.J. 5687 (tomentocarpa, imm fr).

Bell, D. & S. Wiser 88-113 (tomentocarpa, imm fr).

Berlin, B. 542 (iquitana, mat fr).

Brand, J. et al. 1283 (cf. cuatrecasasii, sterile).

Cabrera, H.W. et al. 95 (tomentocarpa, mat fr).

Callejas, R. 1183^2^ (cf. cuatrecasasii, imm fr), 3592 (cuatrecasasii, ♂ fl).

Campbell, D.G. et al. P21256 (cf. tecunarum, sterile).

Cárdenas, D. 374 (cuatrecasasii, imm fr), 723, 1018 (cuatrecasasii, mat fr), 1906, 2081 (cuatrecasasii, ♂ fl).

Carrillo, L. & D. Reyes 695 (aff. tomentocarpa, mat fr).

Carvalho, A.M.V. et al. 6031^3^ (crassa).

**Castelnau, M.D. s.n.** (toxicofera, type of Cocculus toxicoferus).

Castro, R. et al. 18929 (iquitana, imm fr).

Cerón, C.E. 2674, 2717 (both Curarea sp., sterile), 3324A (cf. tecunarum, sterile), 6008 (barnebyana, imm fr).

Cerón, C.E. & M. Cerón 4498 (aff. tomentocarpa, imm fr).

Cerón, C. & F. Coello 3237 (Curarea sp., sterile).

Cerón, C. & M. Factos 7453 (Curarea sp., sterile).

Cerón, C. & N. Gallo 4917, 5012 (Curarea sp., sterile).

Cerón, C.E & F. Hurtado 4094 (barnebyana, ♀ fl buds ).

Cerón, C.E. et al. 39668 (cf. toxicofera, sterile).

Cornejo, F. 2650 (cf. tomentocarpa, MO sheet sterile).

Coêlho, D. & L. Coêlho 41 (toxicofera, ♂ fl).

Croat, T.B. 18788 (toxicofera, mat fr).

Cuadros, H. & A.H. Gentry 3606 (cuatrecasasii, ♂ fl).

**Cuatrecasas, J. & L. Willard 26168** (type of Curarea cuatrecasasii, [imm?] fr).

Daly, D.C. et al. 6153 (tomentocarpa, mat fr), 6264 (tomentocarpa, mat fr), 6767 (tomentocarpa, imm fr), 7254 (tomentocarpa, mat fr), 8073 (tomentocarpa, imm fr), 9172 (tomentocarpa, ♂ fl), 9233 (tomentocarpa, imm fr), 10413 (tomentocarpa, mat fr), 11472 (tomentocarpa, imm fr).

Davis, E.W. & J. Yost 943 (cf. tecunarum).

de Nevers, G.C. & H. Herrera 10673 (cuatrecasasii).

de Nevers, G.C. et al. 5052, 5237, 5237 (cuatrecasasii, ♂ fl).

DeWalt, S. et al. 865 (tomentocarpa, imm & mat fr).

Diaz, F. 10 (cf. tecunarum, sterile), 36 (toxicofera, sterile).

Díaz, C. & N. Jaramillo 1238 (tecunarum, sterile), 1493 (toxicofera, mat fr).

Díaz, C. & J. Pereira 9091 (tomentocarpa, imm fr).

Díaz, C. et al. 1044 (tecunarum, sterile), 1187 (toxicofera, mat fr), 1291 (toxicofera, imm fr), 8239 (iquitana, imm fr).

Dik, A. 1213 (barnebyana, mat fr).

Dressler, R.L. 4372^2^ (cuatrecasasii, ♂ fl).

Duarte, A.P. 7964 (tecunarum, sterile).

Ducke, A. 1968 (toxicofera, ♂ fl buds), 2134 (toxicofera, ♂ fl).

Duke, J.A. 5208 (cuatrecasasii, imm fr).

Duque-Jaramillo, J.M. 2441 (toxicofera, ♂ fl).

Encarnación, F. 1094^2,4^ (toxicofera, ♂ fl).

Espina, J. et al. 2645 (cuatrecasasii, ♂ fl).

Folsom, J.P. et al. 6357 (cuatrecasasii, imm fr).

Forero, L.E. 620, 666 (cf. cuatrecasasii, sterile)

Foster, R. 2543 (tomentocarpa, mat fr), 2929 (cuatrecasasii, ♂ fl).

3722 (toxicofera, ♂ fl).

Foster, R. & C. Augspurger 3474 (tomentocarpa, mat fr).

Foster, R. & S. Baldeon 12669 (tomentocarpa, ♂ fl).

Foster, R. & J. Terborgh 6480 (tomentocarpa, mat fr).

Fox, M.W. 12 (Curarea sp., sterile).

Freire, B. & D. Naranjo 685 (cf. tecunarum, sterile).

Fróes, R. 12701/67 (crassa, sterile), 2087 (toxicofera, sterile), 12090 & 12092 (tecunarum, sterile), 12093(tecunarum, sterile), 21019 (toxicofera, ♂ fl); 21446 (tecunarum, ♀ fl); 21700 (tecunarum ♂ fl), 21709 (tecunarum, sterile), 21720 (tecunarum, sterile), 21802A (toxicofera, ♂ fl), 21836 (toxicofera, ♂ fl); 26364 (toxicofera, imm fr); 29639 (toxicofera, ♂ fl).

Funk, V.A. et al. 8213 (tomentocarpa, imm fr), 8360 (tomentocarpa, mat fr).

Galeano, G. et al. 1135 (toxicofera, imm fr).

Gaillard, M. 184 (toxicofera, ♂ fl).

García-Barriga, H. 14578 (toxicofera, sterile)

Garcia, F. 117^2^ (cuatrecasasii, mat fr).

Gentry, A.H. 5830 (cuatrecasasii, sterile), 70966 (cf. tomentocarpa, sterile), 76369 (cf. toxicofera, sterile).

Gentry A.H. & J. Aronson 24991 (tecunarum, sterile), 25126 (toxicofera, sterile).

Gentry, A.H. & F. Ayala 12699 (toxicofera, sterile).

Gentry, A.G. & C. Diaz 58512 (aff. tomentocarpa, mat fr).

Gentry, A.H. & L. Emmons 39633 (toxicofera, ♂ fl).

Gentry, A.H. & N. Jaramillo 57655 (cf. tomentocarpa, sterile).

Gentry, A.H. & C. Hamilton 41126^1^ (cuatrecasasii, ♂ fl).

Gentry, A.H. & R. Ortiz 78123^4^ (tomentocarpa, ♂ fl), 78233 (tomentocarpa, ♂ fl), 78315 (tomentocarpa, ♂ fl), 78318 (old ♀ fl), 78319 (tomentocarpa, ♂ fl).

Gentry, A.H. & B. Stein 46878 (cf. tecunarum, sterile).

Gentry, A.H. & K. Young 31927 (tomentocarpa, imm fr).

Gentry, A.H. et al. 18505 (toxicofera, ♂ fl), 21624 (toxicofera, ♀ fl), 25720 (aff. tomentocarpa, ♂ fl), 25991 (tecunarum, sterile), 26990 (tomentocarpa, mat fr), 27080 (tomentocarpa, ♀ fl), 28944 (toxicofera, mat fr), 42061 (cf. tecunarum, 42157 (cf. toxicofera, mat fr), 51533 (tomentocarpa, mat fr), 54452 (tecunarum, sterile), 58156 (tomentocarpa, imm fr), 60167 (Curarea sp., sterile), 63375 (cf. tecunarum, sterile), 76977 (cf. tomentocarpa, sterile), 77256 (cf. tomentocarpa, imm fr), 77414 (tecunarum, sterile), 79361 (cuatrecasasii, sterile).

Glaziou, A. 9610 (@ P, cf. tecunarum, @ F = toxicofera).

Goulding, M. 1187^2^ (toxicofera, ♂ fl).

Graham, J.G. & J. Schunke 352 (toxicofera, ♂ fl).

Grández, C. 3475 (cf. toxicofera, sterile)

Grández, C. et al. 1618 (cf. toxicofera, imm fr), 3549 (tecunarum, sterile), 5028 (tecunarum, sterile).

Grassl, C.O. 10076 (toxicofera, sterile).

**Gudiño, E. et al. 952** (type of Curarea barnebyana, ♂ fl).

Hahn, W.J. & S. Tiwari 5136 (candicans, imm fr)

Hammel, B.E. et al. 18912 (cuatrecasasii, sterile).

Holm-Nielsen, L. et al. 22169 (toxicofera, mat fr).

Hoover, W.S. et al. 4530 (gentryana, imm fr).

Huber 4286 ( aff. tomentocarpa).

Huamán, M. et al. 417 (cf. tecunarum, sterile).

Jansen-Jacobs, M.J. et al. 1995^2,3,4^ (candicans, ♂ fl).

Jaramillo, N. et al. 308 (iquitana, imm fr).

Jardim Botanico do Rio do Janeiro RB-19508 (candicans, detached fr).

**Jardim, J.G. et al. 351**^2^ (type of Curarea crassa), 609^2,4^ (crassa, old ♀ fl).

Jenman 5199 (candicans, ♂ fl).

Kayap, R. 156 (iquitana, mat fr), 749 (iquitana, mat fr), 1032 (iquitana, imm fr), 1205 (iquitana, mat fr).

Kennedy, H. 1640 (cuatrecasasii, imm fr).

Kernan, C. & P. Phillips 1147^2,4^ (cuatrecasasii, ♂ fl).

Killip, E.P. & A.C. Smith 28665 (toxicofera, ♂ fl), 29337 (cf. toxicofera, sterile).

King, S. et al. 972 & 977 (cf. tecunarum, sterile).

Klug, G. 2042, 2782 (toxicofera, ♂ fl).

Knapp, S. & J. Mallet 4706 (cuatrecasasii, ♂ fl).

**Krukoff, B.A.** 4754 (toxicofera, sterile), 7535 (tecunarum, sterile), 7578 (tecunarum, ♂ fl bds), 7823, 7824, 7826 (tecunarum, sterile), 8522 (♂ fl), 7828 (tecunarum, sterile), 8549 (tecunarum, ♂ fl), 8370, (tecunarum, ♂ fl), **8713**, (type of Curarea tecunarum, ♂ fl), 10799 (aff. tomentocarpa, sterile), 12305, 12335 (candicans, sterile).

Kujikat, A. 107 (iquitana, old ♀ fl and imm fr).

Lanjouw, J. & J.C. Lindeman 2775a, 2779^1,3^ (candicans, sterile).

Lewis, W. & R. Vásquez 4017 (toxicofera, imm fr).

Lewis, W.H. et al. 10224 (tecunarum, sterile), 10425, 11759 (tecunarum, sterile), 13848 (tecunarum, sterile), 14349 (tecunarum, sterile), 18475 (iquitana, [imm?] fr).

Liesner, R.L. 1325 (cuatrecasasii, mat fr).

Lindeman, J.C. & A.C. Roon 808 (candicans, sterile).

Lindeman, J.C. et al. 539^4^, 805 (candicans, sterile).

Lowrie, S.R. et al. 595 (tomentocarpa, ♂ fl).

Madriñán, S. 702 (toxicofera, ♂ fl).

Marín. J. 246 (cuatrecasasii, mat fr).

Marinho, L.R. 291 (toxicofera, imm fr).

Martin, R.T. & C.A. Lau-Cam 1204 (cf. tecunarum, sterile), 1266 (toxicofera, sterile), 1273 (Curarea sp, cf. tecunarum, sterile).

Martin, R. & T. Plowman 1798 (toxicofera, sterile).

**Martius s.n.** (candicans, type of Cocculus dichroa)

Mathias, M.E. & D. Taylor 3555 (cf. tecunarum, sterile), 3900 (toxicofera, imm fr), 5010, 5018, 5088 (cf. tecunarum, sterile).

McDaniel, S. & M. Rimachi 23886 (toxicofera, ♂ fl).

McPherson 7956 (cuatrecasasii, imm fr).

McPherson, G. & M. Merello 8175 (cuatrecasasii, mat fr).

Miller, J.S. et al. 684 (Curarea sp., sterile).

Morales, J.F. et al 3243^3^ (cuatrecasasii, mat fr).

Mori, S. 7946 (cuatrecasasii, imm fr).

Mori, S. & J. Kallunki 3695 (cuatrecasasii, imm fr).

Mori, S. et al. 22743 (candicans, old ♂ fl).

Moya, G. & D. Reyes 274 (cf. tecunarum, sterile).

Nabe-Nielsen, J. 17 (Curarea sp., sterile).

Naranjo, C. 7 (cf. tecunarum, sterile).

Naranjo, C. & G. Wiederhold 16 (cf. tecunarum, sterile).

Naranjo, D. & B. Freire 363 (cf. tecunarum, sterile), 635 (cf. tecunarum, sterile).

Nee, M. 7731 (cuatrecasasii, imm & mat fr).

Neill, D. 8705 (mat fr), 8712 (barnebyana, old ♀ fl).

Neill, D. et al. 7806 (aff. tomentocarpa, [imm?] fr), 8679 (Curarea sp., sterile).

Nelson, B. 606 (tomentocarpa, ♂ fl), 716 (tomentocarpa, mat. fr).

Núñez, P. 5631 (tomentocarpa, mat fr), 11154 (tomentocarpa, sterile).

Núñez, P. & G. Ortiz 12765 (tomentocarpa, sterile).

Ortiz, R. 158, 159^4^ (cf. toxicofera, imm fr), 176 (aff. iquitana, old ♂ fl), 177 (aff. iquitana, imm fr), 181^1^ (aff. iquitana, old ♂ fl), 184 (aff. iquitana, ♀ fl), 185^2,3^ (aff. iquitana, ♂ fl), 186^1^ (aff. iquitana, imm & mat fr).

Ortiz, R. & G. Cahuamari 273 (toxicofera, ♂ fl).

Ortiz, R. & N. Pezo 208^4^ (aff. iquitana, ♀ fl and mat fr).

Ortiz, R. & J.C. Ruiz 187, 188^4^ (tecunarum, ♂ fl), 191 (aff. iquitana, imm fr).

Ortiz, R. & J. Vargas 194^2,3,4^ (barnebyana, imm fr), 195 (barnebyana, old ♀ fl & imm fr), 197^1^ (aff. tomentocarpa, imm fr), 199^1,2,3^ (aff. tomentocarpa, ♂ fl), 200^1,2,3,4^ (barnebyana, old ♀ fl & imm fr).

Ortiz, R. & R. Vásquez 209 (tecunarum, ♂ fl), 212 (tecunarum, sterile), 214^1^ (tecunarum, sterile).

Ortiz, R. et al. 140, 141 (tecunarum, sterile), 143^2^ (tecunarum, imm fr), 144 (tecunarum, old ♂ fl), 157^4^ (toxicofera, ♂ fl), 166 (cf. toxicofera, sterile), 167 (aff. iquitana, ♂ fl), 168 (aff. iquitana, ♂ fl), 169 (aff. iquitana, ♂ fl), 170 (aff, iquitana, old ♂ fl), 171 (aff. iquitana, old ♂ fl) , 172 (aff. iquitana, imm & mat fr), 173 (aff. iquitana, old ♂ fl), 220^1^ (tecunarum, sterile), 230 (aff. iquitana), 290 (cf. tecunarum, sterile).

Palacios, W. 3106^4^ (aff. tomentocarpa, ♂ fl), 7504 (aff. tomentocarpa, ♂ fl), 10725 (aff. tomentocarpa, mat fr).

Palacios, W. & E. Freire 5104 (aff. tomentocarpa, mat fr).

Palacios, W. et al. 881 (aff. tomentocarpa, ♂ fl), 890 (aff. tomentocarpa, imm fr), 9032, 9493 (toxicofera, imm fr).

Paniagua, N. & S. Beck 1171 (tomentocarpa, mat fr).

Paniagua, N. et al. 5029 (tomentocarpa, mat fr).

Paz y Miño, G. 81.077, 81.048 (Curarea sp., sterile).

Peña, B.S. 547 (toxicofera, [imm?] fr).

Perry, A. et al. 413 (tomentocarpa, mat fr), 654 (tomentocarpa, [sterile?]).

Pipoly, J.J. & R. Boyan 8982^2^ (candicans, ♂ fl).

Pipoly, J.J. et al. 12846 (tecunarum, ♂ fl), 16059 (cf. toxicofera, sterile).

Pinkley, H.V. 285 (cf. tecunarum, sterile), 392 (cf. tecunarum, sterile).

Plowman, T. 7712 (cf. toxicofera, sterile).

Plowman, T. et al. 4029 (aff. tomentocarpa, mat fr), 12587 (cf. tecunarum, sterile).

Prance, G.T. et al. 11272 (toxicofera, ♂ fl), 13931 (cf. tecunarum, sterile), 16146 (tecunarum ♂ fl), 16453 (tecunarum, sterile).

**Pulle 408** (candicans, type of synonymous Abuta? pullei).

Renteria, E. 3754, 4749 (cuatrecasasii, both ♂ fl).

Renteria, E. & D. Cárdenas 4363 (cuatrecasasii, imm fr).

Revilla, J. 445 (toxicofera, ♂ fl), 3779A (toxicofera, ♂ fl).

Revilla, J. & E. Carrillo 1501 (toxicofera, mat fr).

Revilla, J. et al. 2566 (toxicofera, imm fr).

Reyes, D. & L. Carrillo 827 (aff. tomentocarpa, mat fr).

Reynel, C. & E. Meneses 5025^2^ (tomentocarpa, imm fr).

Reynel, C. et al. 5330 (tomentocarpa, mat fr).

**Richard, L.C., s.n.** (candicans, type of Abuta candicans), s.n. (candicans, sterile).

Rimachi, M. 10503 (toxicofera, mat fr, MO sheet sterile), 10636 (toxicofera, mat fr), 11394 (tecunarum, ♂ fl).

**Rodr. Sigueira s.n.** [Herbario Amazonico Museo Paraense-8266] (candicans, type of synonymous Abuta limaciifolia).

Rojas, R. 7674 (aff. iquitana, mat fr), 7909 (aff. iquitana, imm fr).

**Rubio, D. & C. Quelal** 1434^2,4^ (gentryana, mat fr), **1503**^2,3,4^ (type of Curarea gentryana).

Rudas, A. et al. 1528 (toxicofera, imm fr), 1708 (tecunarum, sterile), 2512 (toxicofera, ♂ fl), 2825 (toxicofera, mat fr), 3636, 4166, 5702 (tecunarum, all sterile).

Sandwith, N.Y. 561 (candicans, ♂ fl).

Schultes, R.E. 3522 (Curarea sp., sterile), 5526 (toxicofera, mat fr, n.v.).

Schultes, R.E. & F. Lopez 10400g (tecunarum, sterile).

Schunke, J. 1970-24 (cf. tecunarum, sterile), 2599 (aff. tomentocarpa, mat fr), 2690^4^,(aff. tomentocarpa, ♂ fl), 2981 (aff. tomentocarpa, mat fr), 3830 (aff. tomentocarpa, ♂ fl), 4605 (aff. tomentocarpa, imm fr), 5848, 5849, 5850, 5851 (cf. tecunarum, all sterile), 6836 (aff. tomentocarpa, ♂ fl), 7125 (aff. tomentocarpa, ♂ fl), 7479 (aff. tomentocarpa, sterile).

Schunke, J. & J.G. Graham 15323 (toxicofera, ♂ fl), 16307 (cf. barnebyana, ♂ fl).

Schwacke 3465 (cf toxicofera, sterile)

Silva, M.G. 6538 (tecunarum ♂ fl).

Silverwood-Cope, P. 23 (cf. tecunarum, sterile).

Smith, D.N. 6584^2^ (aff. iquitana, mat fr).

Smith, D.N. et al. 12882 (tomentocarpa, mat fr).

Smith, S.F. et al. 320 (tomentocarpa, mat fr), 1424, 1467 (both tomentocarpa, ♂ fl), Solomon, J.C. 6317 (tomentocarpa, mat fr).

Soto, R. 4116 (cuatrecasasii, mat fr).

Stergios B. et al. 3367 (Curarea sp.)

Stern, W.L. et al. 374 (cuatrecasasii, ♂ fl).

Steyermark, J. et al. 125686 (cf. candicans, ♂ fl buds).

**Tessmann, G. 4196** (iquitana, type of Chondrodendron iquitanum).

Thomas, W.W. 6753 (toxicofera, ♂ fl).

Thomas, W.W. et al. 10900 (crassa, ♂ fl).

Timaná, M. 1945^5^ (tomentocarpa, ♂ fl), 1978 (tomentocarpa, imm fr).

Timaná, M. & N. Jaramillo 3319 (tomentocarpa, ♂ fl).

Timaná, M. & A. Rubio 2084^3^ (tomentocarpa, mat fr).

Tina, F. & Oliveira 2343 (cf. tecunarum, sterile).

Tina, F. & M. Tello 2066 (toxicofera, ♂ fl).

Torke, B. et al. 310 (candicans, sterile)

Ubiratan, J. et al. 218 (tecunarum, imm fr).

*unknown* (U-32454-B!) (candicans, sterile).

**Ule, E. 5631** (toxicofera, type of synonym Hyperbaena polyantha).

Valenzuela, L. & J. Farfan 2243 (tomentocarpa, ♂ fl buds).

Valenzuela, L. & I. Huamantupa 975 (tomentocarpa, ♂ fl).

van der Werff, H. & R. Vásquez 13909^1,2,3,4^ (tecunarum, imm fr), 13990^2,3^ (toxicofera, ♂ fl).

Vásquez 16803 (cf. tecunarum, sterile), 17898 (tecunarum, sterile), 19467 (cf. tecunarum, sterile), 22914 (tecunarum, sterile), 25238 (tecunarum, sterile).

Vásquez, R. & G. Criollo 5790 (aff. iquitana, ♀ fl [and mat fr]).

Vásquez, R & N. Jaramillo 43 (toxicofera, ♂ fl), 3539 (aff. iquitana, imm fr), 11714 (toxicofera, ♂ fl), 12907 (aff. iquitana, imm fr), 12982 (aff. iquitana, imm fr), 15101 (tecunarum, sterile), 15588 (aff. iquitana, imm fr) , 16136 (toxicofera, ♂ fl), 16361 (tecunarum, sterile).

Vásquez, R. et al. 456 (toxicofera, ♂ fl), 4884 (toxicofera, sterile), 5941 (aff. iquitana, ♂ fl), 6602 (aff. iquitana, mat fr), 10689 (aff. iquitana, imm fr), 12001 (aff. iquitana, imm fr), 18225 (aff. iquitana, mat fr),18715^2,4^ (iquitana, old ♂ fl), 18992ª (iquitana, imm fr), 18994^3^ (iquitana, ♂ fl), 21737 (iquitana, mat fr).

Vreden 11663,11706 (candicans, sterile).

Weiss, G. 132 (toxicofera, sterile).

White, O.E. 1812 (tomentocarpa, type of Abuta boliviana**.**

**Williams, R.S.** 562 (tomentocarpa); **616** (tomentocarpa, type of Cissampelos tomentocarpa.

Woytkowski, F. 108, 5108 (cf. toxicofera, both sterile), 5336 (cf. tecunarum, sterile), 5354 [2] (cf. tecunarum, sterile), 5355 (cf. tecunarum, sterile).

Zavortink, T.J. 2297 (toxicofera, sterile), 2301 (cf. toxicofera, sterile).
